# Microwave-Assisted Synthesis of Morpholine-Based Chalcones as Reversible MAO-A Inhibitors in the Management of Mental Depression

**DOI:** 10.3390/ph18030309

**Published:** 2025-02-23

**Authors:** Diksha Choudhary, Bhupinder Kumar, Balakumar Chandrasekaran, Thakur Gurjeet Singh, Rajwinder Kaur, Afaf Aldahish, Rajalakshimi Vasudevan, Prasanalakshmi Balaji

**Affiliations:** 1 Chitkara College of Pharmacy, Chitkara University, Rajpura 140401, Punjab, India; 2 Department of Pharmaceutical Sciences, HNB Garhwal University, Chauras Campus, Srinagar 246174, Jammu and Kashmir, India; 3 Faculty of Pharmacy, Philadelphia University, P. O. Box 1, Amman 19392, Jordan; balakumar@philadelphia.edu.jo; 4 Department of Pharmacology, College of Pharmacy, King Khalid University, Abha 61421, Saudi Arabiaraja@kku.edu.sa (R.V.); 5 Department of Computer Science, College of Computer Science, King Khalid University, Abha 61421, Saudi Arabia

**Keywords:** depression, neurotransmitters, in silico, monoamine oxidase, mental health, hMAO-A

## Abstract

**Background:** Depression is one of the most serious and common health problems among the youth population and is responsible for the initiation of many diseases. As per the World Health Organization, 3.8% of the population suffers from mental depression, globally. The monoamine oxidase-A (MAO-A) enzyme is responsible for the degradation of neurotransmitters leading to lower levels of neurotransmitters. **Methods**: Chalcones (C1-C15) were synthesized by reacting substituted acetophenone with various benzaldehydes in a basic ethanolic solvent at 80 °C under microwave irradiation conditions. To compare the reaction time and product yield, a conventional method of synthesis of chalcones was also performed. The synthesized chalcones (C1-C15) were spectroscopically characterized and screened initially for inhibitory activities against MAO-A and MAO-B. The best active compounds were undertaken for IC_50_ determination against MAO-A enzyme followed by the reversibility of inhibition analysis and the antioxidant assay. Moreover, in silico molecular docking and ADME pharmacokinetic investigations were accomplished. **Results**: Most of the compounds inhibited MAO-A, specifically, compounds C14 and C6 exhibited the highest inhibition at IC_50_ values of 7.91 ± 0.08 μM and 8.45 ± 0.19 μM, respectively. Both these compounds exhibited a reversible MAO-A inhibition displaying up to 60% recovery of enzymatic activity when diluted with substrate (Tyramine). The results of the in silico study indicated docking scores of −9.56 Kcal/mol (C14) and −9.45 Kcal/mol (C6) and exhibited a π-π stacking interaction with the crucial amino acid Trp-397. The compounds were determined to cross the blood–brain barrier (BBB) and displayed favorable gastrointestinal (GI) absorption. Further, the antioxidant assay results demonstrated that the synthesized compounds possess modest free radical scavenging potential. **Conclusions**: This study displayed the MAO-A inhibitory potential of morpholine-substituted chalcones as a promising pharmacophore for the development of novel antidepressant lead compounds.

## 1. Introduction

Depression or major depressive disorder is the most serious mental disorder affecting humans characterized by low mood and aversion to activity [1]. As per the World Health Organization (WHO) data, around 3.8% of the population suffers from depression, and 280 million all over the world are affected by this mental disorder, which includes 5% of adults (4% of men and 6% of women) and 5.7% of adults older than 60 years of age [2]. Depression is about 50% more common among women than men [3]. Worldwide, among pregnant and post-delivery women, 10% are suffering from mental depression [4]. More than 700,000 people die due to suicide every year. Suicide is the fourth leading cause of death among the age group between 15 and 29 years [5]. Depression is associated with a loss of interest in daily life activities and sudden sad feelings, unlike regular mood alterations that indicate a mood disorder in depressed patients [6]. The monoamine oxidases (MAOs) are a family of enzymes that play a crucial role in the degradation of monoamine group-containing neurotransmitters by catalyzing oxidation reactions [7]. Two isoforms of MAO have been identified, namely MAO-A and MAO-B. They differ regarding affinity, substrate specificities, tissue distributions, and relative expressions [8]. MAO-A has an affinity towards monoamines such as serotonin and norepinephrine, whereas MAO-B has a higher affinity to phenylethylamine and benzylamine leading to their metabolism. Both isoforms can metabolize other monoamine-bearing neurotransmitters such as dopamine, tyramine, epinephrine, and tryptamine [9]. Inhibitors of MAO-A are useful as antianxiety and anti-depressant agents [10], while MAO-B inhibitors are clinically useful in the treatment of Parkinson’s disease [11] and Alzheimer’s Disease [12]. Thus, MAO-A and B or mixed MAO-A/B emerged as crucial enzyme targets to treat neurological diseases [13]. From the review of the literature, it is clear that the major drawback of currently available antidepressant drugs including monoamine oxidase inhibitors (MAOIs) is ineffectiveness in the treatment [14]. Several side effects are associated with currently employed MAOIs such as indigestion, diarrhea, constipation, loss of appetite, dizziness, insomnia, dry mouth, blurred vision, drowsiness, weight gain, excessive sweating at night, arrhythmia, agitation, and seizures [15]. Literature reports indicated that MAOIs are responsible for such side effects mainly due to their irreversible and non-selective nature of binding to MAO [16]. These problems are related to antidepressant drugs and can be addressed by designing target-specific compounds that can inhibit the MAO selectively.

From the exploration of the crystal structure of the MAO isoenzymes, it has been observed that both are dimeric in membrane-bound form having identical exon–intron arrangements sharing nearly 70% amino acid similarity [17]. Further, the binding pocket of the active site amino acids in human MAO-B (hMAO-B) is longer because of the presence of the bipartite cavity compared to the monopartite cavity in human MAO-A (hMAO-A) [18]. For hMAO-A, the active site crucial amino acid residues are Tyr-69, Gln-74, Val-91, Val-93, Leu-97, Ile-180, Ile-207, Phe-208, Ser-209, Val-210, Glu-216, Cys-323, Ile-325, Ile-335, Leu-337, Met-350, Trp-397, Tyr-407, and Tyr-444 [19].

**Figure 1:**
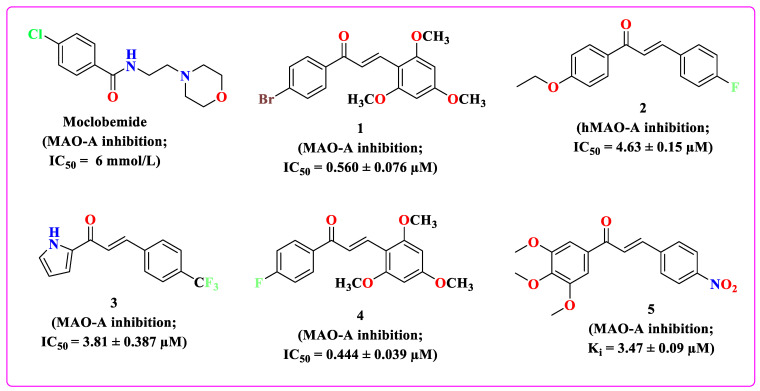
Chalcone-based MAO-A selective inhibitors including morpholine hybrid drug moclobemide. All heteroatoms [Chlorine, Cl = green (dark); Fluorine, F = green (light); Bromine, Br = Brown; Nitrogen, N = blue (dark); and Oxygen, O = Red] are colored according to the standard CPK rule.

Chalcones are a highly prolific class of bioactive pharmacophores present in the structures of drugs and medicinal compounds [20]. Chalcones and their hybrids have been reported for their antifungal [21], antiplatelet [22], antidepressant [23], analgesic, anti-inflammatory [24], antileishmanial [25], antidiabetic [26], antimalarial [27], anti-cancer [28], antibacterial [29], antioxidant [30], antiparasitic [31], and antitubercular [32] activities. Figure 1 presents the literature-reported chalcone-based MAO-A selective inhibitors including the morpholine-containing selective MAO-A inhibitory drug moclobemide [33,34,35,36,37]. Researchers have explored the morpholine-based chalcones for MAO and acetylcholinesterase (AchE) inhibition [38,39]. The idea of hybridizing the morpholine ring to the chalcone is based on the literature that reported MAO inhibitors and the standard drug moclobemide bearing a morpholine ring which plays a crucial role in binding affinity and MAO-A selectivity [40]. Further, the morpholine ring enhances the solubility parameter and provides good permeability in the brain [41] which is a key challenge common to drugs targeting the neurological system.

Based on the above literature findings, we have designed and synthesized a series of morpholine-containing chalcones to explore their potential for reversible MAO-A inhibition aimed at treating mental depression without exerting any serious side effects. We herein reported the synthesis of these morpholine-containing chalcones by a microwave (MW)-assisted method for the first time. The synthesis of chalcones was carried out using both conventional methods (CMs) and non-conventional MW-assisted reactions. MW irradiation, the efficient and ecological-friendly heating method, was found to be the most reliable technique for activating fine chemicals in chemical reactions, leading to faster reaction times, higher conversion rates, and more selectivity [42]. The CM has been well explored in laboratories, but compared to MW-assisted reactions, it is a time-consuming process and requires the use of more quantities of solvents for reactions [43]. Additionally, the chances of the formation of side products are higher in CMs. In contrast, MW-assisted reactions involve direct and rapid heating, reducing the likelihood of side product formation and leading to a higher product yield in less time compared to CMs [44].

The primary novelty of our work is to obtain the target compounds in higher yield using the MW-assisted organic synthesis method and to explore their hMAO inhibitory potential. Further, we have targeted the enzyme MAO-A with selective inhibition that is reversible, to treat mental depression, more effectively. In silico studies assist in exploring the binding orientation of the drug molecules to their corresponding target proteins and understanding the crucial active site amino acids in ligand binding and selectivity. Hence, a molecular docking study was conducted using the X-ray-solved crystal structure of MAO-A. ADME pharmacokinetic prediction allowed us to determine the drug-likeliness of the synthesized compounds. It is also important to note that reactive oxygen species (ROS) are the by-products of MAO enzymatic activity, are responsible for oxidative stress, and are involved in mental depression. Accordingly, we are also interested in determining the antioxidant potential of our synthesized chalcones using a 1,1-diphenyl-2-picryl hydrazyl (DPPH) assay.

## 2. Results and Discussion

### 2.1. Chemistry

**Scheme 1:**
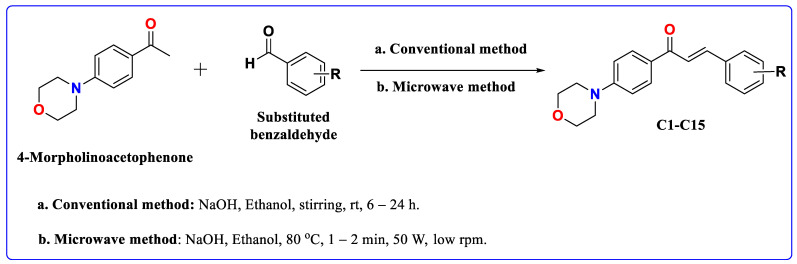
Synthetic route to obtain the titled compounds C1-C15. Heteroatoms [Nitrogen: N = blue (dark) and Oxygen: O = Red] are colored according to the standard CPK rule.

The chalcones were synthesized via a well-known Claisen–Schmidt condensation reaction [45] under CM and MW-assisted methods (Scheme 1). An equimolar mixture of 4-morpholinoacetophenone and substituted benzaldehydes was reacted under basic conditions to yield the target compounds (C1-C15). A non-conventional method involving MW irradiation-assisted synthesis of the compounds was employed to compare the reaction time and product yield. To date, this is the first time reporting the MW method of these target compounds. The completion of each reaction was monitored using thin-layer chromatography (TLC) using a solvent system with a mixture of *n*-hexane–ethyl acetate (7:3). Wang et al. reported the synthesis of chalcones using CM through Claisen–Schmidt condensation between substituted acetophenone and benzaldehyde in the presence of 40% KOH in ethanol at room temperature for 12 h [46]. For the first time, we report the synthesis of the titled chalcones using CM and MW-assisted methods under modified conditions.

The structures of all the synthesized compounds were established based on spectral characterization using FTIR, ^1^H NMR, ^13^C NMR, and HRMS. The FTIR spectra of the chalcones indicated the presence of α,β-unsaturated carbonyl (C=O) stretching which appeared in the range of 1640–1655 cm^−1^, and C=C bond stretching was observed between 1560 and 1650 cm^−1^ in the structures of target chalcones. All the synthesized compounds show a new –C=C– bond formation between α and β carbons conjugated to the carbonyl group indicating the formation of chalcone. The appearance of two proton signals in the range of δ 7.40–7.55 (α-H) ppm and δ 7.67–7.80 (β-H) ppm, respectively, in the ^1^H NMR spectrum confirmed the formation of the chalcone. The *E*-configuration of the synthesized chalcones was determined based on the values of the coupling constant (*J*). If the *J* value is more than 15 Hz, then it is *E*-selective, wherein a *J* value of less than 12 Hz is attributable to the *Z*-configuration. In all of our final compounds, we observed a *J* value of 15 Hz for 14-α and 15-β protons that confirmed the *E*-configuration. In the ^1^H NMR spectrum of C1-C15, the CH_2_ signals of the morpholine ring appeared at δ 3.31–3.32 ppm and δ 3.75–3.88 ppm as triplets, respectively, as reported in the literature [38,39]. In ^13^C NMR, the carbonyl carbon appeared in the deshielded region with δ between 188.0 and 187.7 ppm, confirming the formation of the α, β-unsaturated carbonyl system of the chalcones. Moreover, the HRMS of all the final compounds displayed the presence of molecular ion peaks as expected which further confirmed their chemical structures.

The characterization data of the non-novel compounds C1, C3, C7, C9, C10, and C13 are corroborated well with the compounds reported in the literature [38,39]. However, the authors did not report the FTIR spectra of their synthesized compounds. Further, the authors screened their chalcones for MAO-B selective enzyme inhibition, while we designed and explored the chalcones for MAO-A selective enzyme inhibition potential, which plays a crucial role in mental depression. In this research, the characterization of the novel compounds (C2, C4–C6, C8, C11, C12, C14, and C15) was established based on all the spectral data. Table 1 compares the reaction time and percentage yields obtained in CM and MW methods, whereas Table 2 illustrates the physical characterization data of the synthesized chalcones (C1–C15). The supplementary material provides all the spectral images of FTIR, ^1^H NMR, ^13^C NMR, and HRMS of the synthesized compounds (Figures S1–S58).

### 2.2. Biological Assays

#### 2.2.1. MAO Enzymes Inhibition Assay

Since MAO enzymes are implicated in the degradation of neurotransmitters, the MAO enzymes inhibition assay was carried out to validate the inhibition potential of the synthesized chalcones C1–C15. The enzyme inhibition assay was performed using an Amplex Red assay kit using fluorometric techniques to assess the hMAO inhibitory activity of each synthesized compound [47]. Initially, the inhibitory potential of all synthesized compounds was evaluated at a concentration of 20 μM. The compounds that showed greater than 50% inhibition were further evaluated for the IC_50_ determination. The IC_50_ values against MAO enzymes were determined for only those compounds that showed a good percentage of inhibition at the highest concentration (20 μM). Table 3 presents the percentage inhibition of all the compounds against MAO isoforms and selective IC_50_ values. Compounds C14 and C6 displayed the most potent MAO-A inhibition activity with IC_50_ values of 7.91 ± 0.08 μM and 8.45 ± 0.19 μM, respectively. The compound C12 also displayed potential for MAO-A inhibitory activity with an IC_50_ value of 9.35 ± 0.11 μM. These results demonstrated that morpholine-based chalcones possess moderate selective MAO-A inhibitory potential.

#### 2.2.2. Reversibility Inhibition Study

**Figure 2:**
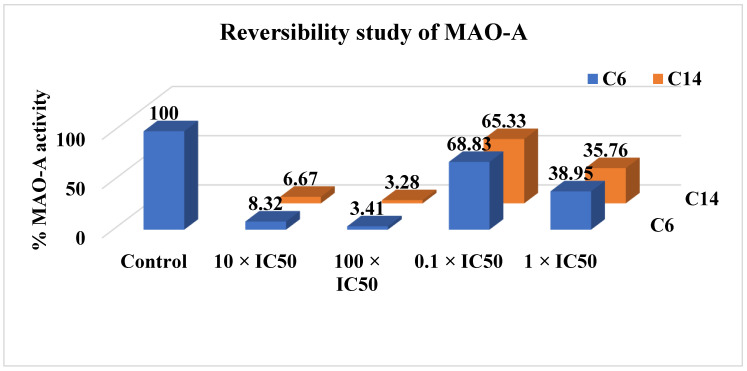
Reversible inhibition profile of compounds C6 and C14 against hMAO-A (100× IC_50_ and 10× IC_50_).

The reversibility study was carried out to validate the potential of the compounds to bind and temporarily inhibit the enzyme. The reversibility study [48] was conducted for the most potent compounds (C6 and C14) against the hMAO-A enzyme and identified that they reversibly inhibit the hMAO-A enzyme. The activity of the enzyme decreases to a minimum upon treatment with the test compounds at a concentration of 10× IC_50_ and 100× IC_50_. Upon 100 times dilution with substrate solution, recovery of more than 60% of the enzymatic activity was observed (Figure 2) which indicates that these compounds are reversible inhibitors of MAO-A and can be displaced from the binding cavity of MAO-A as soon as the concentration of substrate increases.

#### 2.2.3. Structure–Activity Relationship (SAR) Study

**Figure 3:**
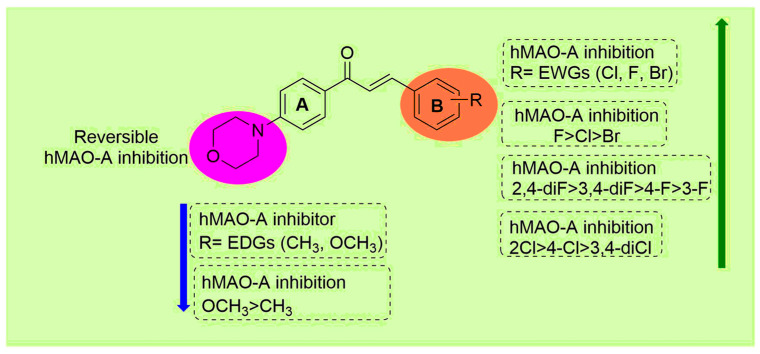
Structure–activity relationship (SAR) study.

In this series, we have synthesized chalcones containing morpholine (ring A) and various substitutions at the ortho, meta, and para positions of ring B. The synthesized compounds were evaluated at different concentrations for their inhibitory effects on hMAO-A and hMAO-B enzymes. Based on the percentage inhibition values, we found that the inhibitory effect on hMAO-A was most pronounced when the ring B was substituted with electron-withdrawing groups (EWGs). Compounds C4, C8, C9, C11, C13, and C15 substituted with electron-donating groups (EDGs) exhibited the lowest percentage inhibition of the hMAO-A enzyme. Notably, the presence of a methoxy group at the ortho position (C11) demonstrated superior inhibition of MAO-A compared to methyl substitution (C8, C9, C13, and C15). However, methoxy groups at the meta and para positions (C4) resulted in reduced inhibition compared to the ortho position (Figure 3).

Halogen substitutions at various positions (C1, C2, C3, C5, C6, C7, C10, C12, and C14) displayed better activity than methyl, ethyl, and methoxy (C4, C8, C9, C11, C13, and C15) substitutions. Specifically, the chlorine at the ortho position (C12) achieved a notable percentage inhibition of 67.7%. Conversely, shifting the chlorine to the para position (C7) resulted in a marked decrease in inhibition, and chlorine substitutions at the meta and para positions (C2) were detrimental to the inhibition. The 2,4-difluoro compound (C14) exhibited the highest percentage inhibition at 75.4%, while the 3,4-difluoro (C6) demonstrated nearly equal inhibition of 71.6% at 20 μM concentration. Overall, halogen substitutions at the ortho and/or para positions crucially contributed to enhancing MAO-A inhibition, while substitutions at the meta position tend to decrease the inhibition.

#### 2.2.4. Antioxidant Activity Assay

H_2_O_2_ produced during metabolic events in the brain may be crucial for signaling and metabolic functions [49]. The compounds were screened to check their potential to inhibit ROS through the DPPH assay [50]. The results of the assay (Table 4) showed that some of the compounds exhibited a 50% antioxidant activity at a 75 μM concentration (C1, C2, C3, C6, C7, and C14) and a 100 μM concentrations (ascorbic acid, C1, C2, C3, C4, C5, C6, C7, C8, C13, and C14). These chemicals demonstrate antioxidant capacity in the DPPH assay, suggesting their potential to protect neuronal cells from ROS. However, further studies are required to confirm this effect.

### 2.3. Molecular Docking Study

**Figure 4:**
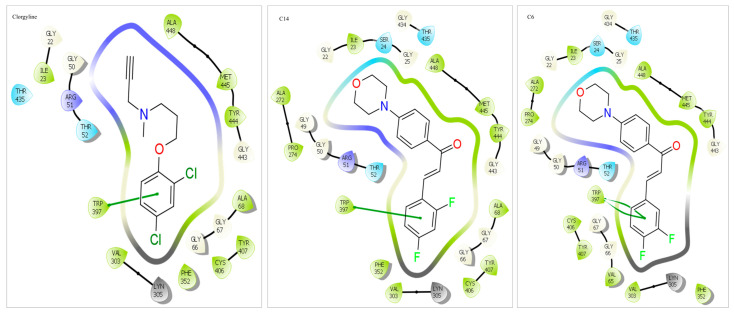
Docking poses of clorgyline, C14, and C6 in the active site of the hMAO-A enzyme.

A molecular docking study was conducted to understand the compounds’ critical interactions and orientation with different amino acids present in the active site of MAO-A. All synthesized compounds were subjected to docking studies using the Glide module with the interface Maestro 12.5 (Schrödinger Inc., New York, NY, USA). The compounds were docked to the active site of the enzyme (PDB ID: 2Z5X) [51]. The molecular interactions between the best active ligands and active site amino acid residues of MAO-A are given in Figure 4. The active site of the MAO-A enzyme consists of specific amino acid residues, like Tyr-69, Asn-181, Phe-208, Val-210, Gln-215, Cys-323, Ile-325, Ile-335, Leu-337, Phe-352, Tyr-407, and Tyr-444 which are essential for the substrate binding and catalysis. The phenyl ring of chalcone in C14 and C6 showed a π-π stacking interaction with Trp-397 which is similar to the interaction of the standard ligand clorgyline (Figure 4). The docking data showed that all of the ligands have a substantial binding affinity for the hMAO-A enzyme. The docking score of all the compounds was collected in Table 5.

### 2.4. ADME Pharmacokinetics Study

The ADME study of the compounds showed that all the compounds follow the Lipinski rule of five, i.e., all the compounds have a molecular weight of less than 500 Da, the number of hydrogen bond acceptors is less than 10, and the number of hydrogen bond donors is also less than 5. The log P value of all the compounds must be less than five for all drug-like compounds [52]. Table 5 presents the ADME pharmacokinetics data of all the target compounds. The results indicated that all compounds complied with Lipinski’s rule of five and exhibited drug-like properties. This has been further supported by a Log P of less than 5, showing good GI absorption and better penetration of the BBB. The higher bioavailability score of the compounds demonstrated good absorption.

## 3. Material and Methods

### 3.1. General Information

All the solvents and chemicals were obtained from Sigma-Aldrich (Sigma-Aldrich Chemicals Private Limited, Bangalore, India) of analytical grade and used without purification. The reactions were monitored using pre-coated thin-layer chromatography (TLC) Aluminum sheets G/UV 254 (Loba Chemie Private Limited, Mumbai, India) under the UV cabinet. CEM Discover (CEM Scientific India Private Limited, Hyderabad, India) was used to perform the microwave-assisted reactions. Infrared spectra were recorded using the Bruker Alpha II FT-IR spectrometer (Billerica, MA, USA). The ^1^H-NMR and ^13^C-NMR spectra were recorded on a Bruker AVANCE Neo 500 MHz and 125 MHz (Bruker, Switzerland, Model: Avance II 500 MHz) spectrometer, respectively, at room temperature. The chemical shifts (δ) are specified in parts per million (ppm), and the coupling constants (*J*) are designated in Hertz (Hz) values. TMS was used as an internal standard exhibiting δ 0 ppm, and deuterated CDCl_3_ was used as a solvent for NMR experiments. The abbreviation in NMR spectral data is s = singlet, d = doublet, dd = doublet of doublet, m = multiplet, and dt = doublet of triplet. Mass spectra were recorded using a MALDI SYNAPT XS HD Mass Spectrometer (Waters Corporation, Wilmslow, UK). The MAO isoforms were purchased from Sigma-Aldrich (Sigma-Aldrich Chemicals Private Limited, Bangalore, India). MAO inhibitory activity against both recombinant isoforms was accomplished by using the InvitogenTM (ThermoFisher Scientific India Private Limited, Mumbai, India). AmplexTM Red Hydrogen Peroxide/Peroxidase assay kit was procured from Thermo Fisher Scientific India Private Limited, Mumbai, India.

### 3.2. Conventional Method (CM) of Synthesis

Substituted benzaldehyde (0.974 mmol) and 4-morpholinoacetophenone (0.974 mmol) were dissolved in 40% NaOH and stirred at room temperature under a specified reaction time (mentioned in Table 1). After completion of the reaction (as monitored by TLC), the reaction mixture was poured into the ice-cold water, and the resulting product was collected by filtration. The product was purified by the recrystallization method using ethanol solvent and was then dried.

### 3.3. Microwave-Assisted Method of Synthesis

4-Morpholinoacetophenone (0.974 mmol) in 5% ethanolic NaOH (3 mL) and substituted benzaldehydes (0.974 mmol) were introduced to a microwave vial (10 mL) at room temperature. This vial was placed in a MW reactor (CEM Discover) and irradiated at 80 °C with a power of 50 Watts for 1–2 min. The progress of the reaction was monitored by TLC, and at the completion, the compounds were directly obtained as pure crystals. For the purification, the obtained crystals were collected by filtration, washed with cold ethanol, and dried.

### 3.4. Spectral Characterization Data

#### 3.4.1. (*E*)-3-(4-Bromophenyl)-1-(4-morpholinophenyl)prop-2-en-1-one (C1)

FTIR (ATR, *ν*_max_, cm^−1^): 2961.77, 2887.83, 2849.98 (C–H stretching), 1648.64 (C=O stretching), 1584.97 (C=C stretching), 1485.78 (C=C aromatic stretch), 1121.51 (C–O stretching), 1200.51 (C–N stretching), 676.10 (C–Br stretching). ^1^H NMR (500 MHz, CDCl_3_) δ ppm: 3.40 (4H, t, *J* = 5 Hz, morpholine CH_2_–N–CH_2_), 4.01 (4H, t, *J* = 5 Hz, morpholine –CH_2_–O–CH_2_–), 7.22 (2H, d, *J* = 10 Hz, H^1^ & H^3^), 7.50 (3H, m, H^19^, H^20^ & H^14^), 7.55 (2H, m, H^18^ & H^22^), 7.72 (1H, d, *J* = 15 Hz, H^15^), 8.04 (2H, d, *J* = 5 Hz, H^6^ & H^4^). ^13^C NMR (125 MHz, CDCl_3_) δ ppm: 188.0, 142.9, 133.9, 132.2, 130.6, 129.8, 129.7, 128.3, 122.2, 116.3, 66.8, 65.6, 51.1, 50.0. HRMS (TOF MS ESI^+^): *m/z* calculated for C_19_H_18_BrNO_2_ [M^+^]: 372.0594, [M^+^ + 2]: 374.0573; found: 372.0240, [M^+^ + 2]: 374.0195.

#### 3.4.2. (*E*)-3-(3,4-Dichlorophenyl)-1-(4-morpholinophenyl)prop-2-en-1-one (C2)

FTIR (ATR, *ν*_max_, cm^−1^): 2963.27, 2854.77 (C–H stretching), 1653.44 (C=O stretch), 1601.25 (C=C stretching), 1441.92, 1379.68 (C–C stretch, C–H bending), 1195.36 (C–O stretching), 1120.84 (C–N stretching), 812.68 (C–Cl stretching). ^1^H NMR (500 MHz, CDCl_3_) δ ppm: 3.35 (4H, t, *J* = 5 Hz, morpholine –N–), 3.88 (4H, t, *J* = 5 Hz, morpholine –O–), 6.93 (2H, d, *J* = 5 Hz, H^1^ & H^3^), 7.44 (1H, dd, *J* = 5 Hz, 10 Hz, H^18^), 7.48 (1H, d, *J* = 10 Hz, H^19^), 7.52 (1H, d, *J* = 15 Hz, H^14^), 7.67 (1H, d, *J* = 15 Hz, H^15^), 7.72 (1H, d, *J* = 5 Hz, H^22^), 8.00 (2H, dd, *J* = 5 Hz, H^6^, H^4^). ^13^C NMR (125 MHz, CDCl_3_) δ ppm: 187.3, 154.2, 140.4, 135.3, 133.9, 133.2, 130.8, 130.7, 129.5, 128.5, 127.4, 123.5, 113.5, 66.5, 47.5. HRMS (TOF MS ESI^+^): *m/z* calculated for C_19_H_17_Cl_2_NO_2_ [M^+^]: 362.0709, [M^+^ + 2]: 364.0680; observed [M^+^]: 362.0390, [M^+^ + 2]: 364.0343.

#### 3.4.3. (*E*)-3-(4-Fluorophenyl)-1-(4-morpholinophenyl)prop-2-en-1-one (C3)

FTIR (ATR, *ν*_max_, cm^−1^): 2976.30, 2850.93 (C–H stretching), 1651.43 (C=O stretching), 1611.36 (C=C stretching), 1385.03, 1320.57 (C–H bending), 1162.96 (C–O stretching), 1122.67 (C–N stretching), 656.58 (C–F stretching). ^1^H NMR (500 MHz, CDCl_3_) δ ppm: 3.32 (4H, t, *J* = 4 Hz, morpholine –N–), 3.85 (4H, t, *J* = 4 Hz, morpholine –O–), 6.90 (2H, d, *J* = 10 Hz, H^1^ & H^3^), 7.08 (2H, t, *J* = 10 Hz, H^21^ & H^19^), 7.47 (1H, d, *J* = 15 Hz, H^14^), 7.61 (2H, m, H^22^ & H^18^), 7.74 (1H, d, *J* = 10 Hz, H^15^), 7.99 (2H, d, *J* = 5Hz, H^6^ & H^4^). ^13^C NMR (125 MHz, CDCl_3_) δ ppm: 188.0, 164.9, 163.4 (*J*_C__–F_ = 187.5), 154.3, 142.4, 131.6, 131.6, 130.7, 130.4, 130.2, 130.1, 128.8, 121.7, 116.1, 113.4, 66.6, 47.5. HRMS (TOF MS ESI^+^): *m/z* calculated for C_19_H_18_FNO_2_ [M^+^]: 312.1394; observed [M^+^]: 312.1087.

#### 3.4.4. (*E*)-3-(3,4-Dimethoxyphenyl)-1-(4-morpholinophenyl)prop-2-en-1-one (C4)

FTIR (ATR, *ν*_max_, cm^−1^): 2970.50, 2836.02 (C–H stretching), 1643.50 (C=O stretching), 1569.48 (C=C stretching), 1431.77, 1375.75 (C–H bending), 1271.32, 1230.23, 1144.90, 1026.12, 932.47, 863.29, 813.72, 651.98 (C–C, C–N, C–O). ^1^H NMR (500 MHz, CDCl_3_) δ ppm: 3.33 (4H, t, *J* = 5 Hz, morpholine C–N–C), 3.87 (4H, t, *J* = 5 Hz, morpholine –C–O–C–), 3.93 (3H, s, OCH_3_, H^25^ ), 3.95 (3 H, s, OCH_3_, H^26^ ), 6.89 (1H, d, *J* = 10 Hz, H^18^), 6.92 (2H, d, *J* = 10 Hz, H^1^ & H^3^), 7.16 (1H, d, *J* = 1.5 Hz, H^19^), 7.23 (1H, dd, *J* = 5 Hz, 10 Hz, H^22^), 7.42 (1H, d, *J* = 15 Hz, H^14^), 7.75 (1H, d, *J* = 15 Hz, H^15^), 8.01 (2H, d, *J* = 5 Hz, H^6^ & H^4^). ^13^C NMR (125 MHz, CDCl_3_) δ ppm: 188.2, 154.0, 151.1, 149.2, 143.5, 130.5, 129.1, 128.2, 122.8, 119.9, 113.4, 111.1, 110.1, 66.5, 56.0, 47.6. HRMS (TOF MS ESI^+^): *m/z* calculated for C_21_H_23_NO_4_ [M^+^]: 354.1694; observed [M^+^]: 354.1326.

#### 3.4.5. (*E*)-3-(4-Bromo-3-fluorophenyl)-1-(4-morpholinophenyl)prop-2-en-1-one (C5)

FTIR (ATR, *ν*_max_, cm^−1^): 2968.34, 2854.57 (C–H stretching), 1653.40 (C=O stretching), 1596.70 (C=C stretching), 1483.41, 1372.50 (C–H bending), 1119.95 (C–O stretching, C–N stretching), 662.91 (C–Br stretching). ^1^H NMR (500 MHz, CDCl_3_) δ ppm: 3.34 (4H, t, *J* = 5 Hz, morpholine –N), 3.86 (4H, t, *J* = 5 Hz, morpholine –O–), 6.91 (2H, d, *J* = 10 Hz, H^1^ & H^3^), 7.27 (1H, d, *J* = 10 Hz, H^22^), 7.38 (1H, dd, *J* = 2 Hz, 10 Hz, H^18^), 7.53 (1H, d, *J* = 15 Hz, H^14^), 7.57 (1H, m, H^19^), 7.67 (1H, d, *J* = 15 Hz, H^15^), 8.00 (2H, d, *J* = 5 Hz, H^4^ & H^6^). ^13^C NMR (125 MHz, CDCl_3_) δ ppm: 187.3, 160.3, 158.3 (*J*_C–F_ = 246.2 ppm), 154.3, 140.7, 140.6, 136.8, 136.7, 133.9, 130.7, 130.3, 130.3, 128.4, 125.2, 123.5, 115.3, 115.1, 113.4, 113.1, 110.8, 110.6, 66.5, 47.4. HRMS (TOF MS ESI^+^): *m/z* calculated for C_19_H_17_BrFNO_2_ [M^+^]: 390.0499, [M^+^ + 2]: 392.0479; observed [M^+^]: 390.0092, [M^+^ + 2]: 392.0105.

#### 3.4.6. (*E*)-3-(3,4-Difluorophenyl)-1-(4-morpholinophenyl)prop-2-en-1-one (C6)

FTIR (ATR, *ν*_max_, cm^−1^): 2917.02, 2850.23 (C–H stretching), 1651.59 (C=O stretching), 1592.41 (C=C stretching), 1478.21 (C–H bending), 1190.42 (C–O stretching), 1114.23 (C–F stretch). ^1^H NMR (500 MHz, CDCl_3_) δ ppm: 3.36 (4H, t, *J* = 5 Hz, morpholine –N), 3.90 (4H, t, *J* = 5 Hz, morpholine –O–), 6.99 (2H, d, *J* = 10 Hz, H^1^ & H^3^), 7.28 (1H, dd, *J* = 5 Hz, 10 Hz, H^22^), 7.39 (1H, dd, *J* = 5 Hz, 10 Hz, H^18^), 7.53 (1H, d, *J* = 15 Hz, H^14^), 7.60–7.57 (1H, m, H^19^), 7.68 (1H, d, *J* = 15 Hz, H^15^), 8.00 (2H, d, *J* = 10 Hz, H^6^ & H^4^). ^13^C NMR (125 MHz, CDCl_3_) δ ppm: 187.3, 158.3, 154.3, 140.7, 136.8, 133.9, 130.7, 128.4, 125.2, 123.5, 115.3, 113.4, 110.8, 66.5, 47.4. HRMS (TOF MS ESI^+^): *m/z* calculated for C_19_H_17_F_2_NO_2_ [M^+^]: 330.1300: observed [M^+^]: 330.0986.

#### 3.4.7. (*E*)-3-(4-Chlorophenyl)-1-(4-morpholinophenyl)prop-2-en-1-one (C7)

FTIR (ATR, *ν*_max_, cm^−1^): 2966.67, 2845.58 (C–H stretching), 1647.85 (C=O stretching), 1605.57, 1585.46 (C=C stretching, alkene), 1447.35 (C=C stretch, aromatic), 1198.21 (C–O stretching), 1119.51 (C–N stretching), 669.03 (C–Cl stretch). ^1^H NMR (500 MHz, CDCl_3_) δ ppm: 3.34 (4H, t, *J* = 5 Hz, morpholine –N–), 3.88 (4H, t, *J* = 5 Hz, morpholine –O–), 6.95 (2H, d, *J* = 10 Hz, H^1^ & H^3^), 7.49 (3H, t, *J* = 5 Hz, H^19^, H^21^, H^14^), 7.53 (2H, d, *J* = 5 Hz, H^18^, H^22^), 7.71 (1H, d, *J* = 15 Hz, H^15^), 8.00 (2H, d, *J* = 5 Hz, H^4^ & H^6^). ^13^C NMR (125 MHz, CDCl_3_) δ ppm: 187.8, 153.8, 142.0, 134.1, 132.1, 130.6, 129.6, 129.1, 124.3, 122.4, 113.8, 66.4, 47.8. HRMS (TOF MS ESI^+^): *m/z* calculated for C_19_H_18_ClNO_2_ [M^+^]: 328.1099, [M^+^ + 2]: 330.1069; observed [M^+^]: 328.0777, [M^+^ + 2]: 330.0795.

#### 3.4.8. (*E*)-1-(4-Morpholinophenyl)-3-(m-tolyl)prop-2-en-1-one (C8)

FTIR (ATR, *ν*_max_, cm^−1^): 2963.65, 2848.79 (C–H stretching), 1650.72 (C=O stretching), 1595.34 (C=C stretching, alkene), 1439.67 (C=C stretch, aromatic), 1197.22 (C–O stretching), 1116.82 (C–N stretching), 664.44 (C–Cl stretch). ^1^H NMR (500 MHz, CDCl_3_) δ ppm: 2.39 (3H, CH_3_), 3.33 (4H, t, *J* = 5 Hz, morpholine –N–), 3.86 (4H, t, *J* = 5 Hz, morpholine –O–), 6.92 (2H, d, *J* = 5 Hz, H^1^ & H^3^), 7.21 (1H, d, *J* = 5 Hz, H^20^), 7.30 (1H, t, *J* = 5 Hz, H^19^), 7.44 (2H, d, *J* = 10 Hz, H^18^ & H^22^), 7.54 (1H, d, *J* = 15 Hz, H^14^), 7.76 (1H, d, *J* = 15 Hz, H^15^), 8.01 (2H, d, *J* = 5 Hz, H^4^ & H^6^). ^13^C NMR (125 MHz, CDCl_3_) δ ppm: 188.2, 154.1, 143.5, 138.5, 135.2, 131.0, 130.6, 130.3, 128.9, 128.9, 128.7, 125.5, 121.7, 113.4, 113.3, 66.6, 47.5, 21.3. HRMS (TOF MS ESI^+^): *m/z* calculated for C_20_H_21_NO_2_ [M^+^]: 308.1645; observed [M^+^]: 308.1355.

#### 3.4.9. (*E*)-1-(4-Morpholinophenyl)-3-(p-tolyl)prop-2-en-1-one (C9)

FTIR (ATR, *ν*_max_, cm^−1^): 2821.7 (C–H stretching), 1687.7 (C=O stretching), 1652.7 (C=C alkene), 1548.7 (C=C aromatic stretch). HRMS (TOF MS ESI^+^): *m/z* calculated for C_20_H_21_NO_2_ [M^+^]: 308.1645; observed [M^+^]: 308.1355.

#### 3.4.10. (*E*)-3-(3-Fluorophenyl)-1-(4-morpholinophenyl)prop-2-en-1-one (C10)

FTIR (ATR, *ν*_max_, cm^−1^): 1687.58 (C=O stretching), 1652.40 (C=C stretching, alkene), 1524.6 (C–F stretch), 1241.84 (C–N stretching), 1119.49 (C–O stretching). ^1^H NMR (500 MHz, CDCl_3_) δ ppm: 3.34 (4H, t, 5 Hz, morpholine –N–), 3.87 (4H, t, *J* = 5 Hz, morpholine –O–), 6.93 (2H, d, *J* = 5 Hz, H^1^ & H^3^), 7.09 (1H, m, H^22^), 7.36 (3H, m, H^18^, H^19^, & H^20^), 7.54 (1H, d, *J* = 15 Hz, H^14^), 7.74 (1H, d, *J* = 15 Hz, H^15^), 8.01 (2H, d, *J* = 5 Hz, H^6^ & H^4^). ^13^C NMR (125 MHz, CDCl_3_) δ ppm: 187.7, 154.2, 141.8, 137.6, 130.7, 130.4, 130.3, 128.6, 124.4, 123.1, 117.0, 116.8, 114.3, 114.2, 113.4, 66.5, 47.5. HRMS (TOF MS ESI^+^): *m/z* calculated for C_19_H_18_FNO_2_ [M^+^]: 312.1394; observed [M^+^]: 312.1149.

#### 3.4.11. (*E*)-3-(2-Methoxyphenyl)-1-(4-morpholinophenyl)prop-2-en-1-one (C11)

FTIR (ATR, *ν*_max_, cm^−1^): 2965.69, 2847.60 (C–H stretching), 1648.19 (C=O stretching), 1597.72 (C=C stretching, alkene), 1444.37 (C=C stretch, aromatic), 1187.53 (C–O stretching), 1115.77 (C–N stretching). ^1^H NMR (500 MHz, CDCl_3_) δ ppm: 3.32 (4H, t, *J* = 5 Hz, morpholine –N–), 3.86 (4H, t, *J* = 5 Hz, morpholine –O–), 3.91 (3H, s, OCH_3_), 6.91 (3H, m, H^1^, H^3^ & H^19^), 6.98 (1H, t, *J* = 10 Hz, H^21^), 7.36 (1H, dt, H^14^), 7.63 (2H, m, H^18^ & H^20^), 8.01 (2H, d, *J* = 5 Hz, H^4^ & H^6^), 8.09 (1H, d, *J* = 15 Hz, H^15^). ^13^C NMR (125 MHz, CDCl_3_) δ ppm: 188.8, 158.7, 154.0, 138.8, 131.3, 130.6, 129.2, 129.0, 124.3, 122.8, 120.7, 113.4, 111.2, 66.6, 55.5, 47.6. HRMS (TOF MS ESI^+^): *m/z* calculated for C_20_H_21_NO_3_ [M^+^]: 324.1589; observed [M^+^]: 324.1303.

#### 3.4.12. (*E*)-3-(2-Chlorophenyl)-1-(4-morpholinophenyl)prop-2-en-1-one (C12)

FTIR (ATR, *ν*_max_, cm^−1^): 2975.69, 2857.60 (C–H stretching), 1647.80 (C=O stretching), 1595.12 (C=C aromatic stretching), 1229.20 (C–O stretching), 1117.58 (C–N stretching).^1^H NMR (500 MHz, CDCl_3_) δ ppm: 3.34 (4H, t, *J* = 5 Hz, morpholine –N–), 3.87 (4H, t, *J* = 5 Hz, morpholine –O–), 6.91 (2H, d, *J* = 5 Hz, H^1^ & H^3^), 7.31 (2H, m, H^18^ & H^20^), 7.43 (1H, m, H^19^), 7.74 (1H, d, *J* = 15 Hz, H^14^), 7.74 (1H, m, H^21^), 8.00 (2H, d, *J* = 5 Hz, H^4^ & H^6^), 8.14 (1H, d, *J* = 15 Hz, H^15^). ^13^C NMR (125 MHz, CDCl_3_) δ ppm: 188.0, 154.2, 139.1, 135.3, 133.7, 130.7, 130.7, 130.2, 128.5, 127.7, 126.9, 124.9, 113.4, 66.5, 47.4. HRMS (TOF MS ESI^+^): *m/z* calculated for C_19_H_18_ClNO_2_ [M^+^]: 328.1099, [M^+^ + 2]: 330.1069; observed [M^+^]: 328.0641, [M^+^ + 2]: 330.0795.

#### 3.4.13. (*E*)-3-(4-Ethylphenyl)-1-(4-morpholinophenyl)prop-2-en-1-one (C13)

FTIR (ATR, *ν*_max_, cm^−1^): 2970.7, 2850.3 (C–H stretching), 1650.34 (C=O stretching), 1597.54 (C=C aromatic stretching), 1121.4 (C–N stretching). ^1^H NMR (500 MHz, CDCl_3_) δ ppm: 1.25 (3H, t, *J* = 10 Hz, CH_3_), 2.68 (2H, q, CH_2_), 3.33 (4H, t, *J* = 5 Hz, morpholine –N–), 3.87 (4H, t, *J* = 5 Hz, morpholine –O–), 6.92 (2H, d, *J* = 5 Hz, H^1^ & H^3^), 7.24 (2H, d, *J* = 10 Hz, H^19^ & H^21^), 7.52 (1H, d, *J* = 15 Hz, H^14^), 7.57 (2H, d, *J* = 5 Hz, H^18^ & H^22^), 7.78 (1H, d, *J* = 15 Hz, H^15^), 8.01 (2H, d, *J* = 5 Hz, H^4^ & H^6^). ^13^C NMR (125 MHz, CDCl_3_) δ ppm: 188.3, 154.0, 146.9, 143.4, 132.7, 130.6, 129.1, 128.4, 128.4, 121.0, 113.5, 66.5, 47.6, 28.8, 15.3. HRMS (TOF MS ESI^+^): *m/z* calculated for C_21_H_23_NO_2_ [M^+^]: 322.1802; observed [M^+^]: 322.1530.

#### 3.4.14. (*E*)-3-(2,4-Difluorophenyl)-1-(4-morpholinophenyl)prop-2-en-1-one (C14)

FTIR (ATR, *ν*_max_, cm^−1^): 2962.60, 2842.67 (C–H stretching), 1650.89 (C=O stretching), 1597.56 (C=C stretching, alkene), 1435.32 (C=C stretch, aromatic), 1191.28 (C–O stretching), 1118.57 (C–N stretching). ^1^H NMR (500 MHz, CDCl_3_) δ ppm: 3.34 (4H, t, *J* = 5 Hz, morpholine –N–), 3.87 (4H, t, *J* = 5 Hz, morpholine –O–), 6.89 (1H, m, H^21^), 6.92 (2H, d, *J* = 5 Hz, H^1^ & H^3^), 6.93 (1H, d, *J* = 5 Hz, H^18^), 7.61 (1H, m, H^14^ & H^19^), 7.81 (1H, d, *J* = 15 Hz, H^15^), 8.00 (2H, d, *J* = 5 Hz, H^4^ & H^6^). ^13^C NMR (125 MHz, CDCl_3_) δ ppm: 187.9, 154.2, 135.0, 130.7, 128.5, 113.4, 66.5, 47.4. HRMS (TOF MS ESI^+^): *m/z* calculated for C_19_H_17_F_2_NO_2_ [M^+^]: 330.1300; observed [M^+^]: 330.1050.

#### 3.4.15. (*E*)-1-(4-Morpholinophenyl)-3-(o-tolyl)prop-2-en-1-one (C15)

FTIR (ATR, *ν*_max_, cm^−1^): 2862.50, 2742.60 (C–H stretching), 1654.85 (C=O stretching), 1598.49 (C=C stretching, alkene), 1438.60 (C=C stretch, aromatic), 1115.20 (C–O stretching), 1041.69 (C–N stretching). ^1^H NMR (500 MHz, CDCl_3_) δ ppm: 2.48 (3H, CH_3_), 3.33 (4H, t, *J* = 5 Hz, morpholine –N–), 3.86 (4H, t, *J* = 5 Hz, morpholine –O–), 6.91 (2H, d, *J* = 5 Hz, H^1^ & H^3^), 7.22(2H, t, *J* = 5 Hz, H^20^ & H^21^), 7.29 (1H, dt, *J* = 5, 10 Hz, H^19^), 7.47 (1H, d, *J* = 15 Hz, H^14^), 7.68 (1H, d, *J* = 5 Hz, H^18^), 8.01 (2H, d, *J* = 5 Hz, H^4^ & H^6^), 8.08 (1H, d, *J* = 15 Hz, H^15^). ^13^C NMR (125 MHz, CDCl_3_) δ ppm: 188.1, 154.2, 141.0, 138.1, 134.3, 130.8, 130.6, 129.8, 128.9, 126.3, 126.2, 123.1, 113.4, 66.6, 47.5, 19.9. HRMS (TOF MS ESI^+^): *m/z* calculated for C_20_H_21_NO_2_ [M^+^]: 308.1645; observed [M^+^]: 308.1417.

### 3.5. MAO Inhibition Assay

The MAO inhibitory activity of the synthesized molecules was evaluated against both enzyme isoforms, hMAO-A and hMAO-B, using an Amplex Red^®^ assay kit. Standards clorgyline (MAO-A inhibitor) and pargyline (MAO-B inhibitor) were also included in the assay to assess inhibitory potential via the fluorimetric method, as described in the literature [47]. This study used 1.1 μg of hMAO-A enzyme and 7.5 μg of hMAO-B enzyme, each with their specific activities, in a 40 μL solution of both isoforms in each well of a 96-well plate (n = 3). The enzyme inhibition assay was performed at pH 8. A 10 μL solution of the synthesized compounds, at different concentrations (final concentrations of 0.2, 2, and 20 μM in each well), was prepared in 0.05 M sodium phosphate buffer. After adding 10 μL solution of the synthesized compounds, the enzyme solutions were incubated for 30 min at 37 °C in a CO_2_ incubator using a flat-bottom 96-well plate (Tarsons, Kolkata, India). A working solution (WS) of 10 mL containing 200 μL of tyramine (substrate, 1 mM), 200 μL of Amplex Red (200 μM), 100 μL of horseradish peroxidase (HRP, 1 U/mL), and 9.5 mL of buffer was prepared. After the incubation period, the WS was added to the enzyme-test compound mixture in the 96-well plates. Fluorescence was measured using BioTek Synergy H1 multimode microplate reader (Agilent Technologies India Private Limited, New Delhi, India) with excitation at 545 nm and emission at 590 nm to monitor reaction progress. Control tests utilized carrier solvents instead of test compounds, while blank tests (using the Amplex^®^ Red reagent without enzyme) were conducted to account for background interference in the readings, ensuring accurate measurement of H_2_O_2_ production during the enzymatic reaction.

### 3.6. Reversibility Inhibition Studies

Reversibility inhibition investigations were conducted using dilution methods as per the reported protocol [48], and all experiments were performed in triplicate. Using a flat-bottom 96-well plate (Tarsons), MAO enzymes were incubated with the test compounds C6 and C14 (concentrations 10× IC_50_ and 100× IC_50_) for 30 min at 37 °C. The samples were diluted 100 times with tyramine substrate after 30 min of incubation, resulting in final inhibitor concentrations of 0.1× IC_50_ and 1× IC_50_, respectively. Substrate and buffer solutions were used for the dilution. Clorgyline was employed as a positive control for MAO-A. After subtracting the background activities caused by the buffer and other media, the percentage of MAO activities recovered was computed with the control (without inhibitor) and plotted as a percentage of the enzyme activity and dilution factor of IC_50_.

### 3.7. Antioxidant Assay

The DPPH approach, which gauges the free radical scavenging activity, was used to evaluate the antioxidant properties of the compounds. The test was performed according to the literature method [50]. The stock solution of DPPH (0.5 mM) was prepared, and methanol was used to create each dilution. The different concentrations (10, 25, 50, 75, and 100 μM) of the test compounds (C1–C15) were prepared and compared with the standard ascorbic acid. The percentage antioxidant activity of the compounds was calculated using the following formula: % Antioxidant activity = [(A_absorbance_ − A_test_)/A_absorbance_] × 100.

### 3.8. Molecular Docking and ADME Pharmacokinetic Studies

The three-dimensional structure of the hMAO-A enzyme was retrieved from the RCSB protein data bank with PDB ID: 2Z5X [51] and prepared using the ‘Protein Preparation Wizard’ [53] of Glide module (Schrödinger Inc., New York, NY, USA). For the ligand preparation, the molecules were drawn in ChemDraw Professional 16.0, saved in .sdf format, imported into the Maestro, and subsequently used to prepare the ligands using the ‘LigPrep’ module [54]. A grid box was created on the prepared protein covering a volume of 20 Å around the native ligand using the ‘receptor-grid generation’ protocol to define a suitable binding site for the docking. The ligand docking module under Glide extra precision (XP) mode [55] was employed for molecular docking of the ligands. The binding orientation and molecular interactions were analyzed, accordingly. The ADME pharmacokinetics was determined using the SwissADME web tool http://www.swissadme.ch/ (accessed on 20 December 2024).

## 4. Conclusions

The morpholine-based chalcones were explored to check their potential for MAO-A enzyme inhibition which is mainly responsible for depression by causing catalytic deamination of neurotransmitters. A total of fifteen morpholine-based chalcones were synthesized and characterized, spectroscopically. Green chemistry involving the MW-assisted organic synthetic method was also conducted to compare the reaction time and product yields with the CM. The MW-assisted synthetic method demonstrated a better yield with a lower reaction time than the CM. The in vitro MAO-A inhibition study showed promising IC_50_ values of 7.91 ± 0.08 μM and 8.45 ± 0.19 μM for C14 and C6, respectively. Compounds C6 and C14 exhibited more than 60% reversibility during the MAO-A enzyme inhibition assay and showed more than 50% antioxidant potential in the DPPH assay. The in silico molecular docking study supported these results by exhibiting docking scores of −9.56 Kcal/mol (C14) and −9.45 Kcal/mol (C6) besides demonstrating π-π stacking interactions with the crucial amino acid residue Trp-397 similar to the standard ligand clorgyline. Hence, the safety and efficacy of these compounds in cellular models should be investigated before proceeding to in vivo evaluation.

## Figures and Tables

**Table 1:** Comparison of reaction time and yield (%) of conventional and microwave methods.

Compound	Conventional Method		Microwave Method *	
	Reaction Time (h)	Yield %	Reaction Time (min)	Yield (%)
C1	12	88	1	96
C2	10	79	1	88
C3	11	80	1.5	89
C4	18	84	2	90
C5	24	77	1	87
C6	12	75	1.2	85
C7	6	81	1	90
C8	8	85	2	91
C9	9	86	1	94
C10	10	82	2	90
C11	12	75	1.4	85
C12	24	88	2	95
C13	12	80	2	90
C14	13	84	1	91
C15	24	70	2	82

* Microwave method: temperature of 80 °C with power of 50 W.

**Table 2:** Physical characterization data of the synthesized chalcones (C1–C15).

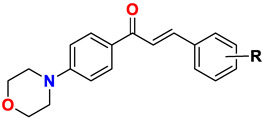						
Compound	R	Mol. Formula	Mol. Weight (g/mol)	Color	Melting Point	* R_f_
C1	4-Br	C_19_H_18_BrNO_2_	372.26	Yellow	189–190 °C	0.4
C2	3,4-diCl	C_19_H_17_Cl_2_NO_2_	362.25	Yellow	127–128 °C	0.44
C3	4-F	C_19_H_18_FNO_2_	311.35	Yellow	150–151 °C	0.36
C4	3,4-diOCH_3_	C_21_H_23_NO_4_	353.41	Yellow	159–160 °C	0.2
C5	3-F,4-Br	C_19_H_17_BrFNO_2_	390.25	Yellow	191–192 °C	0.4
C6	3,4-diF	C_19_H_17_F_2_NO_2_	329.34	Yellow	161–162 °C	0.4
C7	4-Cl	C_19_H_18_ClNO_2_	327.81	Yellow	188–189 °C	0.35
C8	3-CH_3_	C_20_H_21_NO_2_	307.39	Yellow	144–145 °C	0.5
C9	4-CH_3_	C_20_H_21_NO_2_	307.39	Yellow	186–187 °C	0.4
C10	3-F	C_19_H_18_FNO_2_	311.36	Yellow	124–125 °C	0.4
C11	2-OCH_3_	C_20_H_21_NO_3_	323.39	Yellow	144–145 °C	0.5
C12	2-Cl	C_19_H_18_ClNO_2_	327.80	Yellow	163–164 °C	0.4
C13	4-CH_2_CH_3_	C_21_H_23_NO_2_	321.41	Yellow	131–132 °C	0.4
C14	2,4-diF	C_19_H_17_F_2_NO_2_	329.34	Yellow	171–172 °C	0.4
C15	2-CH_3_	C_20_H_21_NO_2_	307.39	Light yellow	130–131 °C	0.5

* R_f_ = retardation factor; solvent system: *n*-hexane–ethyl acetate in 7:3 ratio. All compounds (C1–C15) are obtained in a solid state. Heteroatoms [Nitrogen: N = blue (dark) and Oxygen: O = Red] are colored according to the standard CPK rule on the structure.

**Table 3:** The percentage inhibition and IC_50_ values of the synthesized compounds against hMAO-A and hMAO-B.

Compound	Percentage Inhibition at 20 μM		* IC_50_ Values (μM ± SEM)	
	MAO-A	MAO-B	MAO-A	MAO-B
C1	13.64	6.59	---	---
C2	19.43	4.74	---	---
C3	37.74	7.11	---	---
C4	15.85	5.56	---	---
C5	36.81	6.54	---	---
C6	71.67	31.94	8.45 ± 0.19	---
C7	21.41	7.43	---	---
C8	3.11	6.32	---	---
C9	4.95	6.77	---	---
C10	33.93	12.37	---	---
C11	21.11	18.57	---	---
C12	67.76	23.85	9.35 ± 0.11	---
C13	7.25	17.54		---
C14	75.45	35.34	7.91 ± 0.08	---
C15	6.17	25.66		---
Clorgyline	94.98	---	4.39 ± 1.02 nM	nd
Pargyline	---	98.96	nd	0.079 ± 0.004 nM

“---“ Percentage inhibition less than 50% at a concentration of 20 μM; nd: not determined; * IC_50_ was calculated only for the compounds showing a % of inhibition greater than 50% at a concentration of 20 μM.

**Table 4:** DPPH free radical scavenging potential of compounds C1-C15.

Compound	Absorbance at 517 nm (% Antioxidant Activity)				
	10 µM	25 µM	50 µM	75 µM	100 µM
Control	0.048	0.079	0.055	0.058	0.058
Ascorbic acid	0.045(8.03)	0.064 (20.27)	0.033 (40.23)	0.031 (46.33)	0.024 (58.53)
C1	0.044 (9.24)	0.062 (21.80)	0.035 (35.78)	0.027 (53.13)	0.026 (54.82)
C2	0.045 (6.63)	0.059 (25.78)	0.035 (37.18)	0.029 (50.91)	0.027 (53.12)
C3	0.041(14.58)	0.049 (38.25)	0.033 (40.31)	0.027 (53.10)	0.025 (56.87)
C4	0.040 (16.37)	0.054 (32.47)	0.033 (41.07)	0.039 (33.69)	0.028 (52.62)
C5	0.039 (19.11)	0.061 (23.41)	0.034 (38.15)	0.029 (49.88)	0.029 (50.95)
C6	0.042 (12.25)	0.060 (24.46)	0.035 (36.03)	0.027 (53.39)	0.026 (55.16)
C7	0.042 (13.81)	0.067 (15.62)	0.038 (32.06)	0.028 (52.12)	0.027 (53.37)
C8	0.040 (16.56)	0.055 (31.47)	0.035 (36.01)	0.029 (49.24)	0.020 (66.42)
C9	0.044 (8.65)	0.061 (23.43)	0.035 (35.75)	0.035 (39.19)	0.032 (44.66)
C10	0.040 (17.09)	0.063 (21.05)	0.036 (34.56)	0.034 (42.27)	0.039 (32.62)
C11	0.042 (13.89)	0.067 (16.00)	0.033 (40.26)	0.042 (27.09)	0.037 (36.45)
C12	0.047 (3.03)	0.064 (19.54)	0.033 (40.88)	0.032 (44.56)	0.037 (35.95)
C13	0.047 (2.54)	0.067 (15.55)	0.032 (41.40)	0.045 (22.18)	0.019 (66.87)
C14	0.043 (10.51)	0.065 (18.21)	0.033 (41.12)	0.027 (53.12)	0.024 (59.51)
C15	0.045 (6.51)	0.055 (30.71)	0.033 (41.06)	0.039 (32.50)	0.041 (29.17)

**Table 5:** Docking score and ADME pharmacokinetics data of C1-C15 and Clorgyline.

Compound	Docking Score	Molecular Weight	H-Bond Acceptor	Log P	Log K_p_ (Skin Permeation) (cm/s)	GI Absorption	BBB Permeability	Bioavailability Score
C1	−7.48	372.26	2	3.39	−5.64	High	Yes	0.55
C2	−8.9	362.25	2	3.37	−5.17	High	Yes	0.55
C3	−9.24	311.35	3	3.17	−5.69	High	Yes	0.55
C4	−6.66	353.41	4	3.37	−6.06	High	Yes	0.55
C5	−8.98	390.25	3	3.37	−5.68	High	Yes	0.55
C6	−9.45	329.34	4	3.16	−5.72	High	Yes	0.55
C7	−7.99	327.8	2	3.31	−5.41	High	Yes	0.55
C8	−8.57	307.39	2	3.24	−5.48	High	Yes	0.55
C9	−9.09	307.39	2	3.17	−5.48	High	Yes	0.55
C10	−8.95	311.35	3	3.16	−5.69	High	Yes	0.55
C11	−8.98	323.39	3	3.2	−5.55	High	Yes	0.55
C12	−9.3	327.8	2	3.21	−5.28	High	Yes	0.55
C13	−7.61	321.41	2	3.4	−5.25	High	Yes	0.55
C14	−9.56	329.34	4	3.15	−5.72	High	Yes	0.55
C15	−8.49	307.39	2	3.13	−5.48	High	Yes	0.55
Clorgyline	−4.27	272.17	2	3.43	−4.99	High	Yes	0.55

## Data Availability

The original contributions presented in this study are included in the article/Supplementary Material. Further inquiries can be directed to the corresponding authors.

## Supplementary Materials

The following supporting information can be downloaded at: https://www.mdpi.com/article/10.3390/ph18030309/s1, Figures S1–S58: FTIR, ^1^H NMR, ^13^C NMR, and HRMS spectral images of the synthesized chalcones C1–C15.
