# Supplementary material for: Microwave-Assisted Synthesis of Morpholine-Based Chalcones as Reversible MAO-A Inhibitors in the Management of Mental Depression
Source: Pharmaceuticals (Basel). 2025 Feb 23;18(3):309. doi: 10.3390/ph18030309 (PMC11945657; doi:10.3390/ph18030309)
Supplement: Supplementary file 1 [file pharmaceuticals-18-00309-s001.zip › pharmaceuticals-3444840-supplementary.pdf]

Supplementary Materials

Spectral Images

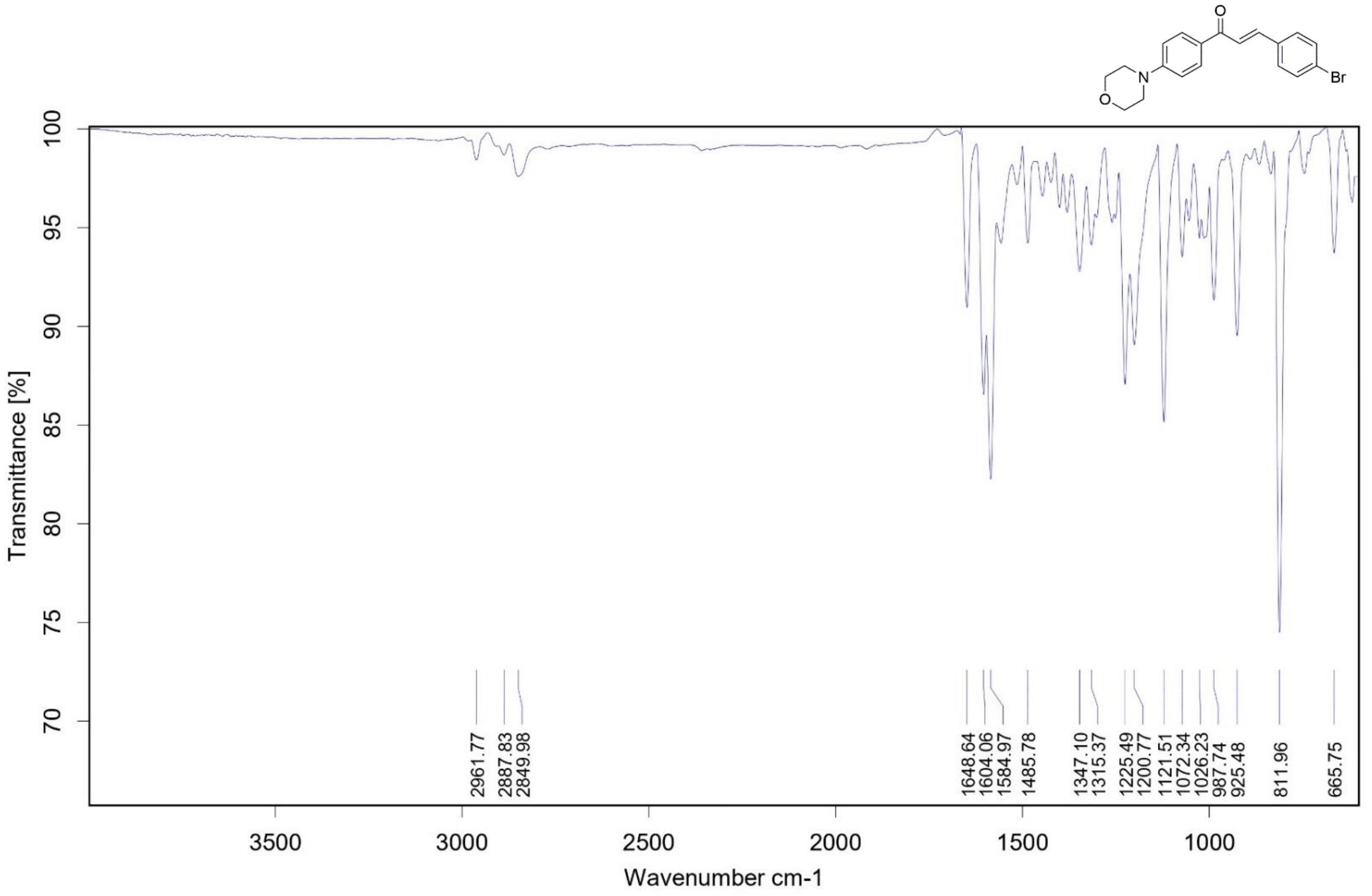

Figure S1. FT-IR of **C1**

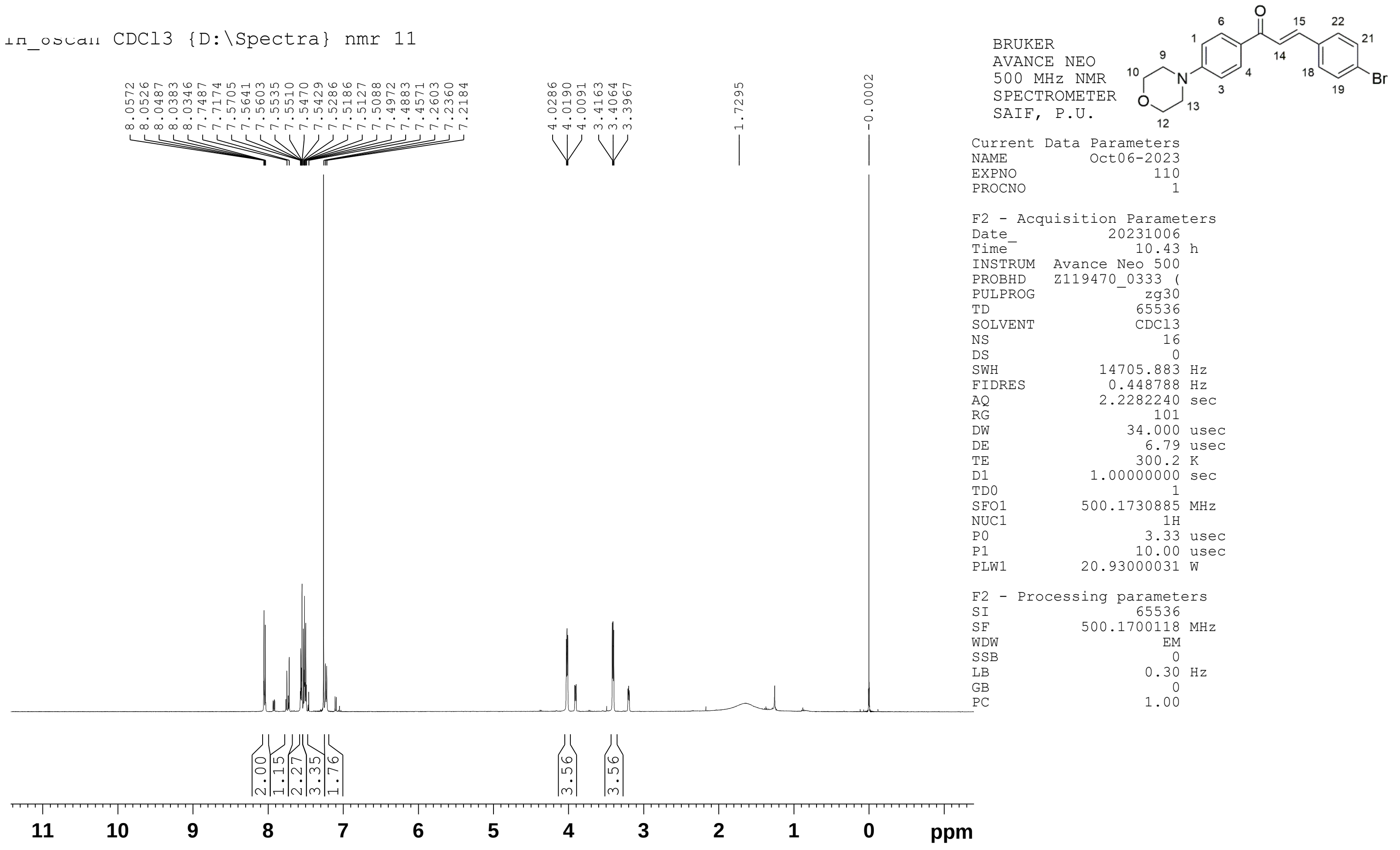

Figure S2. ^{1}H NMR of **C1**

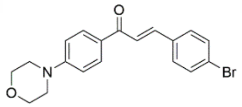

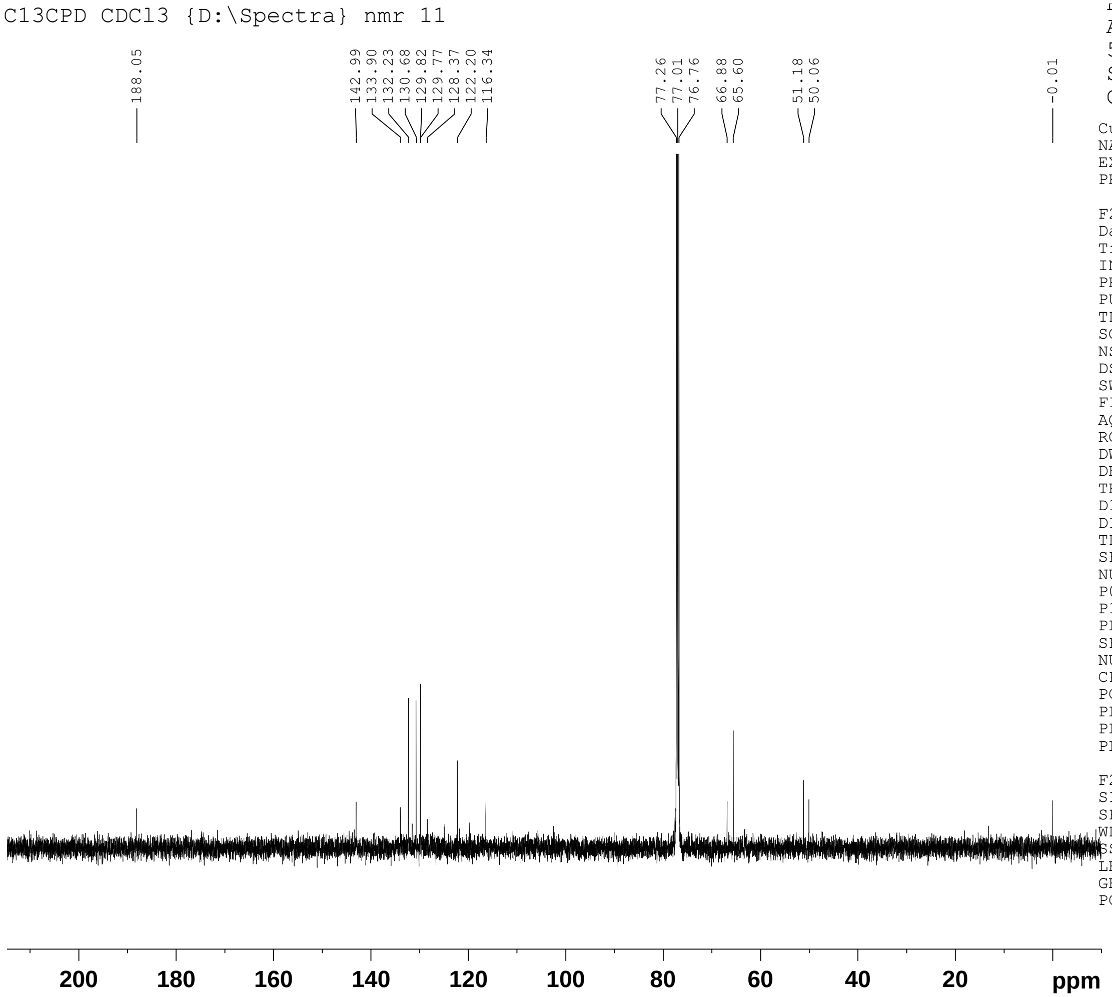

BRUKER
AVANCE NEO
500 MHz NMR SPECTROMETER
SAIF, PANJAB UNIVERSITY,
CHANDIGARH

Current Data Parameters
NAME Oct06-2023
EXPNO 112
PROCNO 1

F2 - Acquisition Parameters
Date_ 20231006
Time 22.58 h
INSTRUM Avance Neo 500
PROBHD Z119470_0333 (
PULPROG zgpg30
TD 65536
SOLVENT CDCl3
NS 2048
DS 4
SWH 37037.035 Hz
FIDRES 1.130281 Hz
AQ 0.8847360 sec
RG 101
DW 13.500 usec
DE 6.50 usec
TE 300.1 K
D1 2.00000000 sec
D11 0.03000000 sec
TD0 1
SFO1 125.7804233 MHz
NUC1 13C
P0 3.33 usec
P1 10.00 usec
PLW1 83.14099884 W
SFO2 500.1720007 MHz
NUC2 1H
CPDPRG[2 waltz65
PCPD2 80.00 usec
PLW2 20.93000031 W
PLW12 0.32703000 W
PLW13 0.16449000 W

F2 - Processing parameters
SI 32768
SF 125.7678465 MHz
WDW EM
SSB 0
LB 1.00 Hz
GB 0
PC 1.40

Figure S3. ^{13}C NMR of **C1**

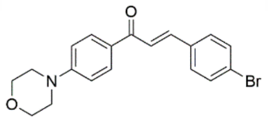

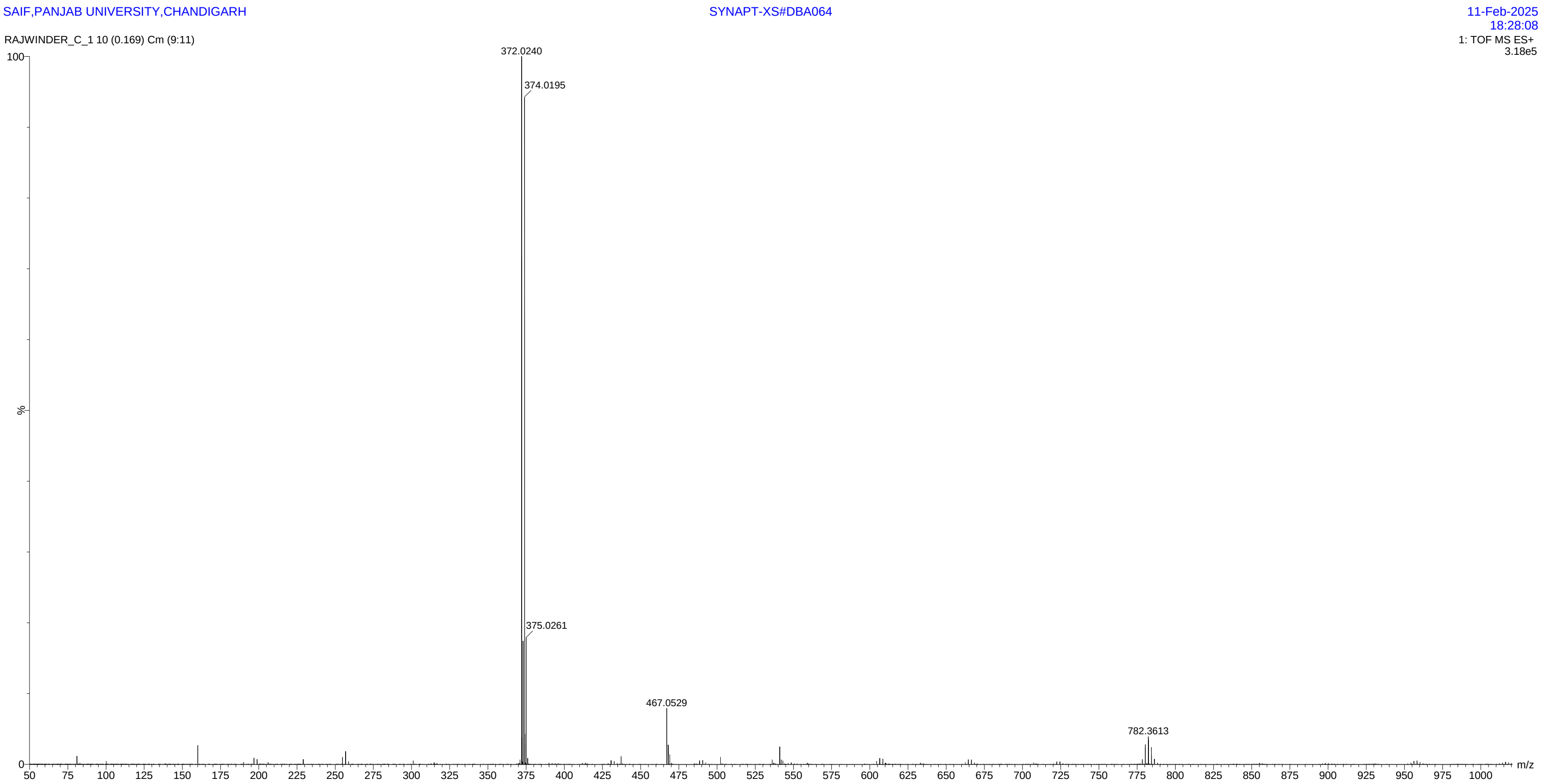

Figure S4. HRMS of **C1**

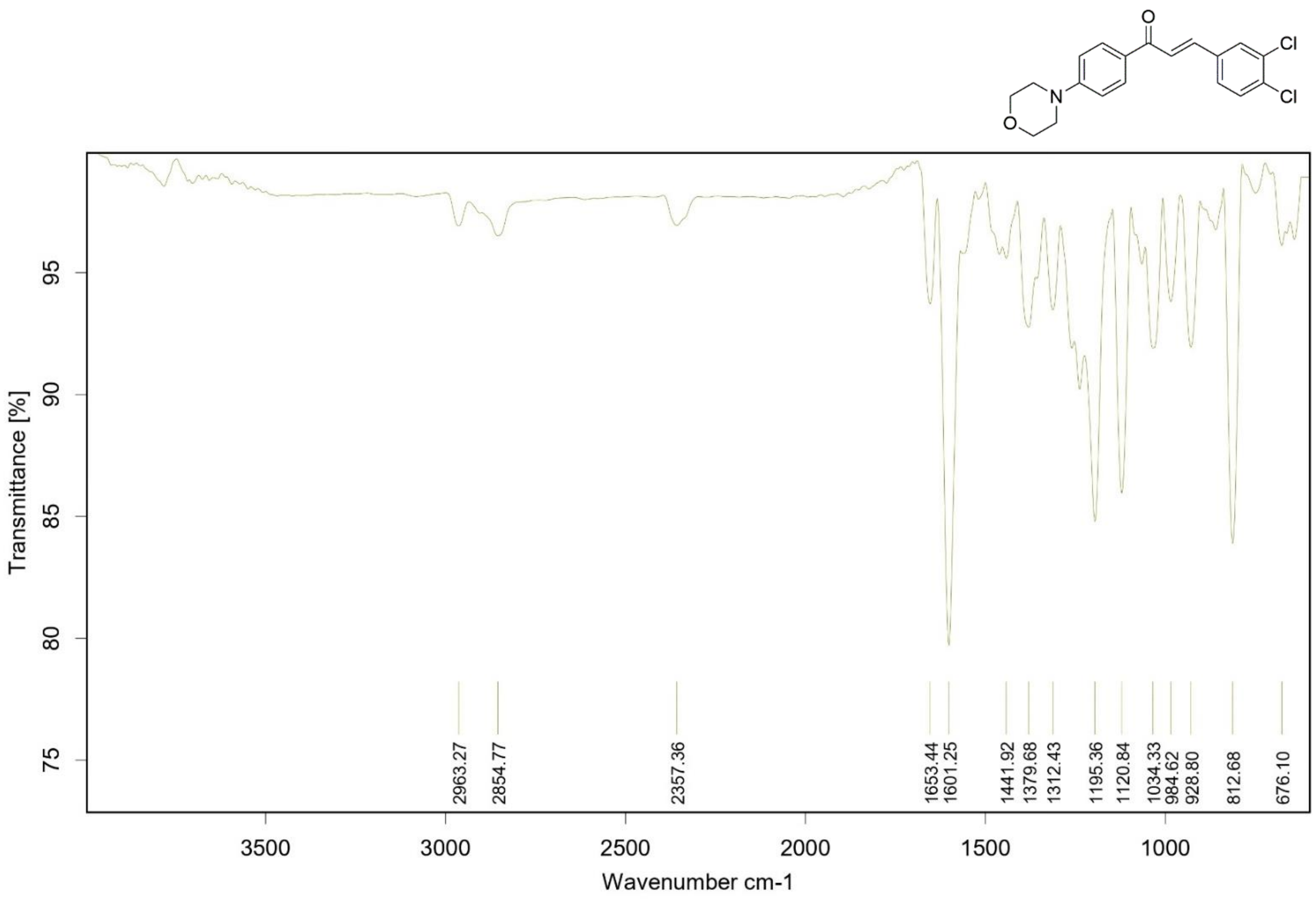

Figure S5. FT-IR of **C2**

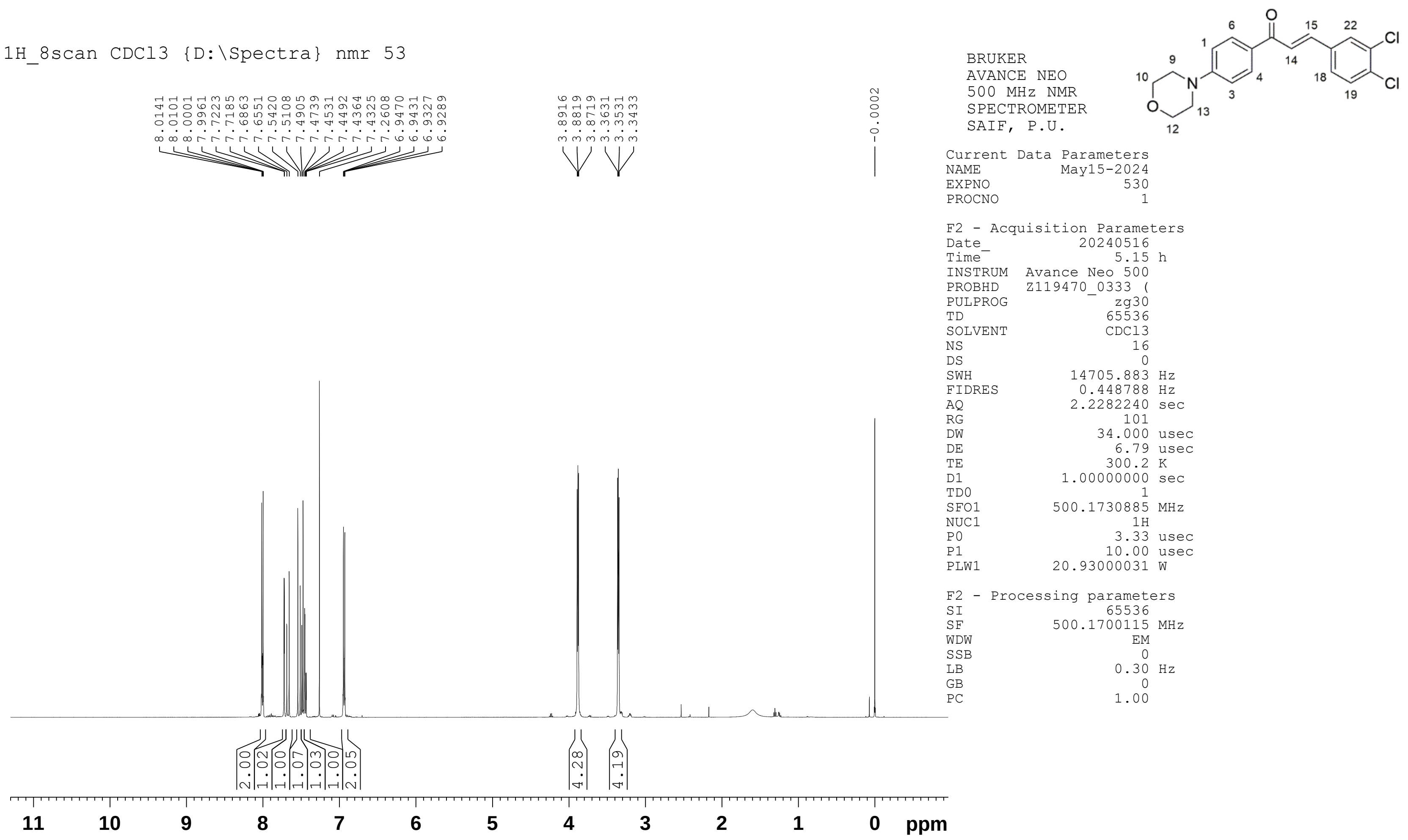

Figure S6. ^{1}H NMR of **C2**

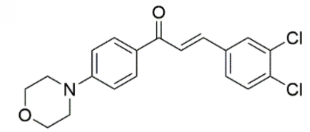

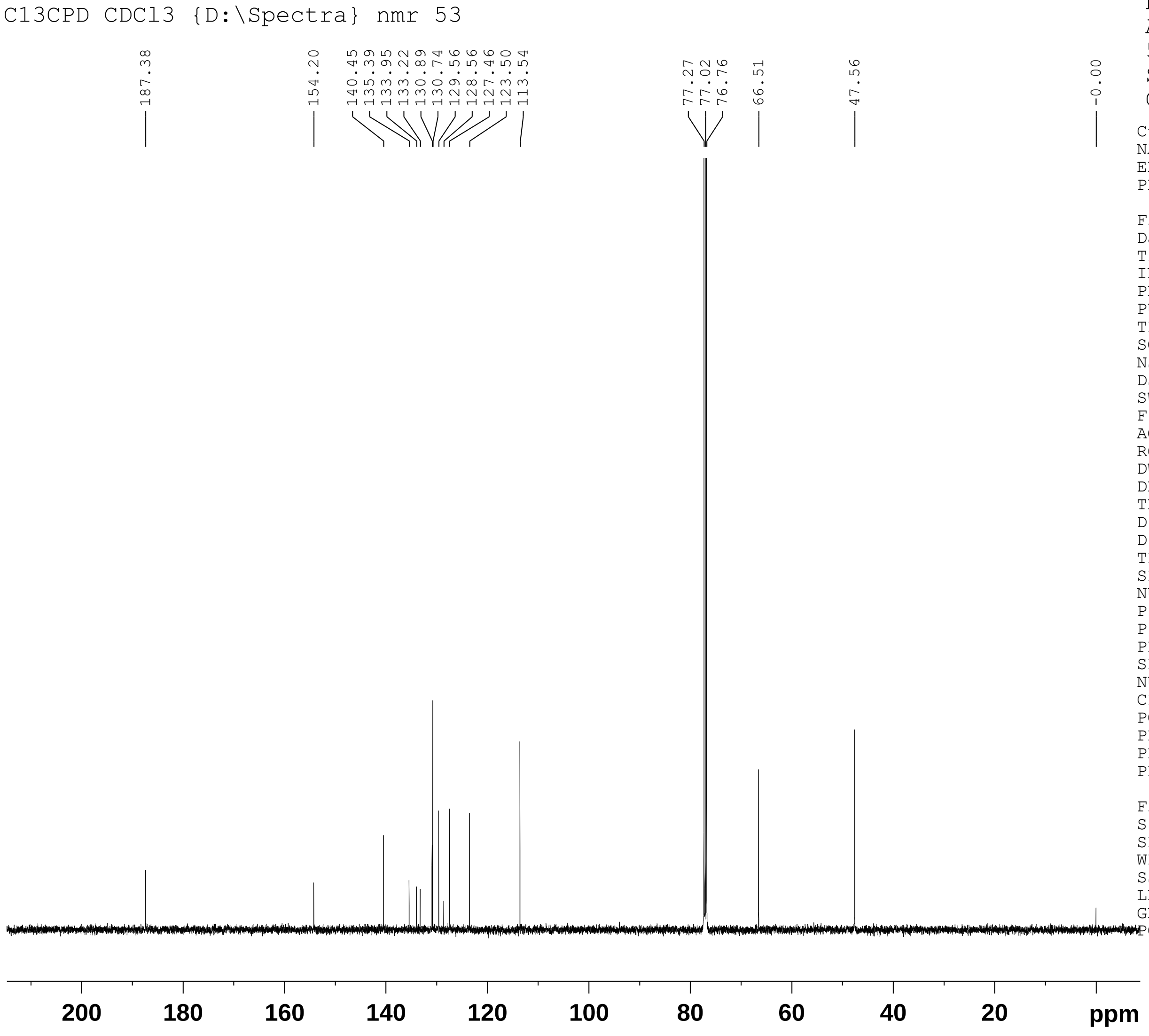

BRUKER
AVANCE NEO
500 MHz NMR SPECTROMETER
SAIF, PANJAB UNIVERSITY,
CHANDIGARH

```
Current Data Parameters
NAME          May15-2024
EXPNO                531
PROCNO                 1

F2 - Acquisition Parameters
Date_           20240516
Time                5.41 h
INSTRUM   Avance Neo 500
PROBHD   Z119470_0333 (
PULPROG          zgpg30
TD                 65536
SOLVENT            CDCl3
NS                   512
DS                     4
SWH            37037.035 Hz
FIDRES          1.130281 Hz
AQ             0.8847360 sec
RG                   101
DW                13.500 usec
DE                  6.50 usec
TE                 300.2 K
D1            2.00000000 sec
D11           0.03000000 sec
TD0                    1
SFO1         125.7804233 MHz
NUC1                 13C
P0                  3.33 usec
P1                 10.00 usec
PLW1         83.14099884 W
SFO2         500.1720007 MHz
NUC2                  1H
CPDPRG[2         waltz65
PCPD2              80.00 usec
PLW2         20.93000031 W
PLW12         0.32703000 W
PLW13         0.16449000 W

F2 - Processing parameters
SI                 32768
SF           125.7678465 MHz
WDW                   EM
SSB                    0
LB                  1.00 Hz
GB                     0
PC                  1.40
```

Figure S7. ^{13}C NMR of **C2**

Manuscript ID: **pharmaceuticals-3444840**

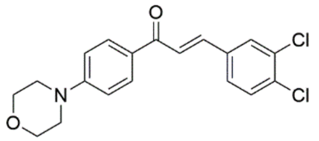

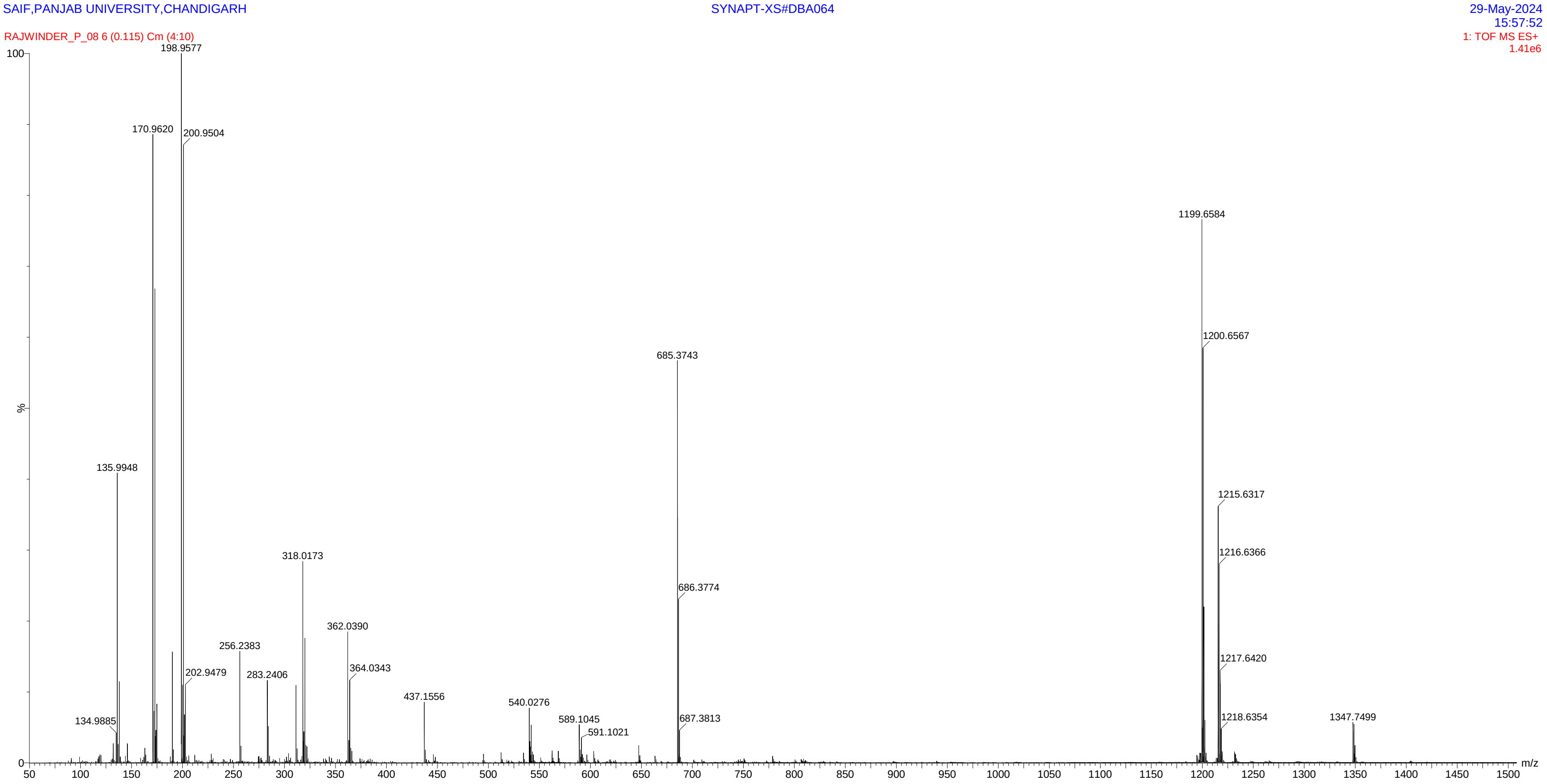

Figure S8. HRMS of **C2**

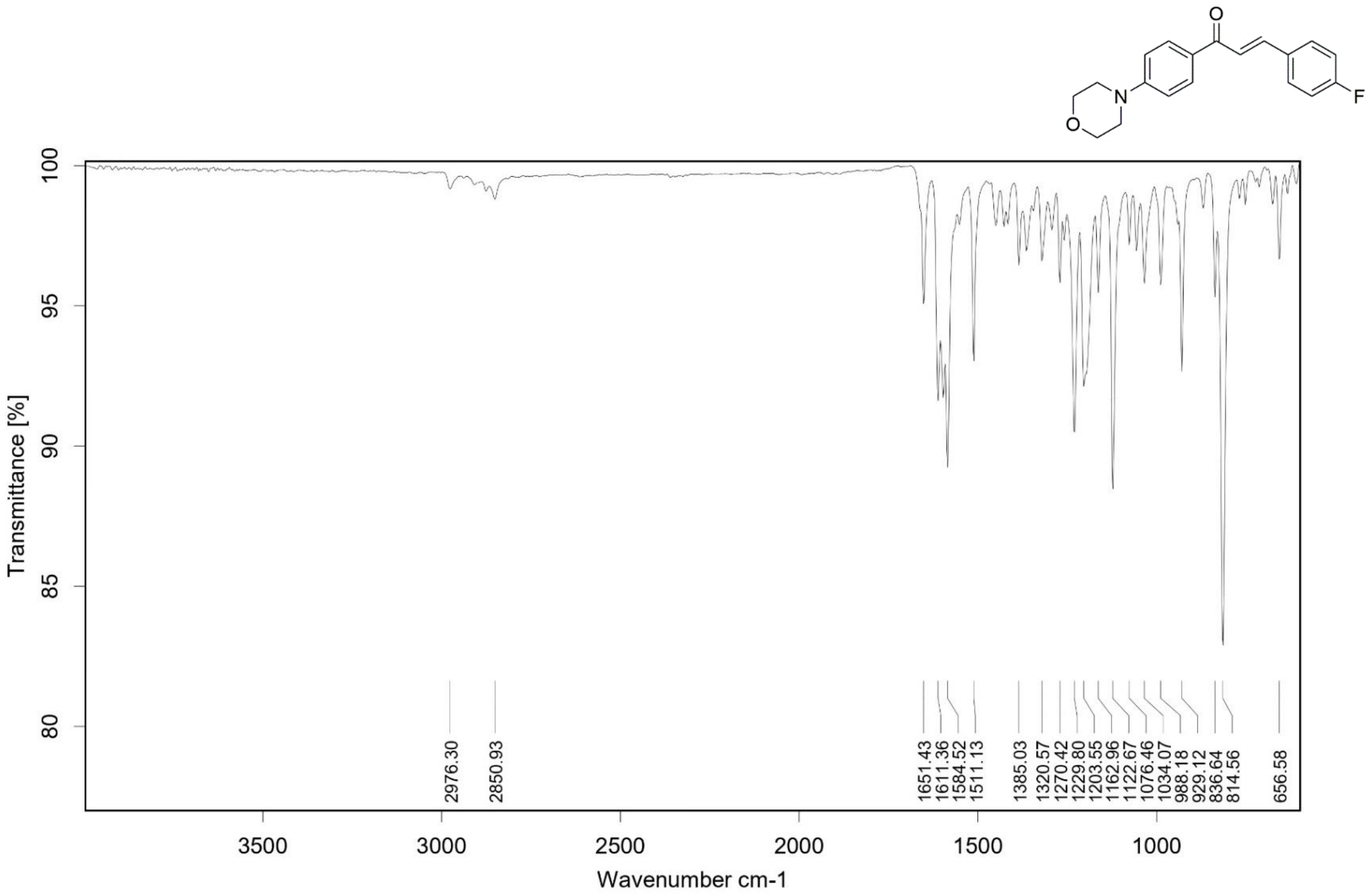

Figure S9. FT-IR of **C3**

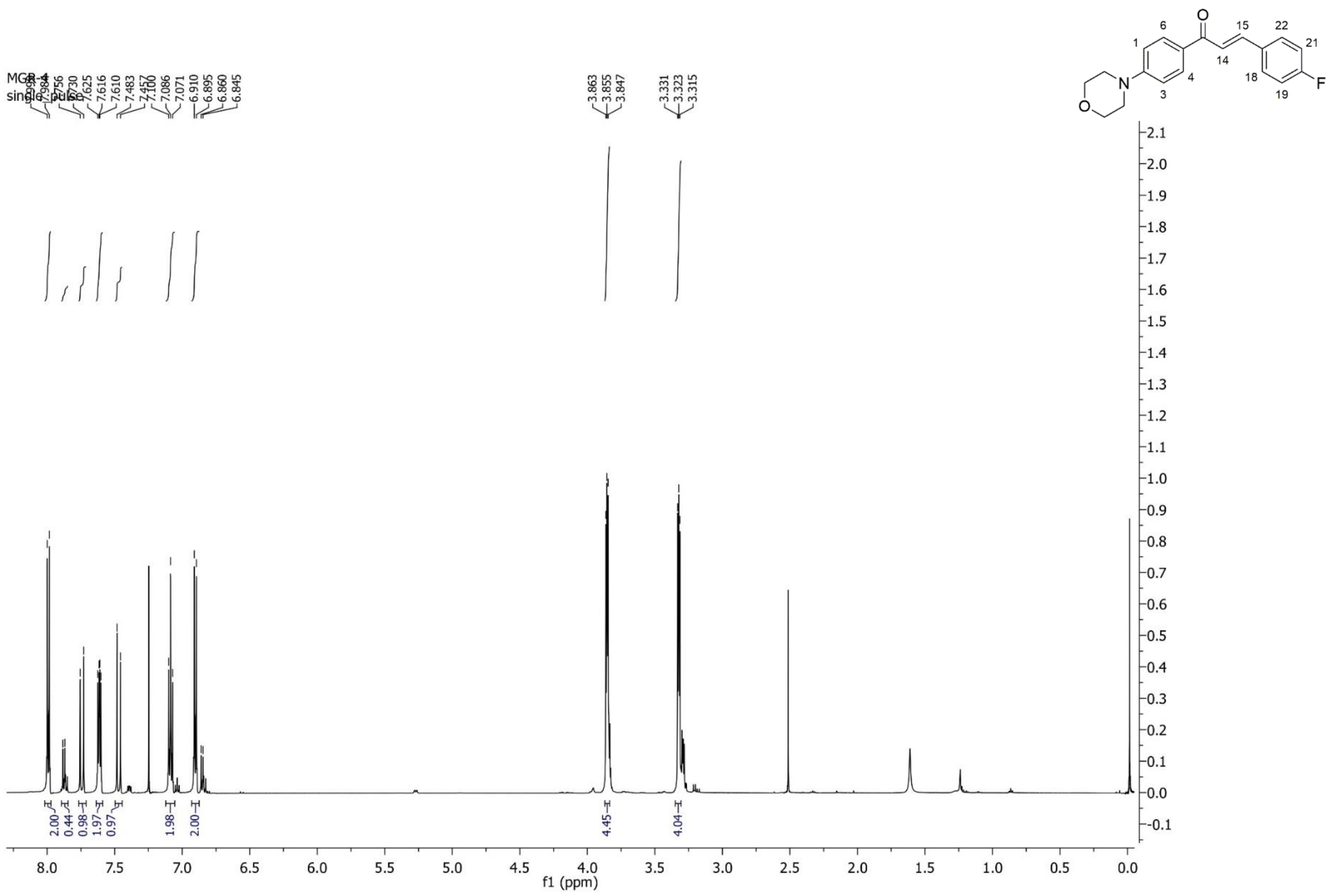

Figure S10. ^{1}H NMR of **C3**

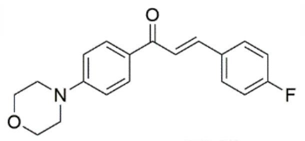

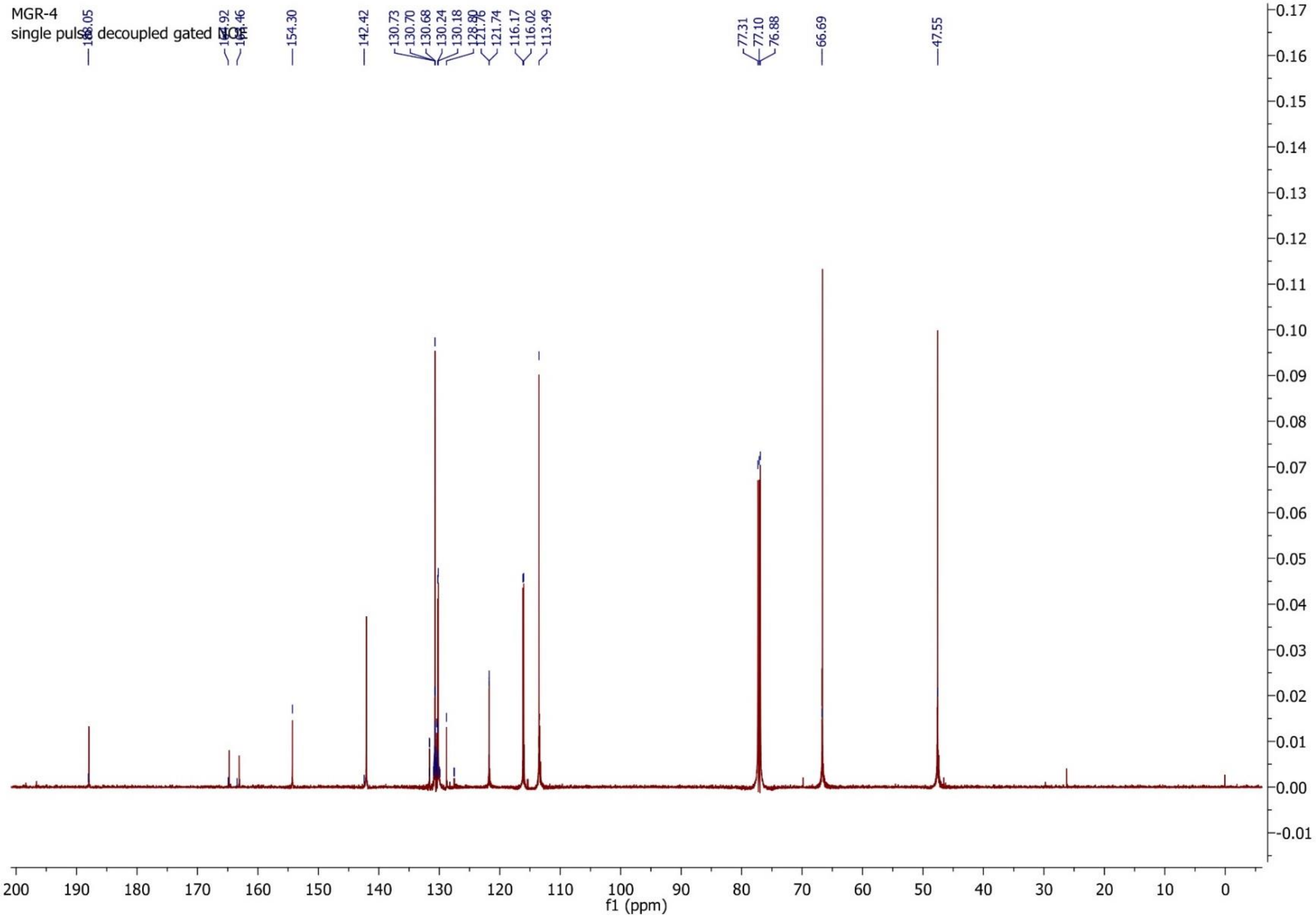

Figure S11. ^{13}C NMR of **C3**

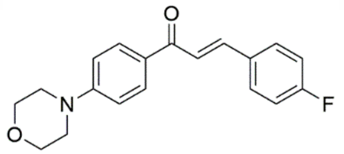

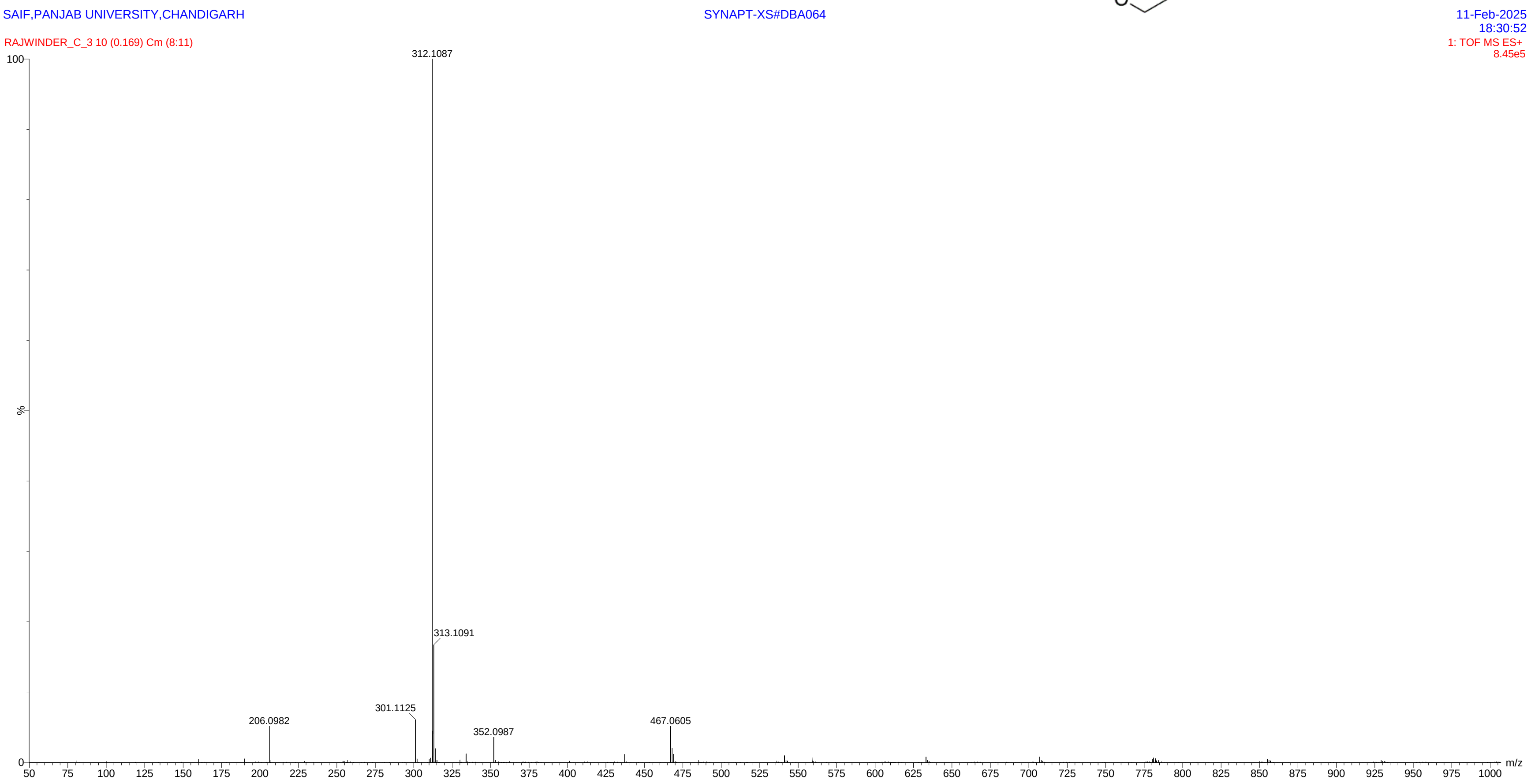

Figure S12. HRMS of **C3**

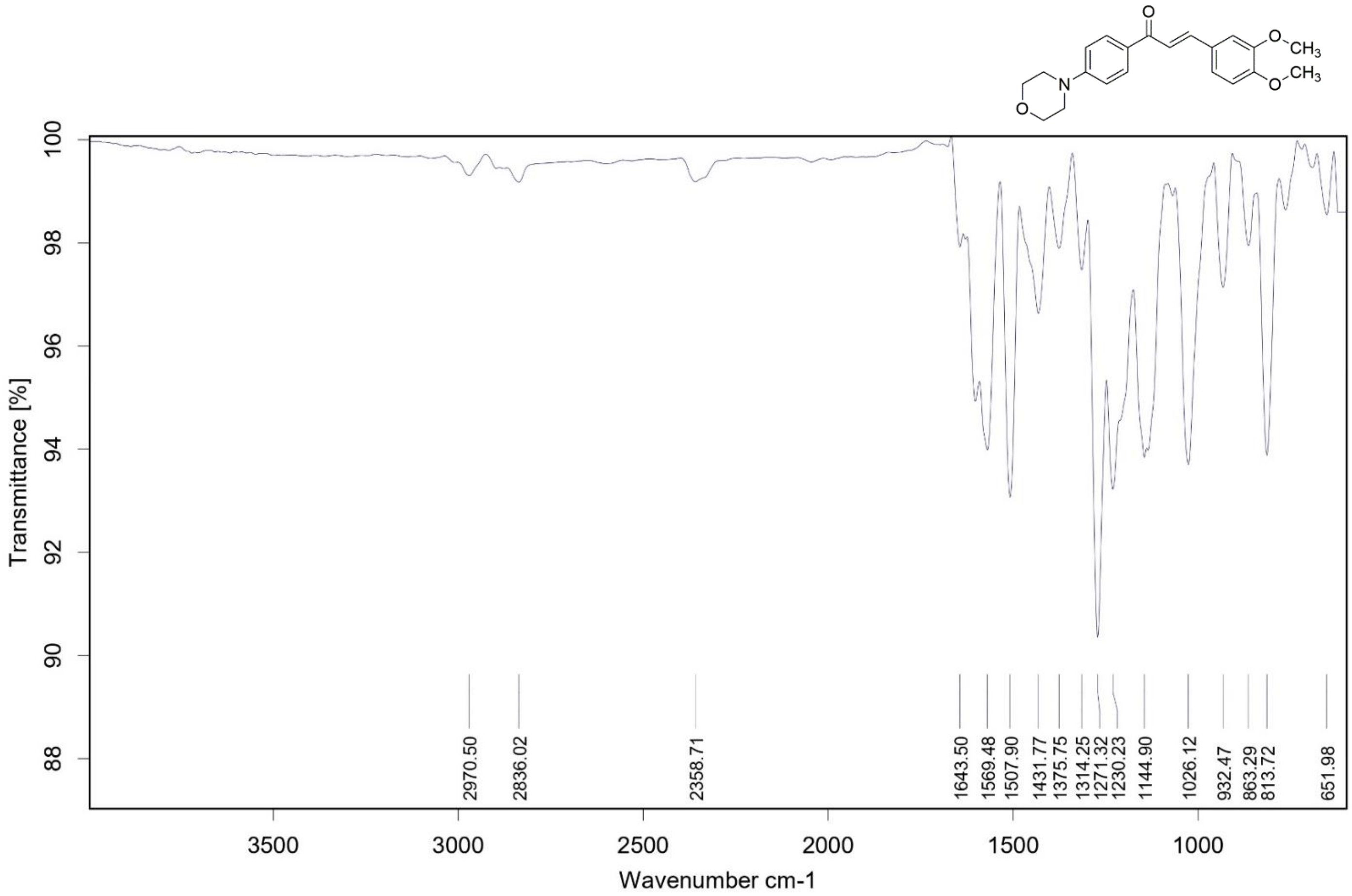

Figure S13. FT-IR of **C4**

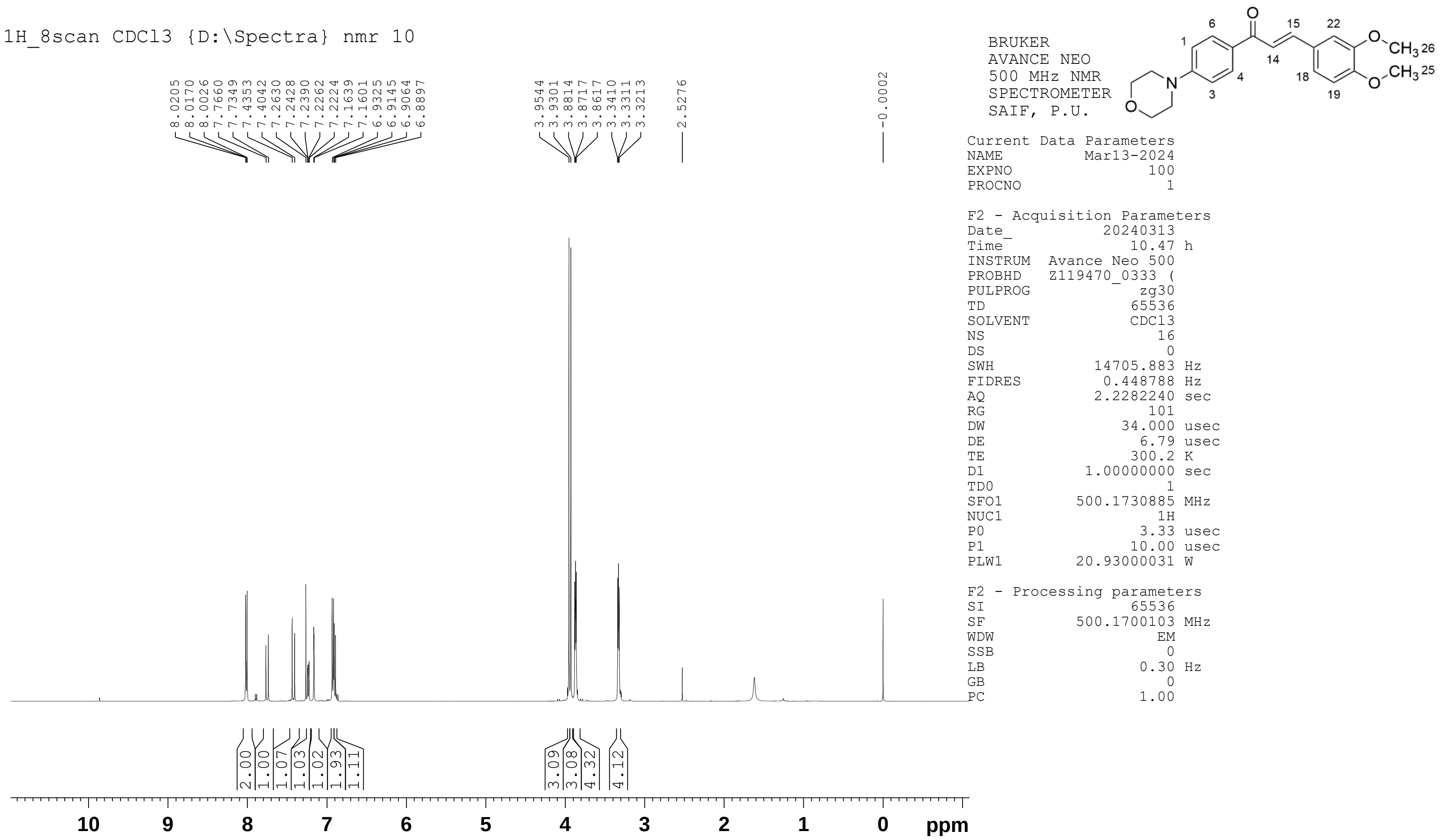

Figure S14. ^{1}H NMR of **C4**

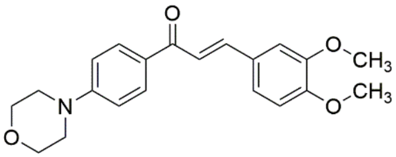

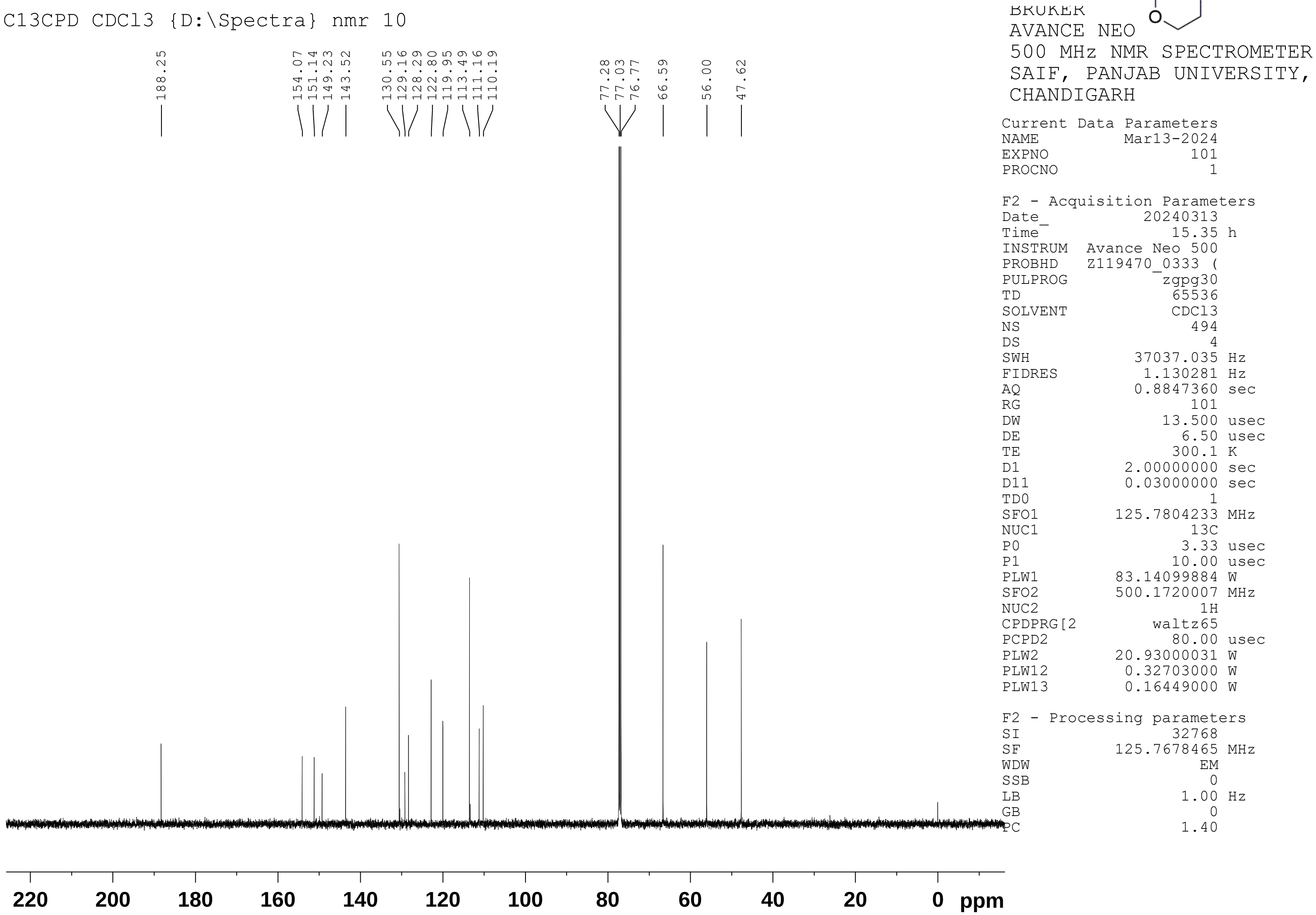

Figure S15. ^{13}C NMR of **C4**

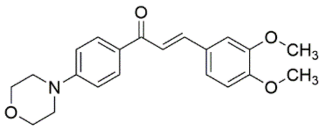

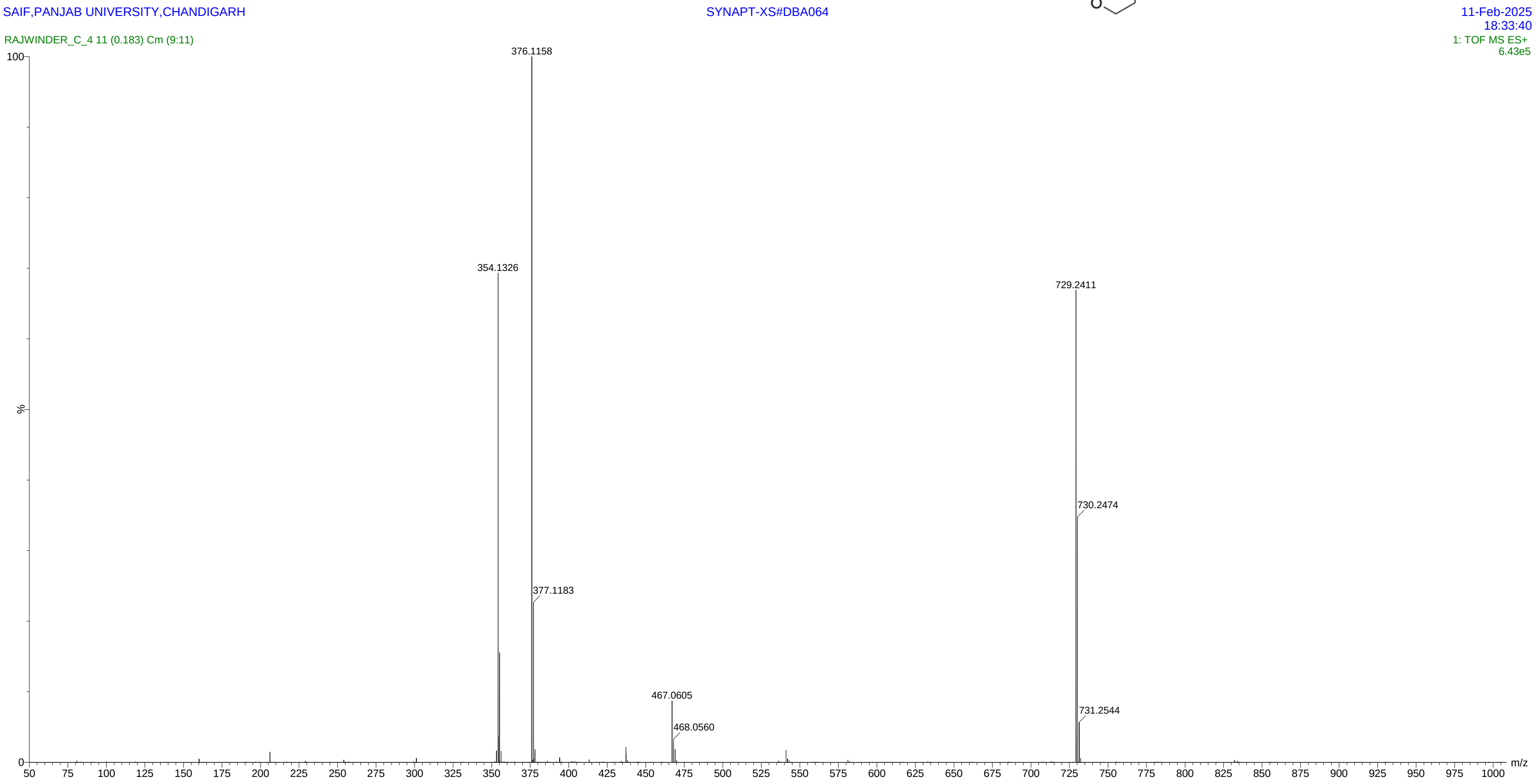

Figure S16. HRMS of **C4**

Manuscript ID: **pharmaceuticals-3444840**

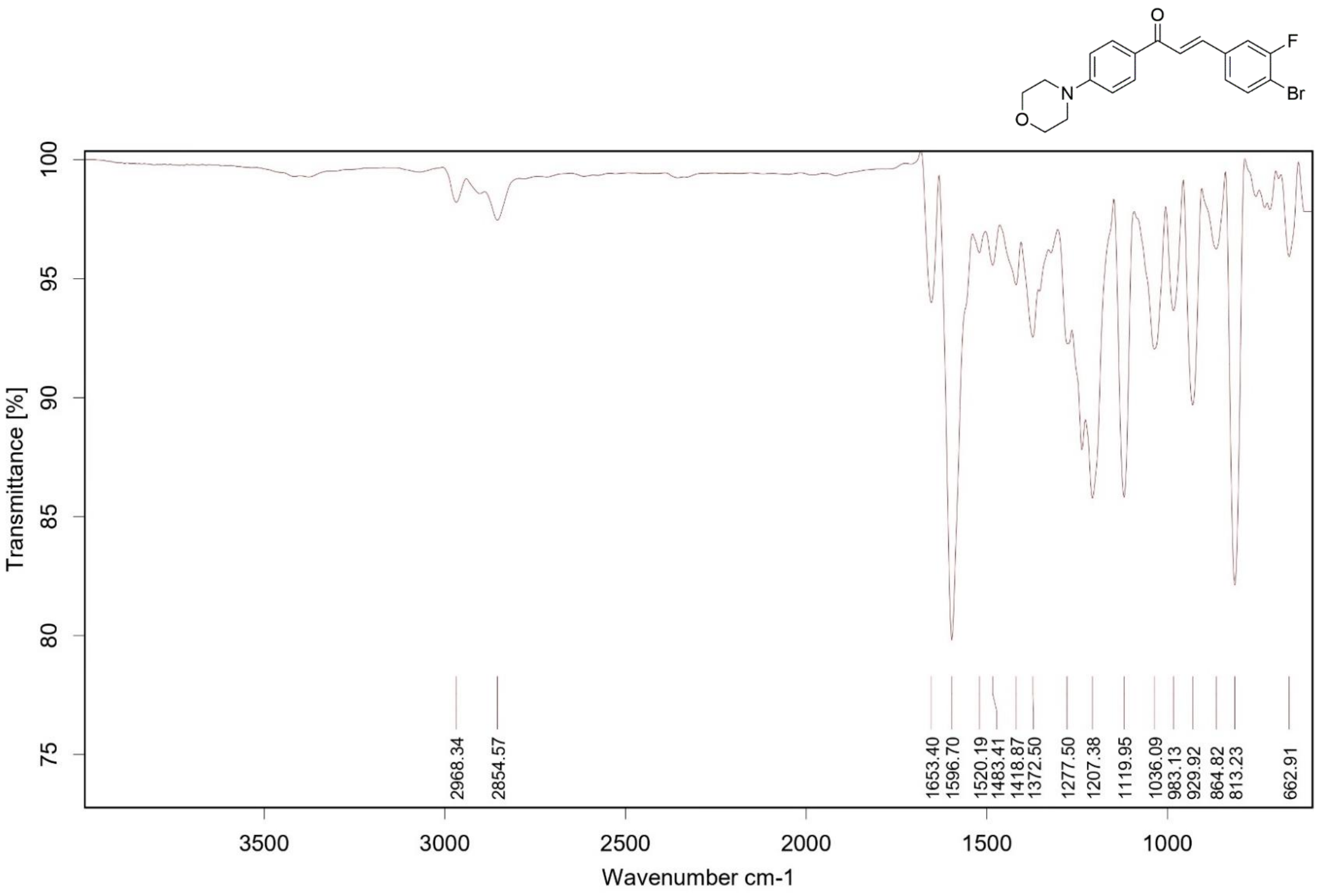

Figure S17. FT-IR of **C5**

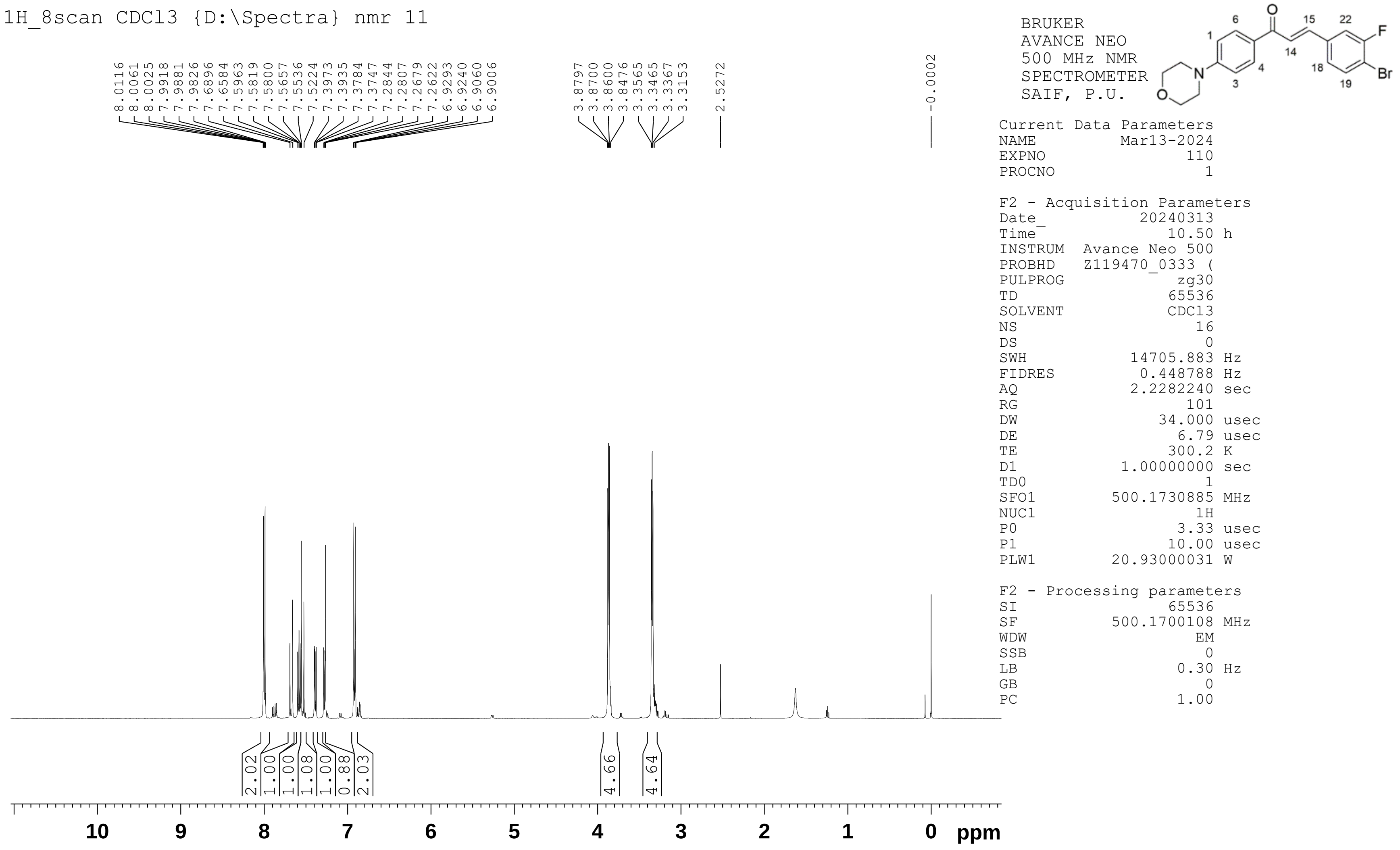

Figure S18. ^{1}H NMR of **C5**

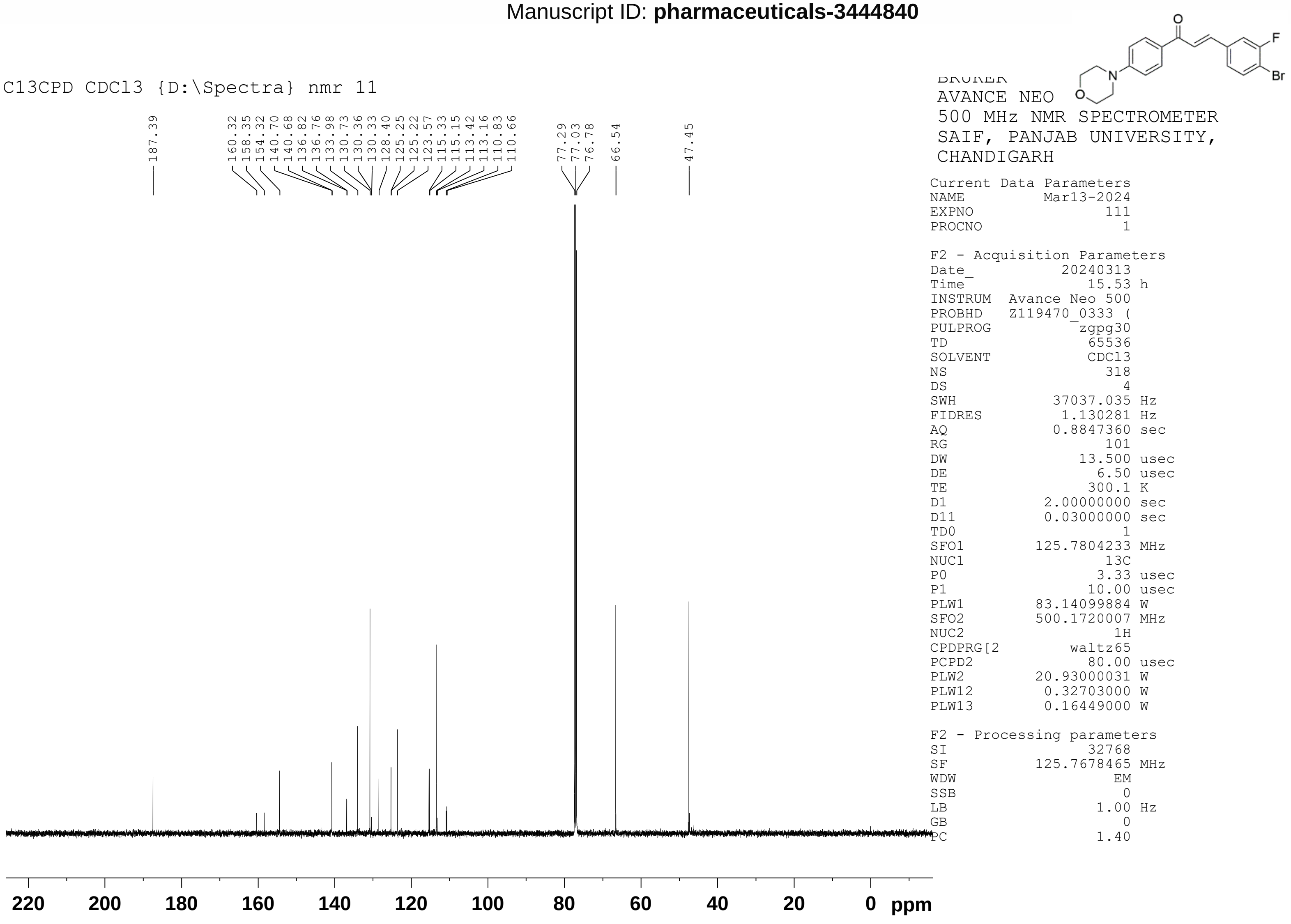

Figure S19. ^{13}C NMR of **C5**

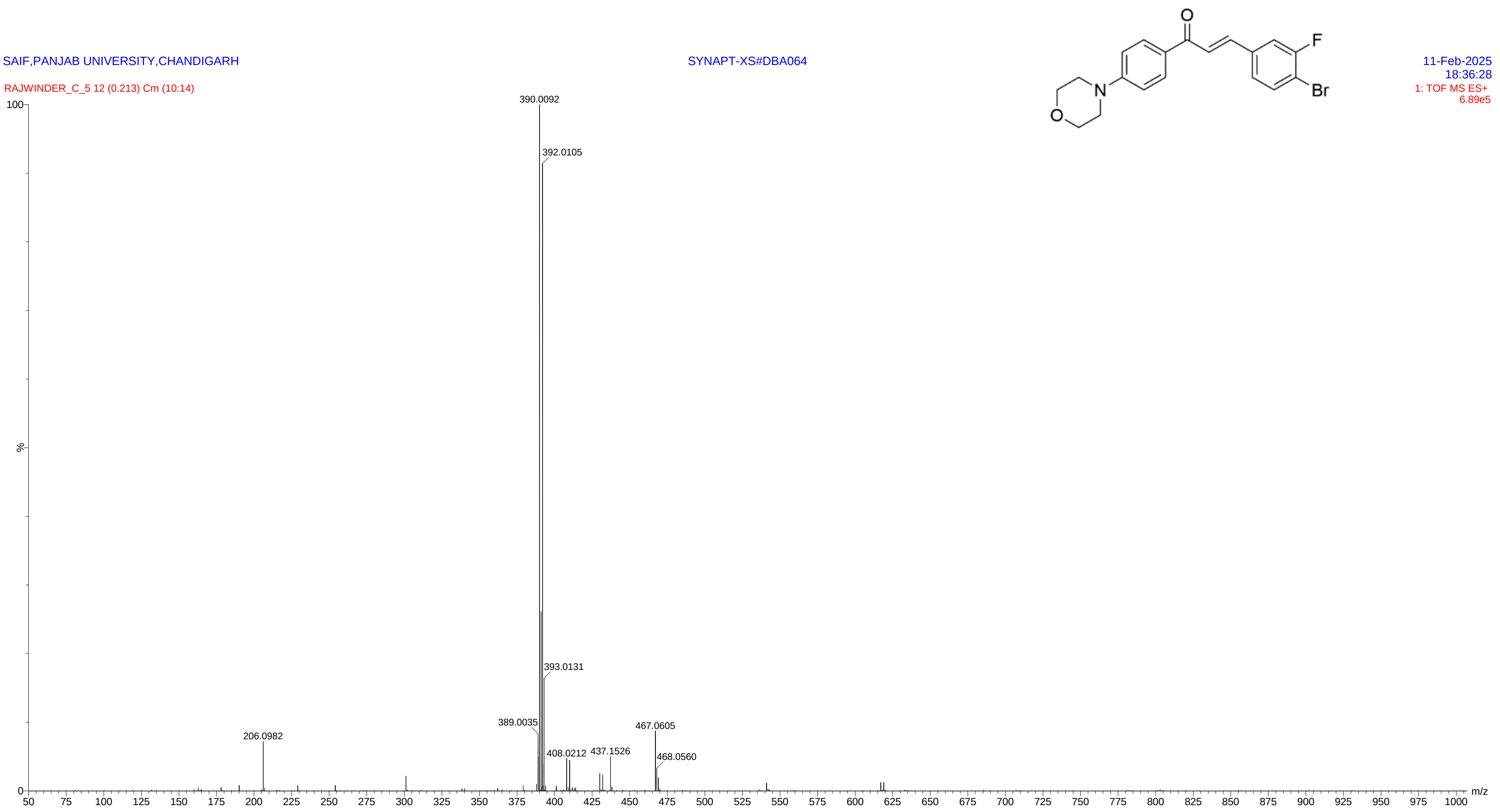

Figure S20. HRMS of **C5**

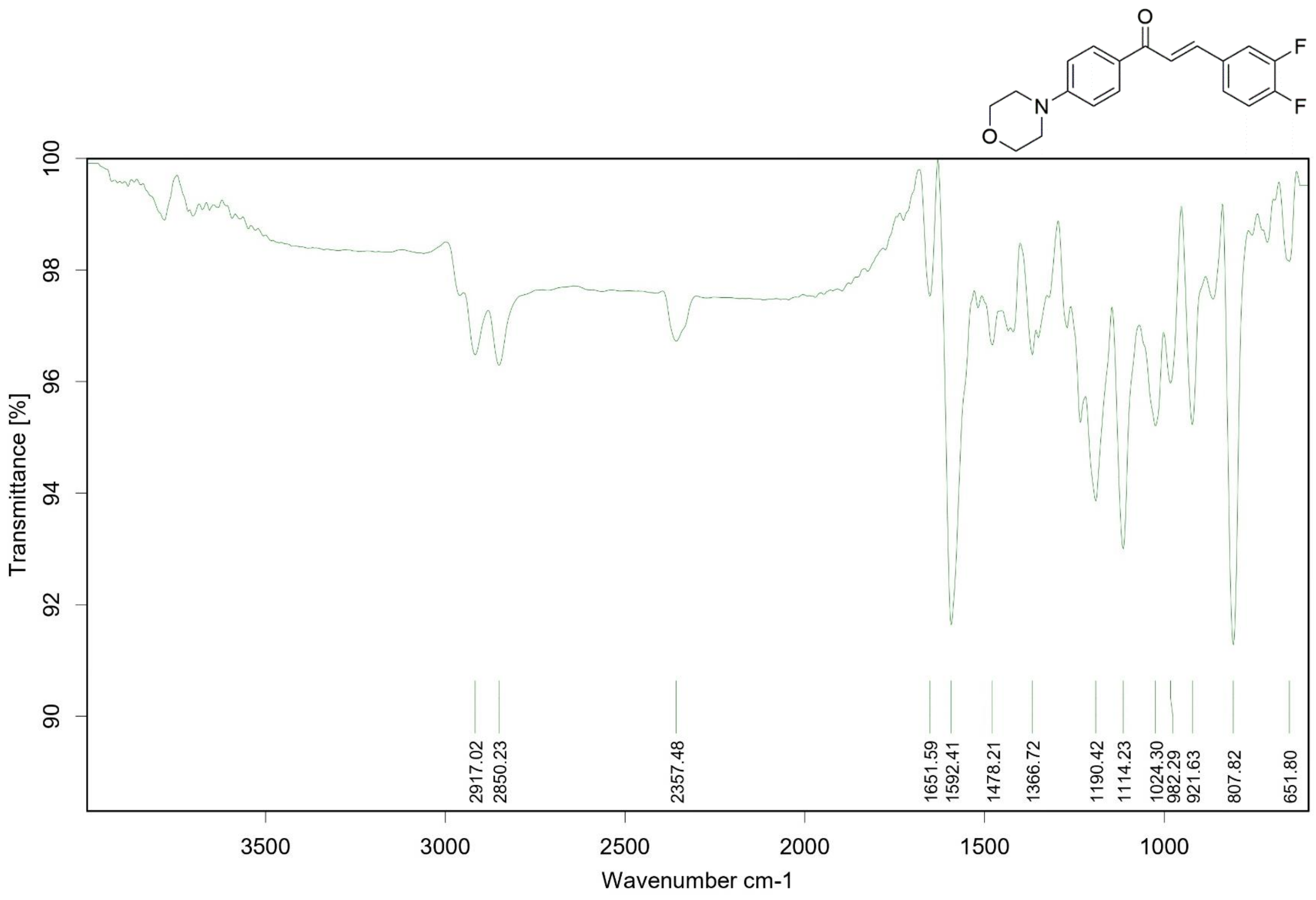

Figure S21. FT-IR of **C6**

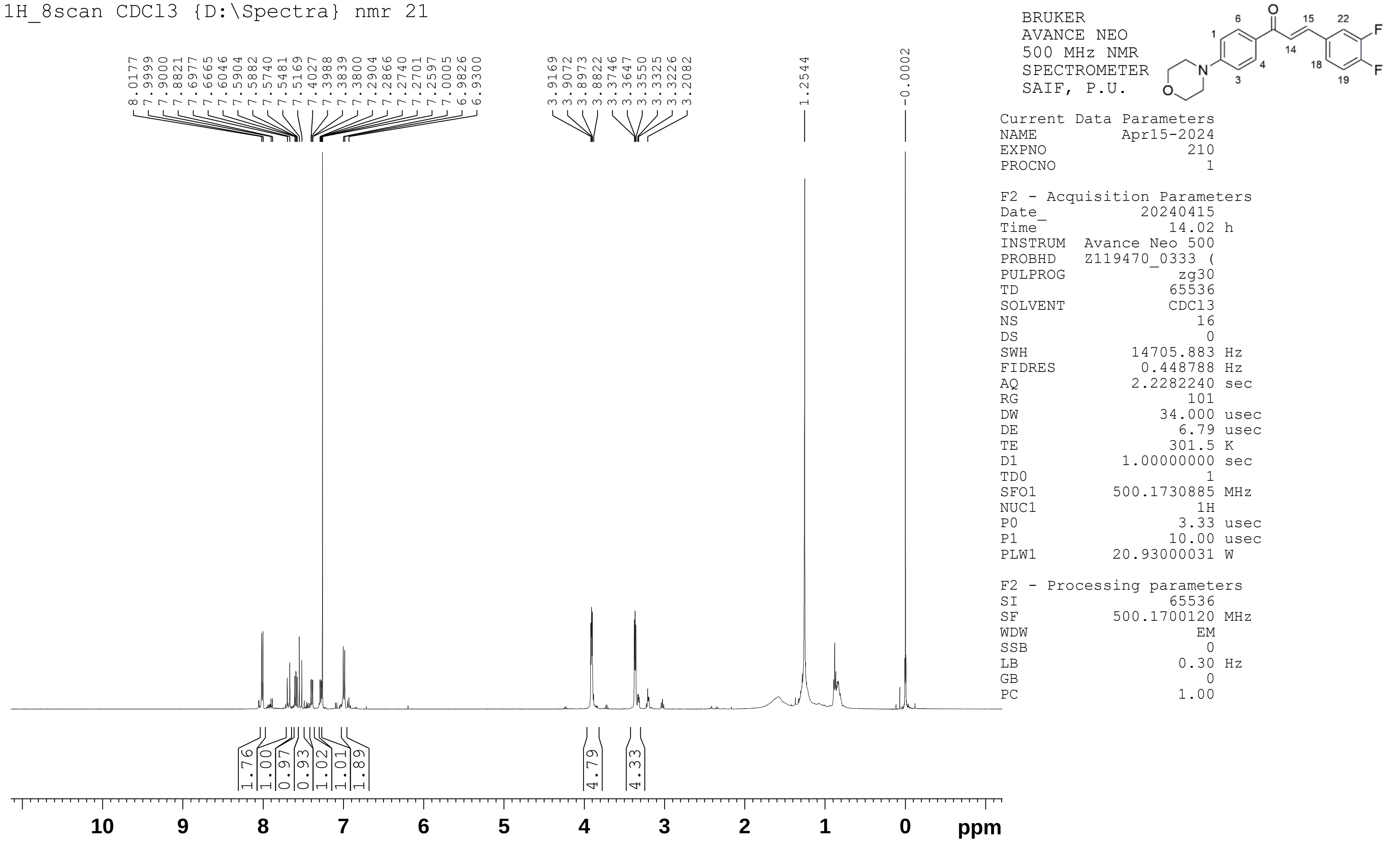

Figure S22. ^{1}H NMR of **C6**

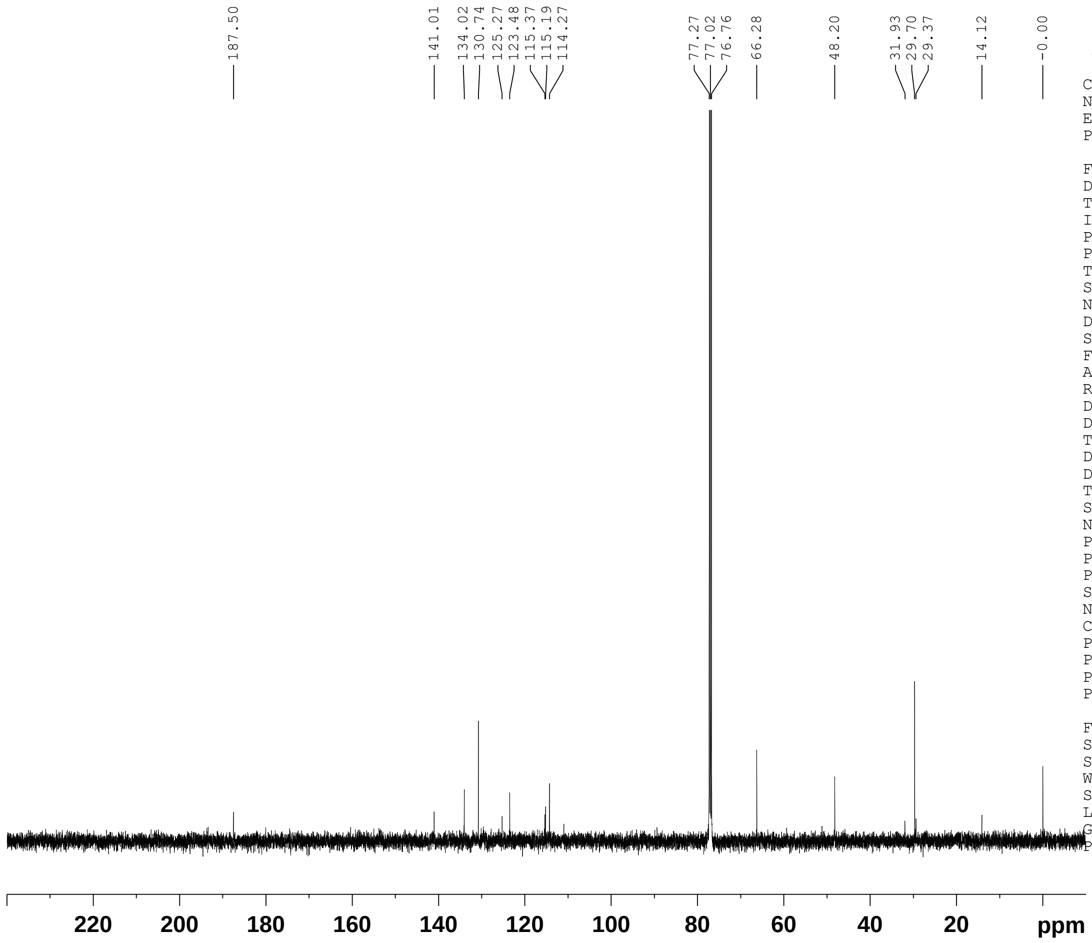

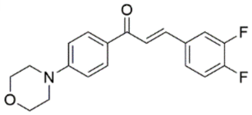

BRUKER
AVANCE NEO
500 MHz NMR SPECTROMETER
SAIF, PANJAB UNIVERSITY,
CHANDIGARH

Current Data Parameters

NAME	Apr15-2024	
EXPNO	211	
PROCNO	1	

F2 - Acquisition Parameters

Date_	20240415	
Time	21.14	h
INSTRUM	Avance Neo 500	
PROBHD	Z119470_0333 (	
PULPROG	zgpg30	
TD	65536	
SOLVENT	CDCl3	
NS	512	
DS	4	
SWH	37037.035	Hz
FIDRES	1.130281	Hz
AQ	0.8847360	sec
RG	101	
DW	13.500	usec
DE	6.50	usec
TE	300.2	K
D1	2.00000000	sec
D11	0.03000000	sec
TD0	1	
SFO1	125.7804233	MHz
NUC1	13C	
P0	3.33	usec
P1	10.00	usec
PLW1	83.14099884	W
SFO2	500.1720007	MHz
NUC2	1H	
CPDPRG[2	waltz65	
PCPD2	80.00	usec
PLW2	20.93000031	W
PLW12	0.32703000	W
PLW13	0.16449000	W

F2 - Processing parameters

SI	32768	
SF	125.7678462	MHz
WDW	EM	
SSB	0	
LB	1.00	Hz
GB	0	
PC	1.40	

Figure S23. ^{13}C NMR of **C6**

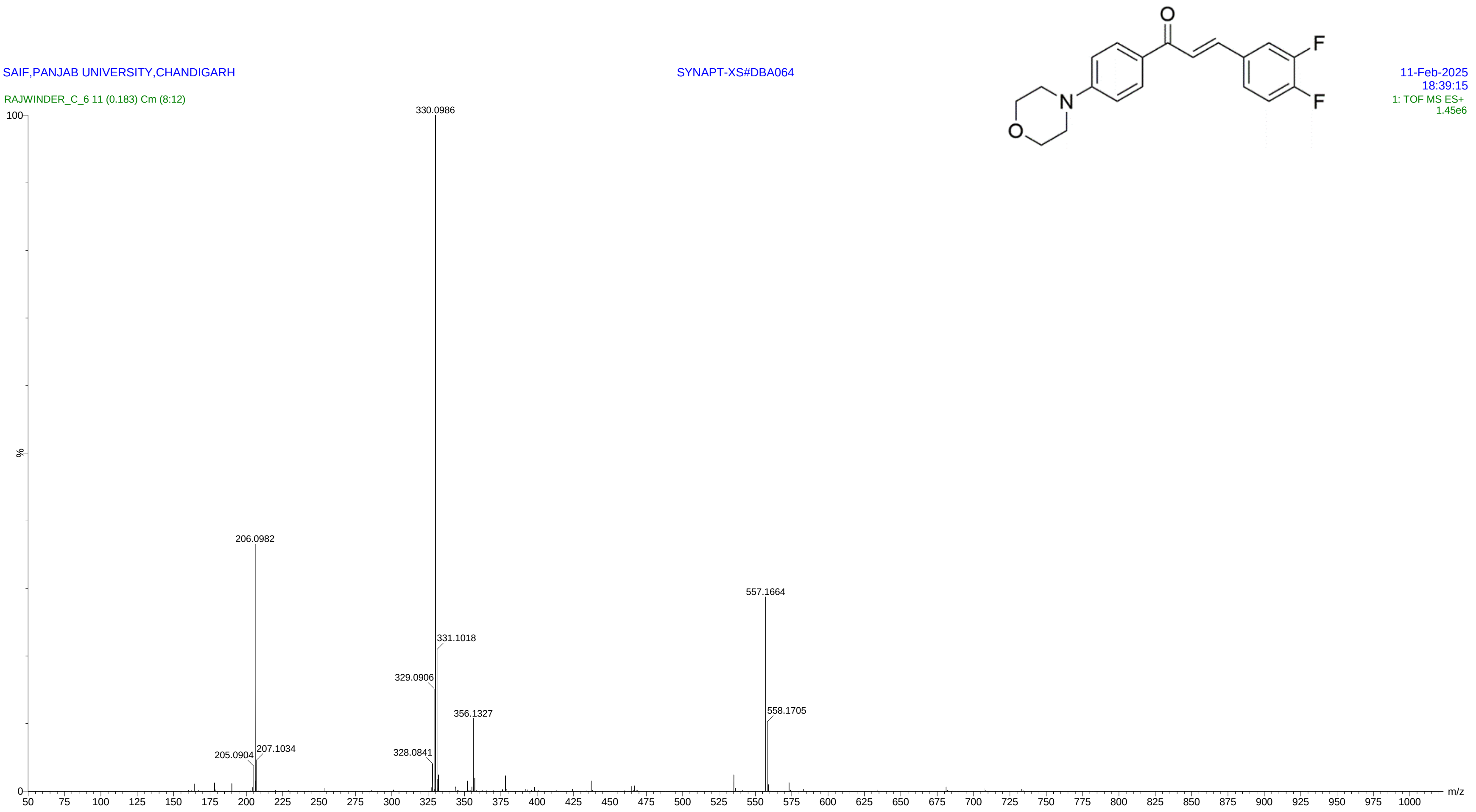

Figure S24. HRMS of **C6**

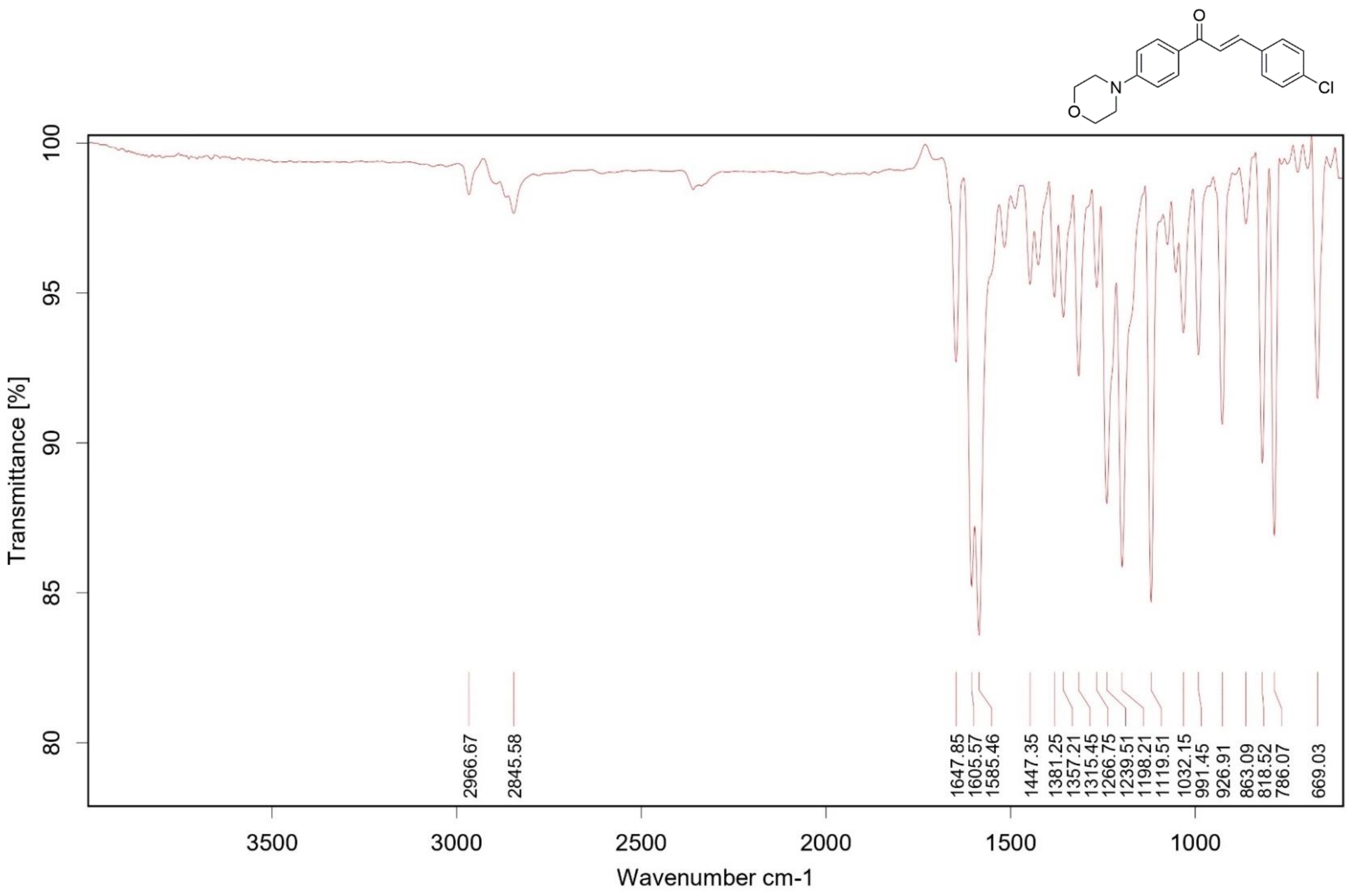

Figure S25. FT-IR of **C7**

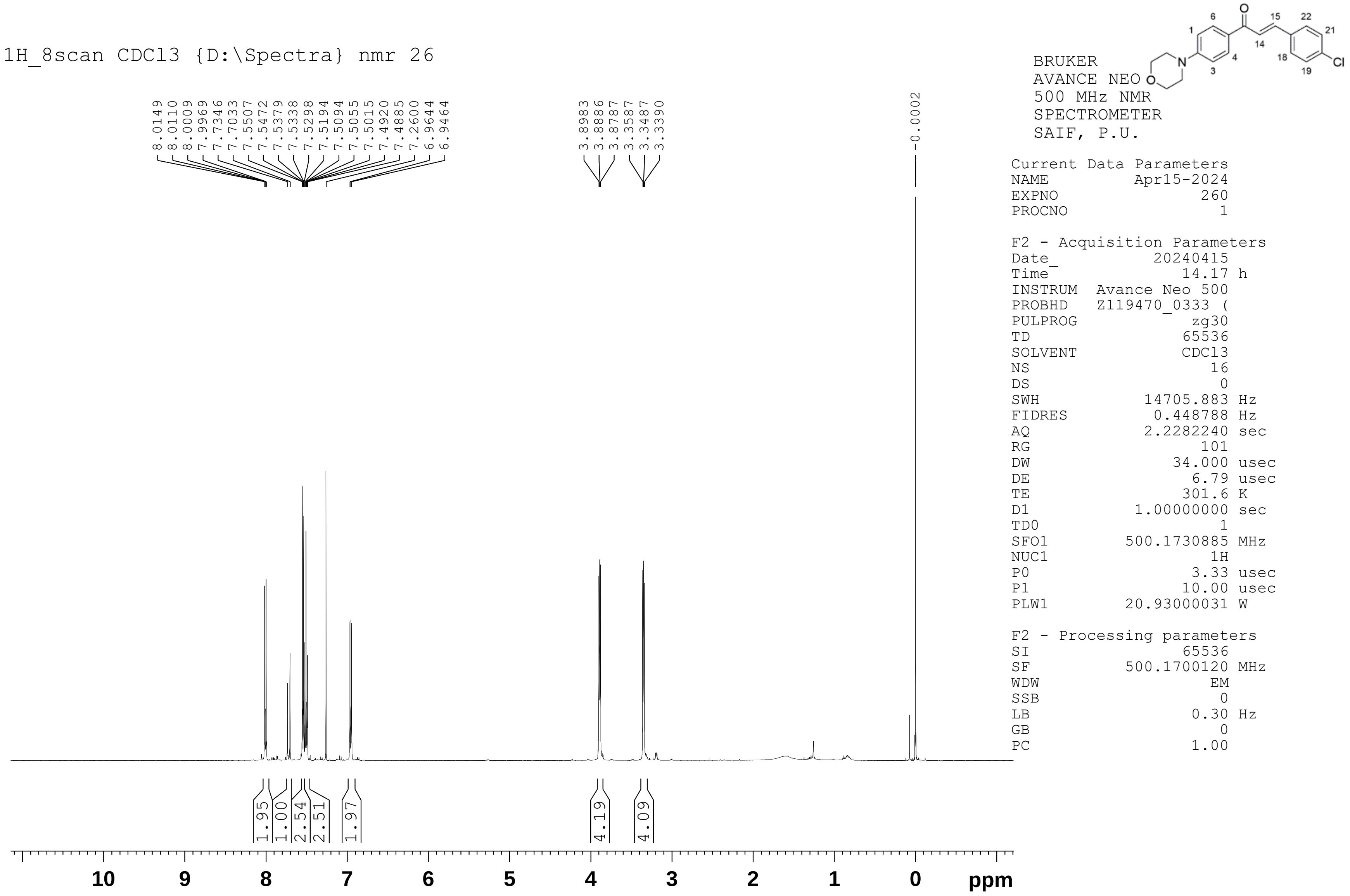

Figure S26. ^{1}H NMR of **C7**

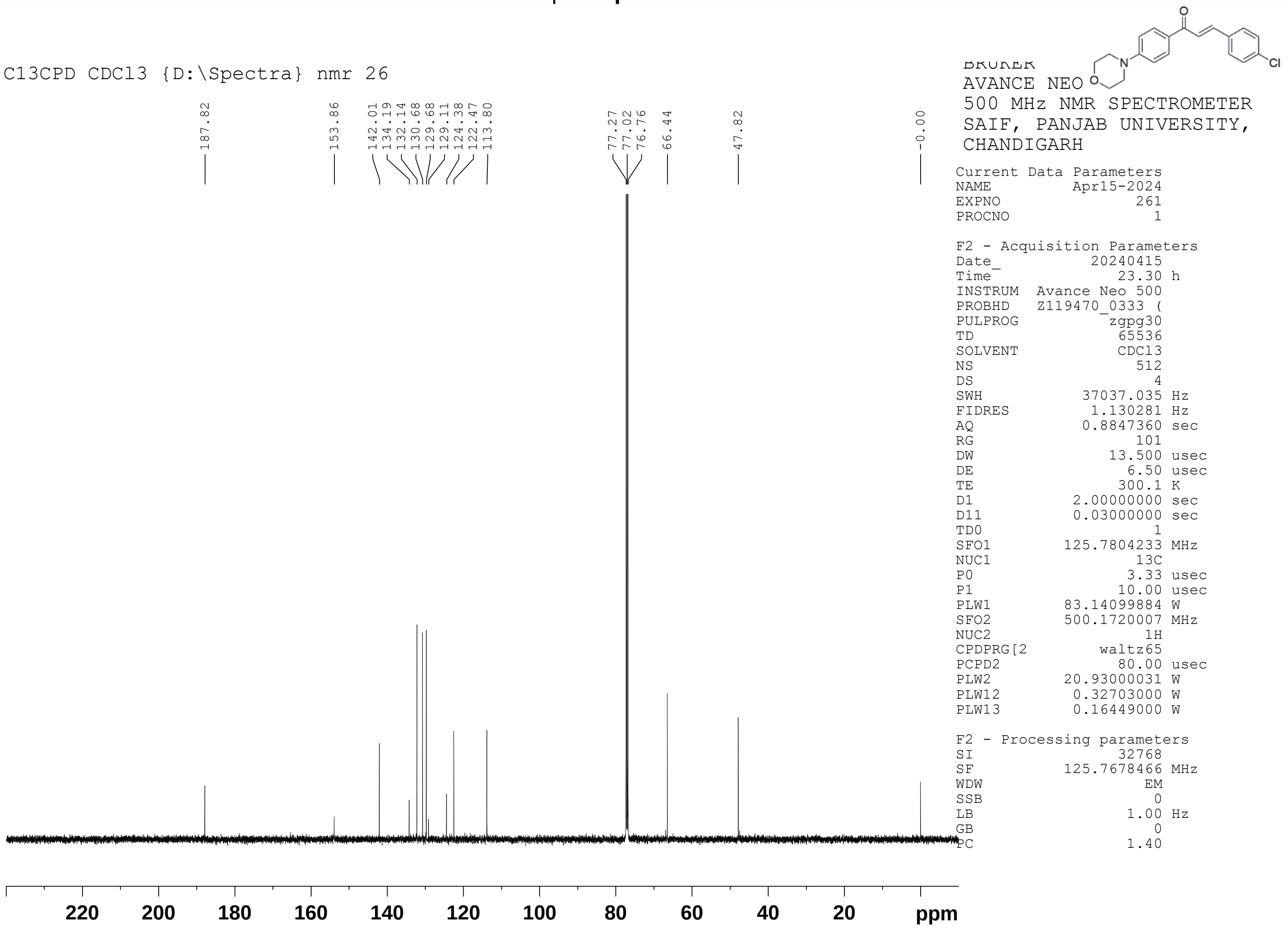

Figure S27. ^{13}C NMR of **C7**

Manuscript ID: **pharmaceuticals-3444840**

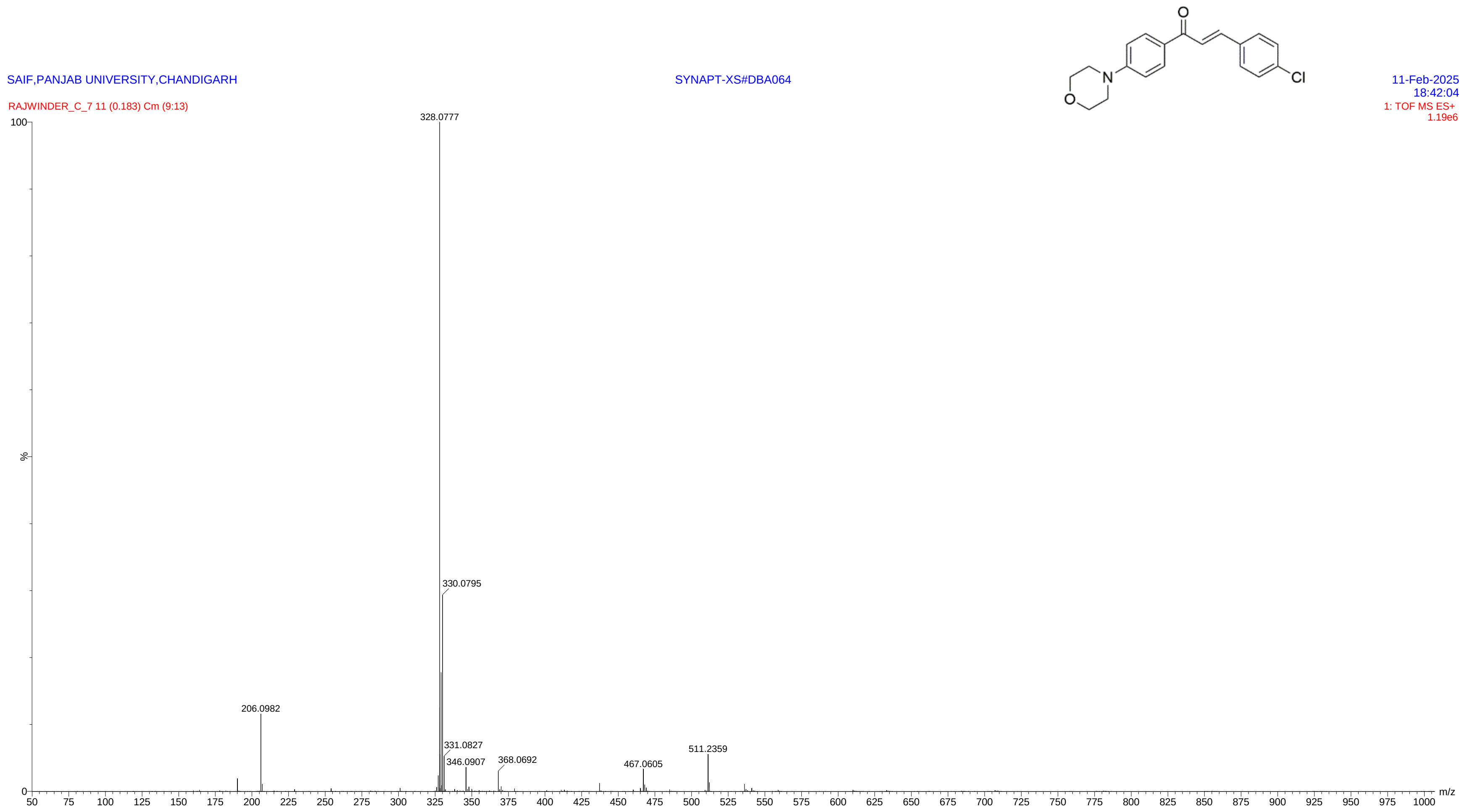

Figure S28. HRMS of **C7**

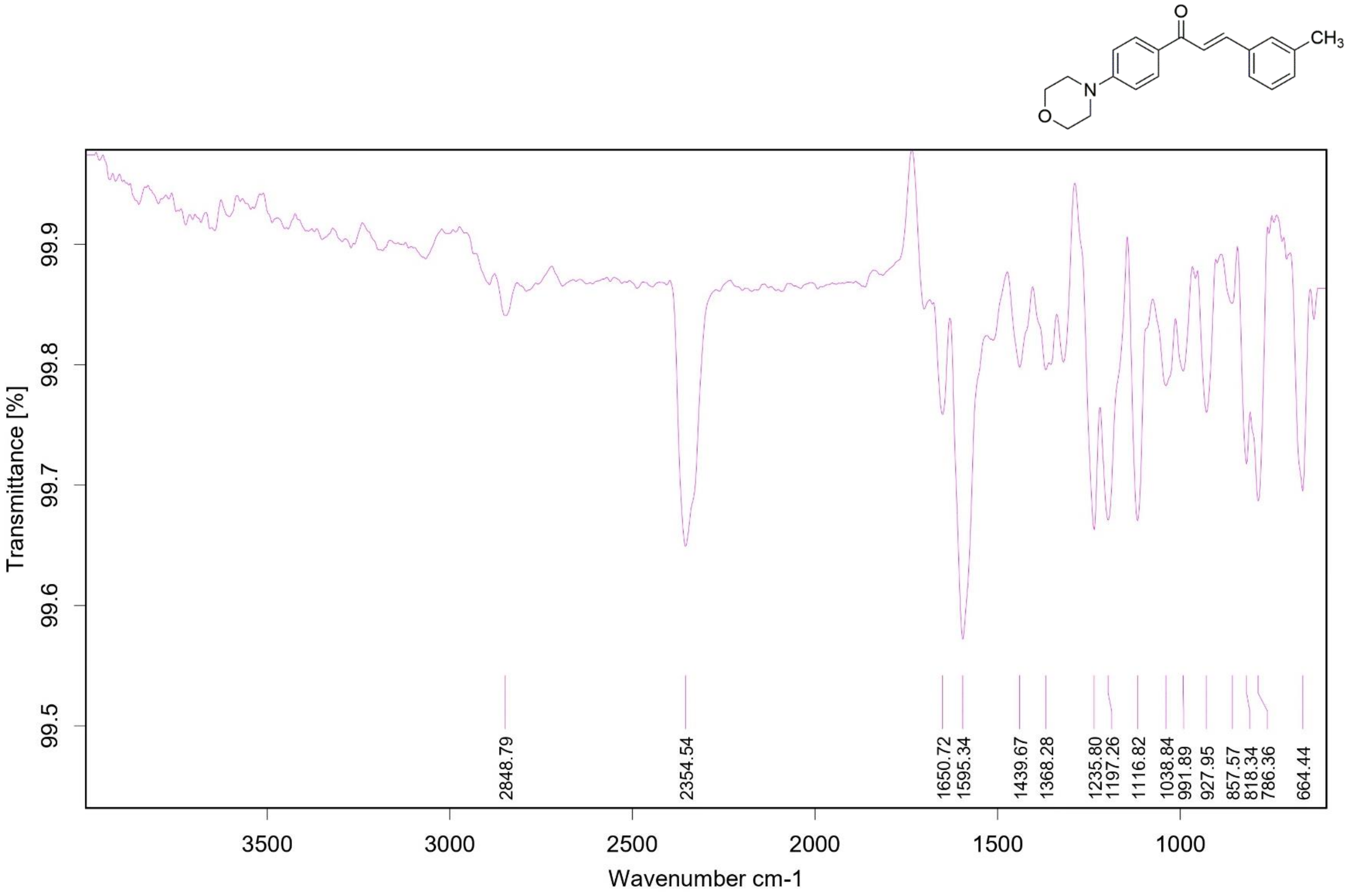

Figure S29. FT-IR of **C8**

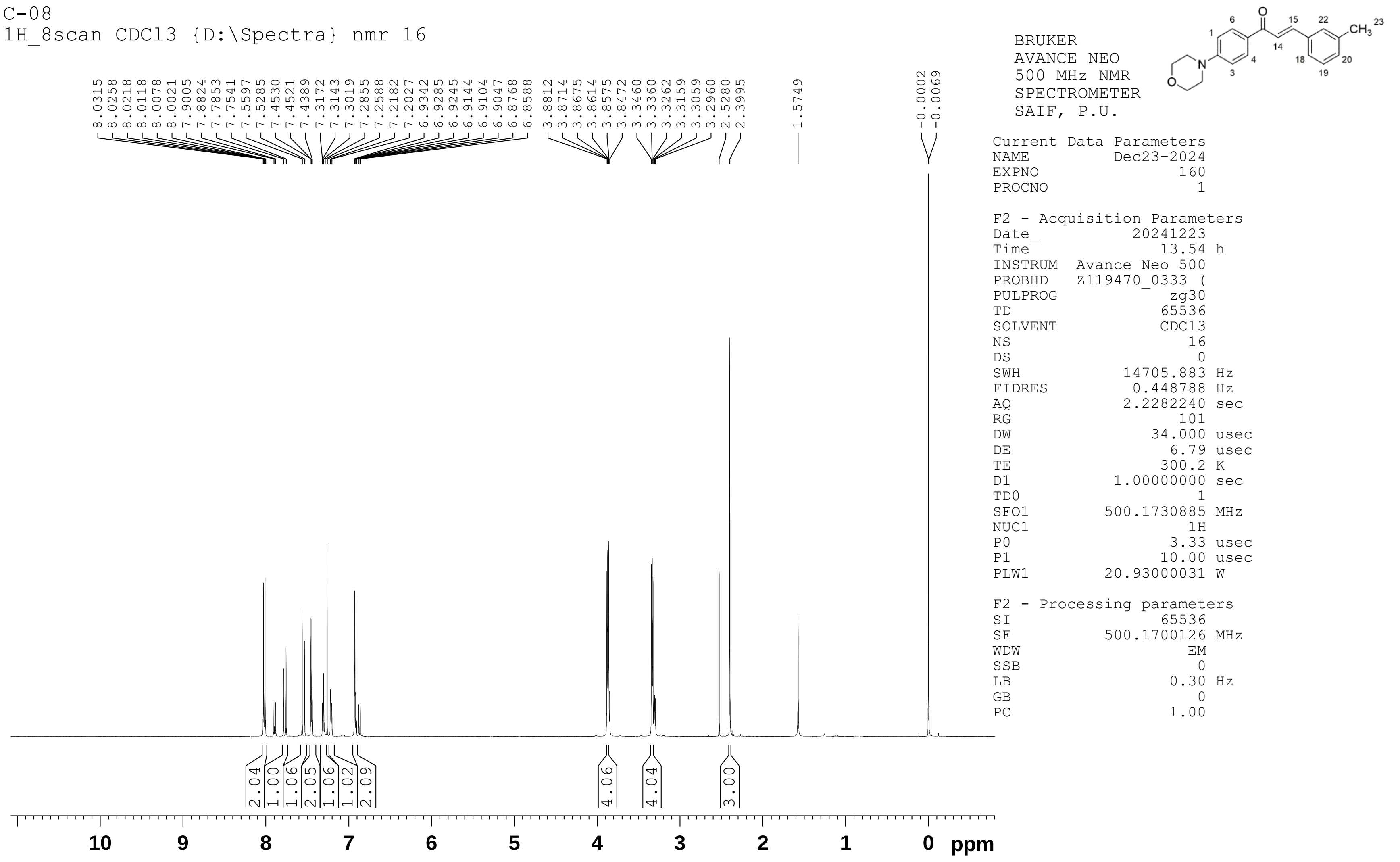

Figure S30. ^{1}H NMR of **C8**

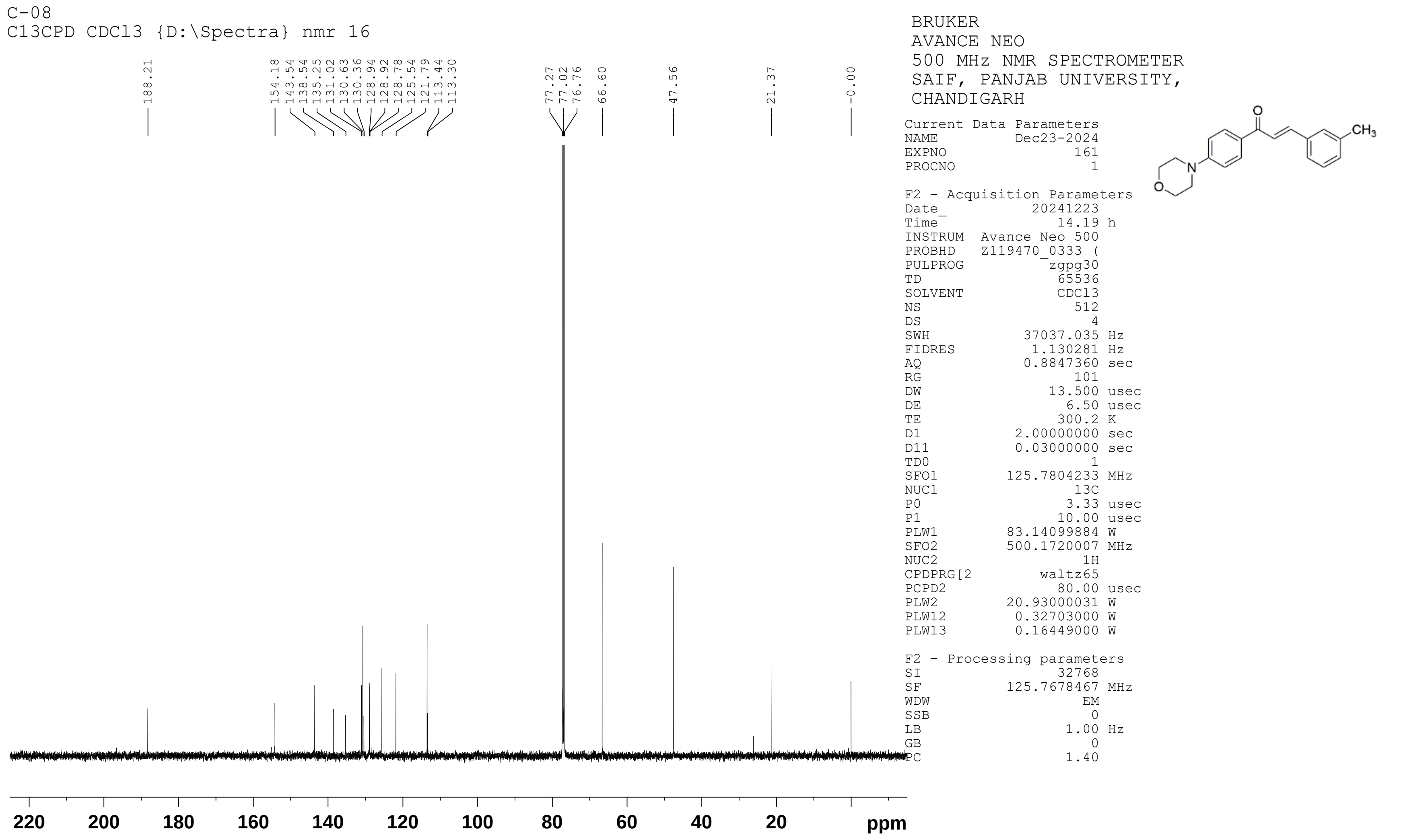

Figure S31. ^{13}C NMR of **C8**

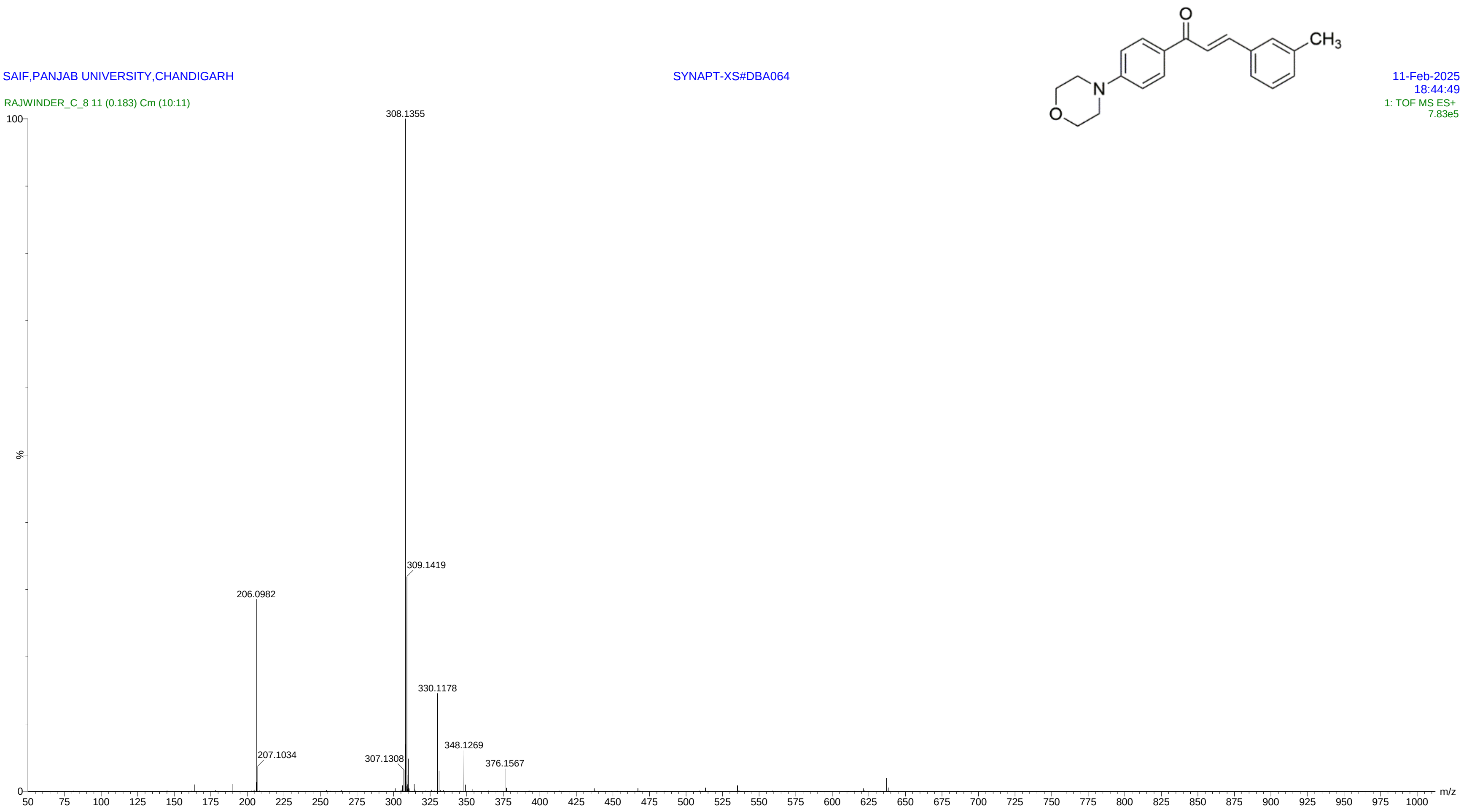

Figure S32. HRMS of **C8**

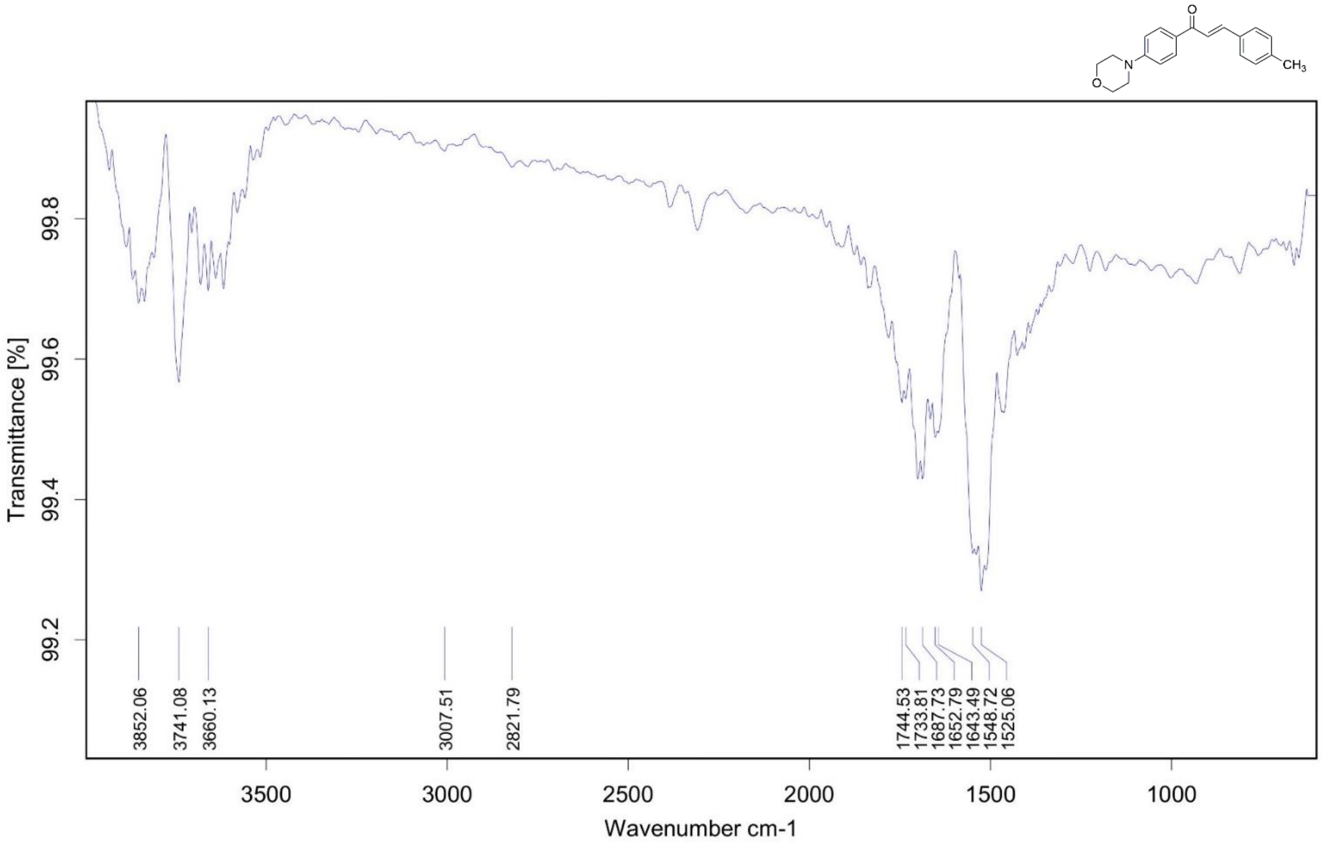

Figure S33. FT-IR of **C9**

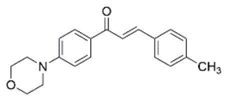

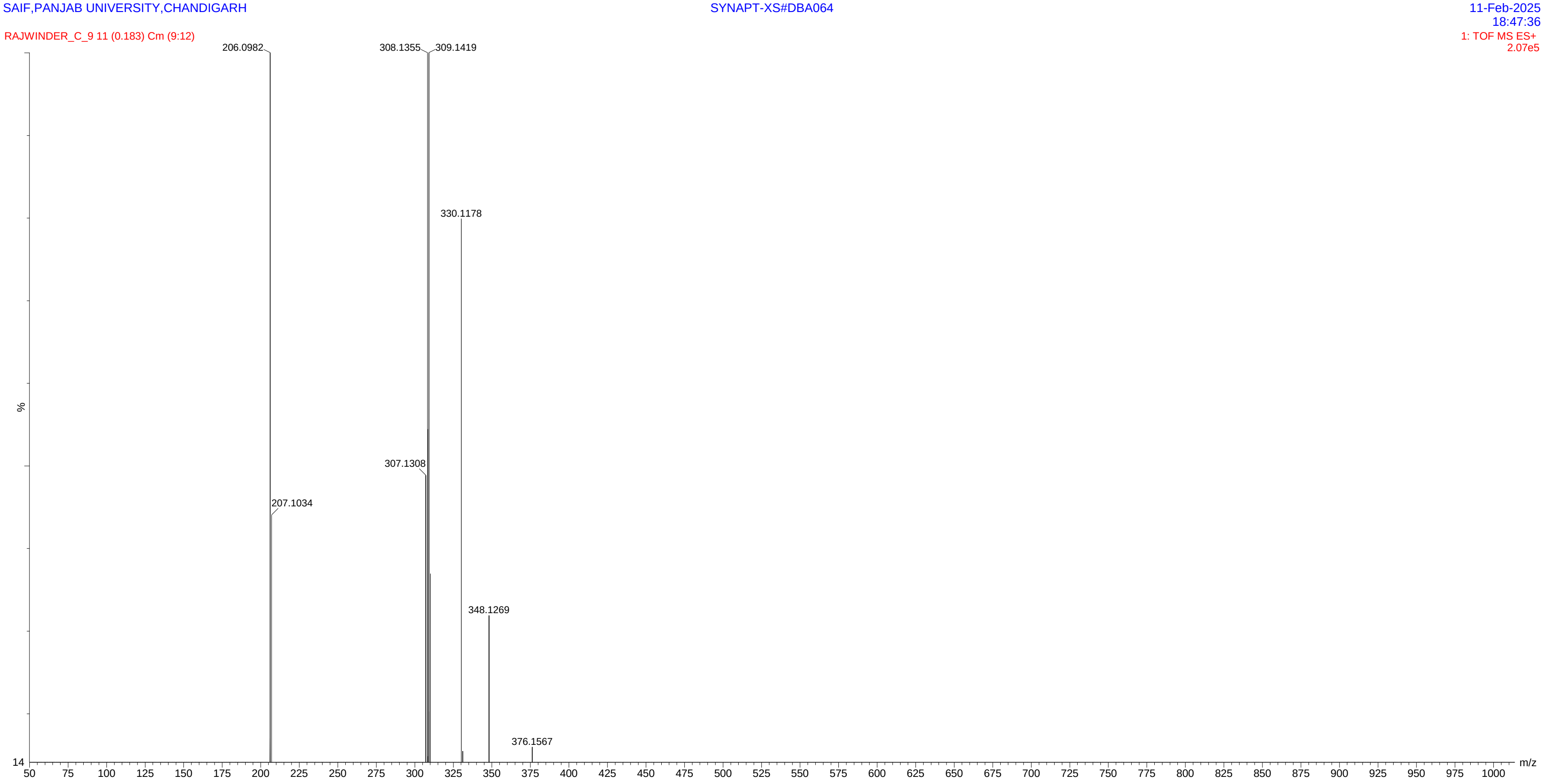

Figure S34. HRMS of **C9**

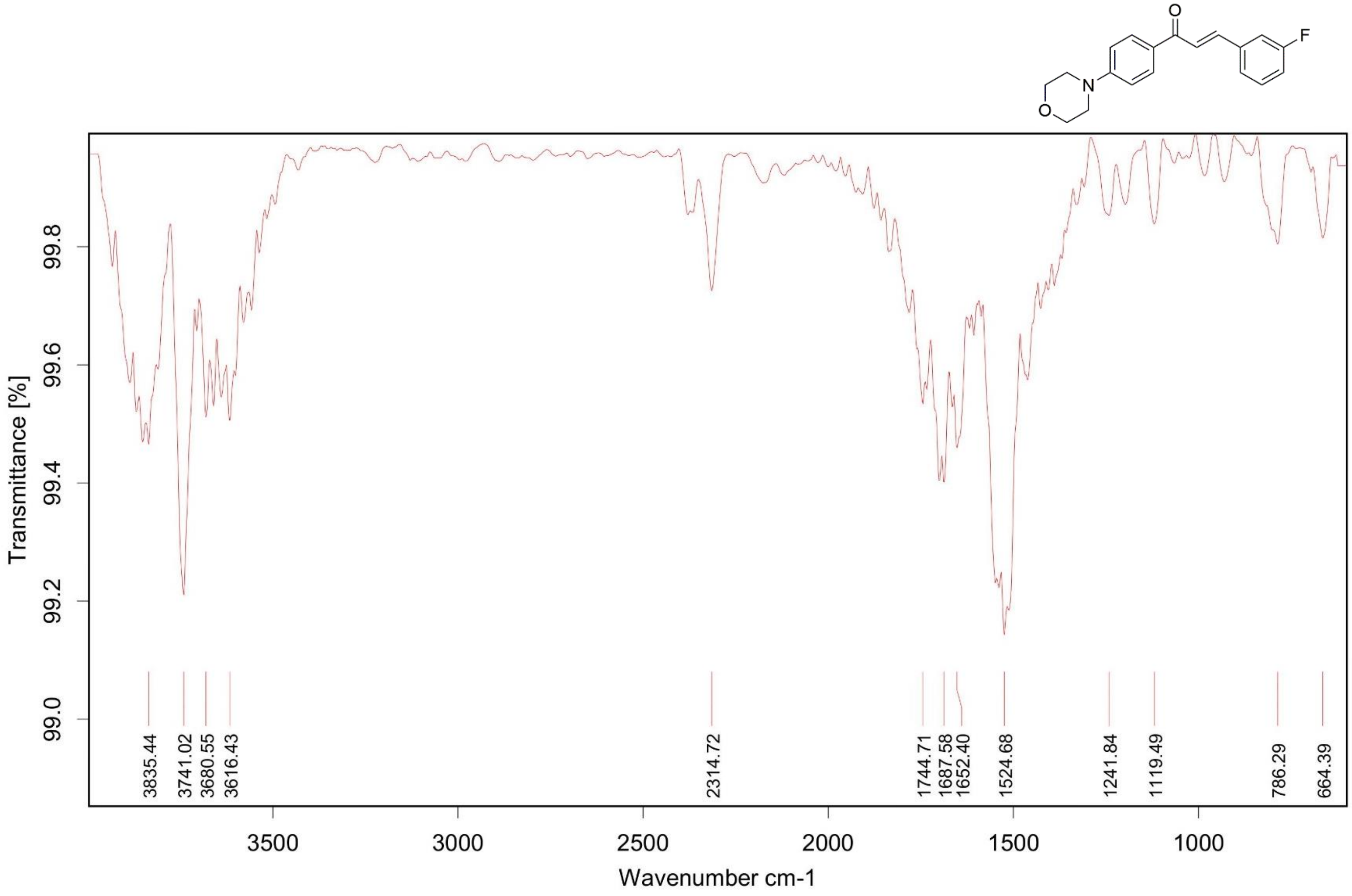

Figure S35. FT-IR of **C10**

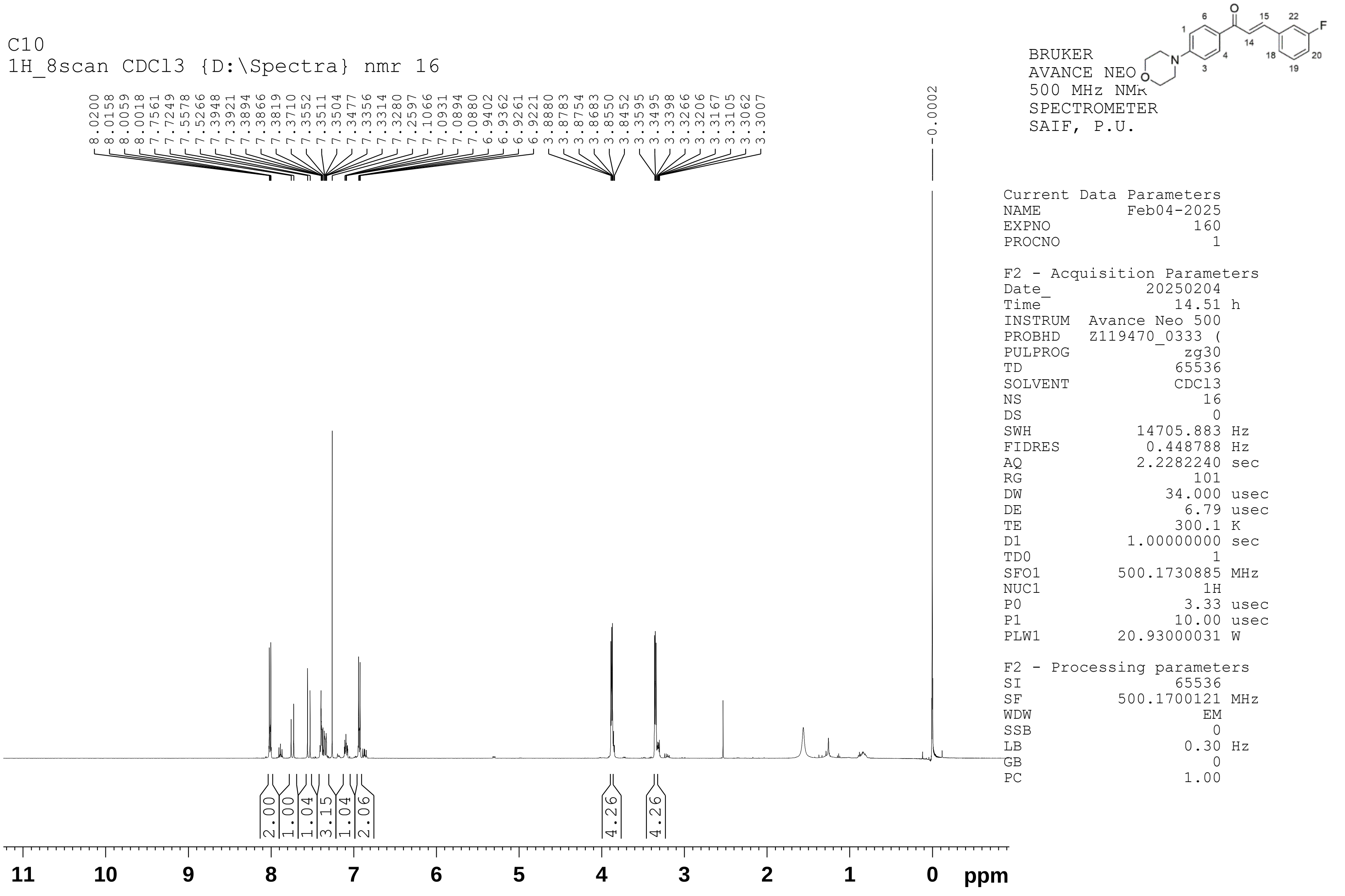

Figure S36. ^{1}H NMR of **C10**

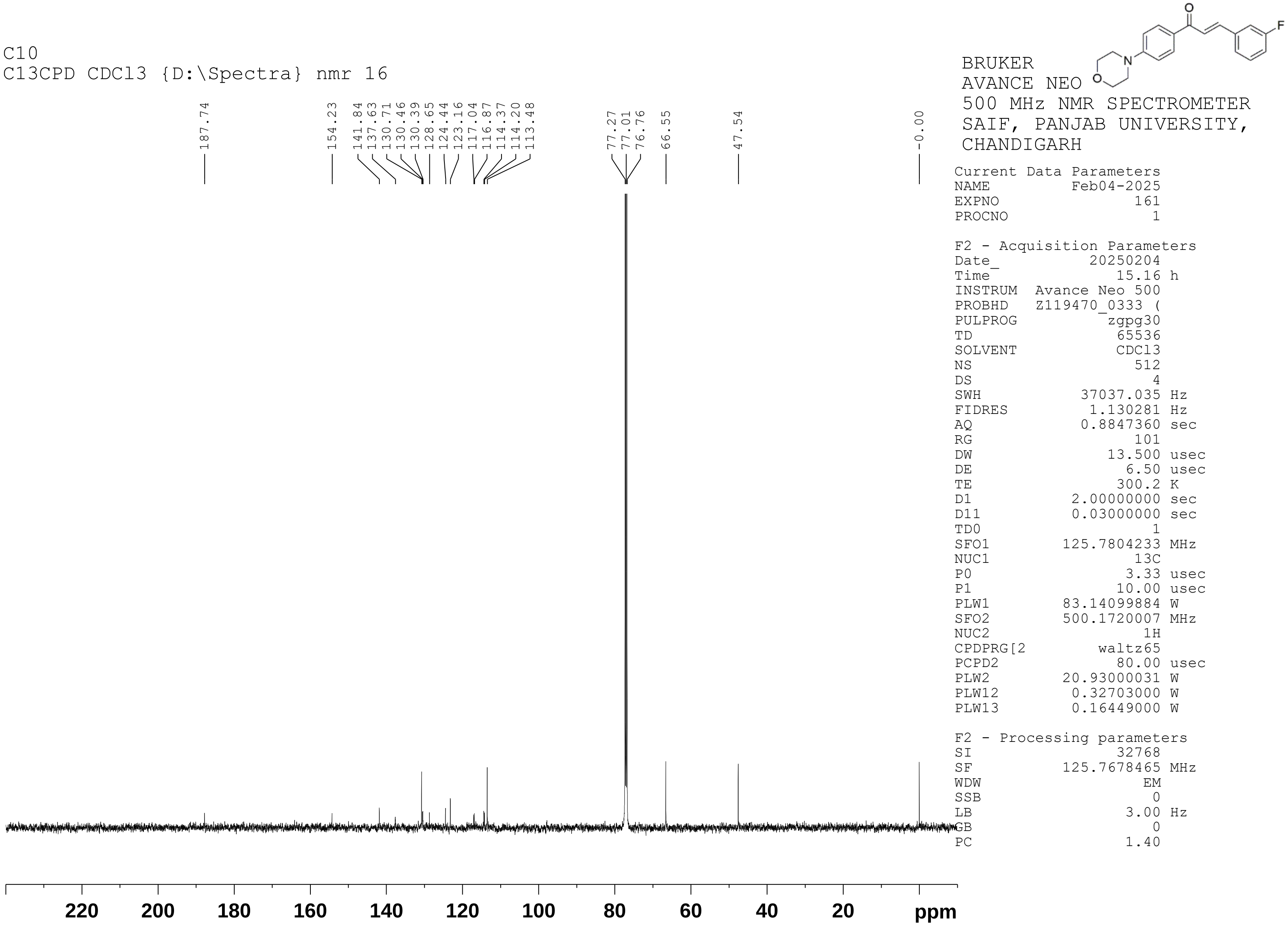

Figure S37. ^{13}C NMR of **C10**

Manuscript ID: **pharmaceuticals-3444840**

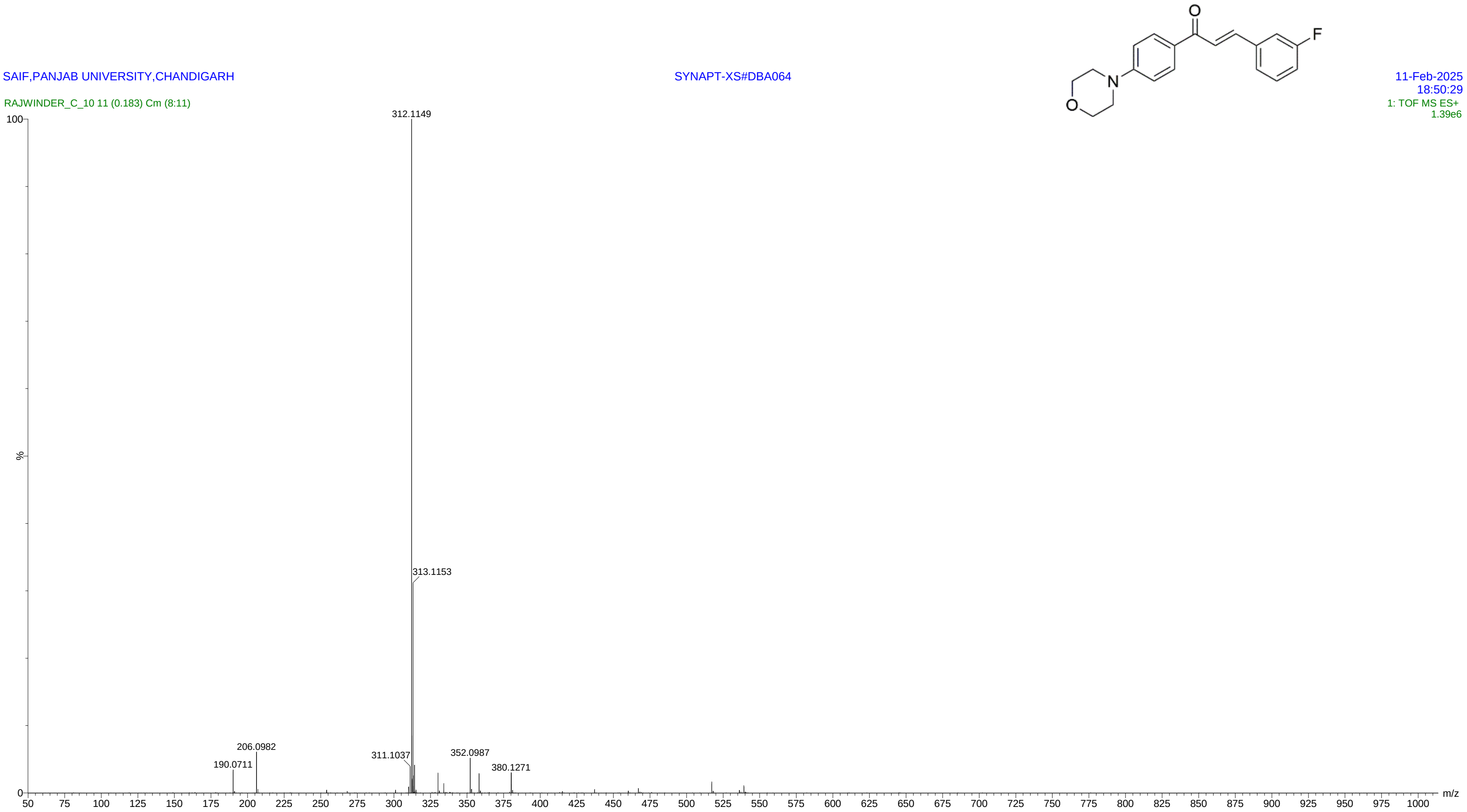

Figure S38. HRMS of **C10**

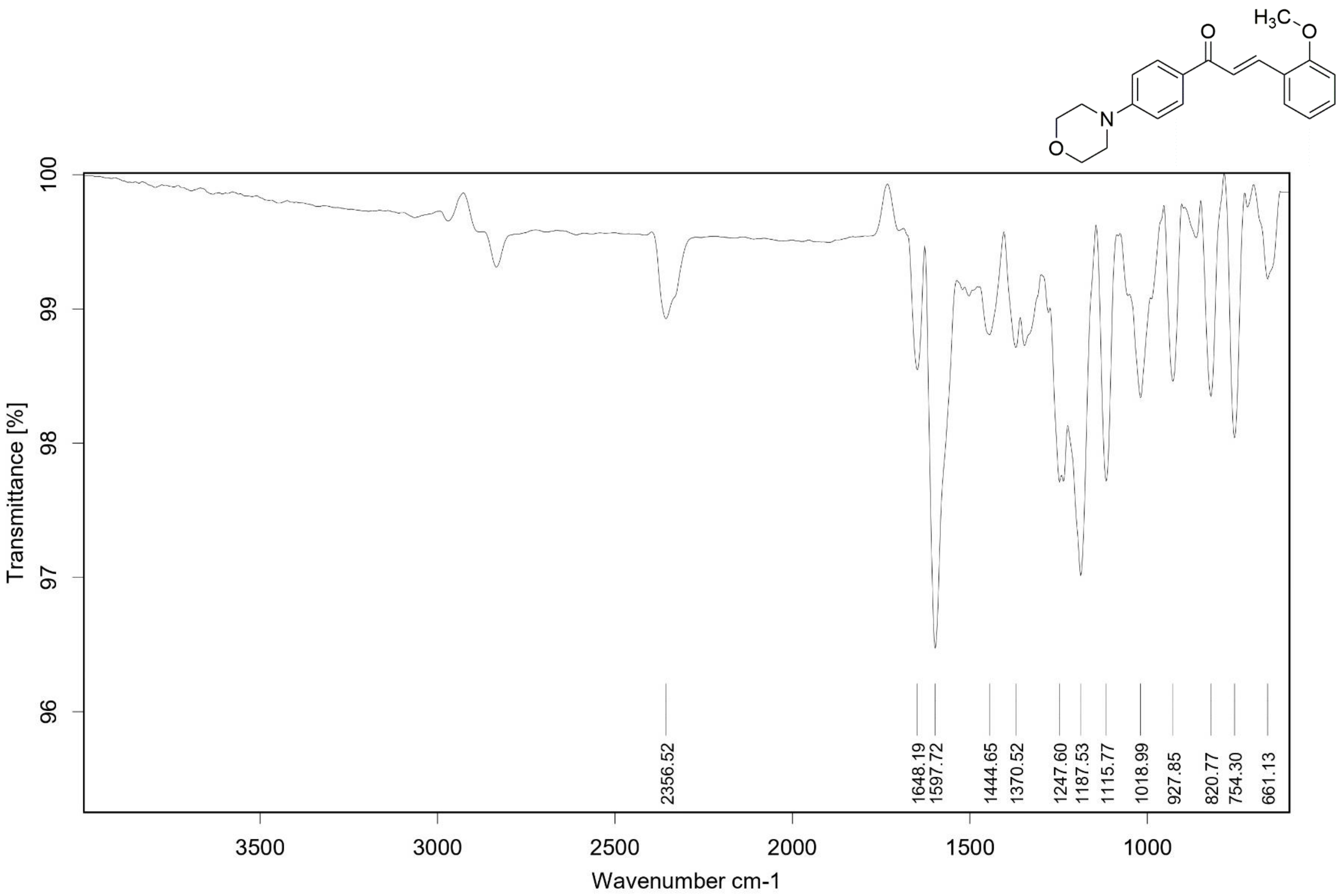

Figure S39. FT-IR of **C11**

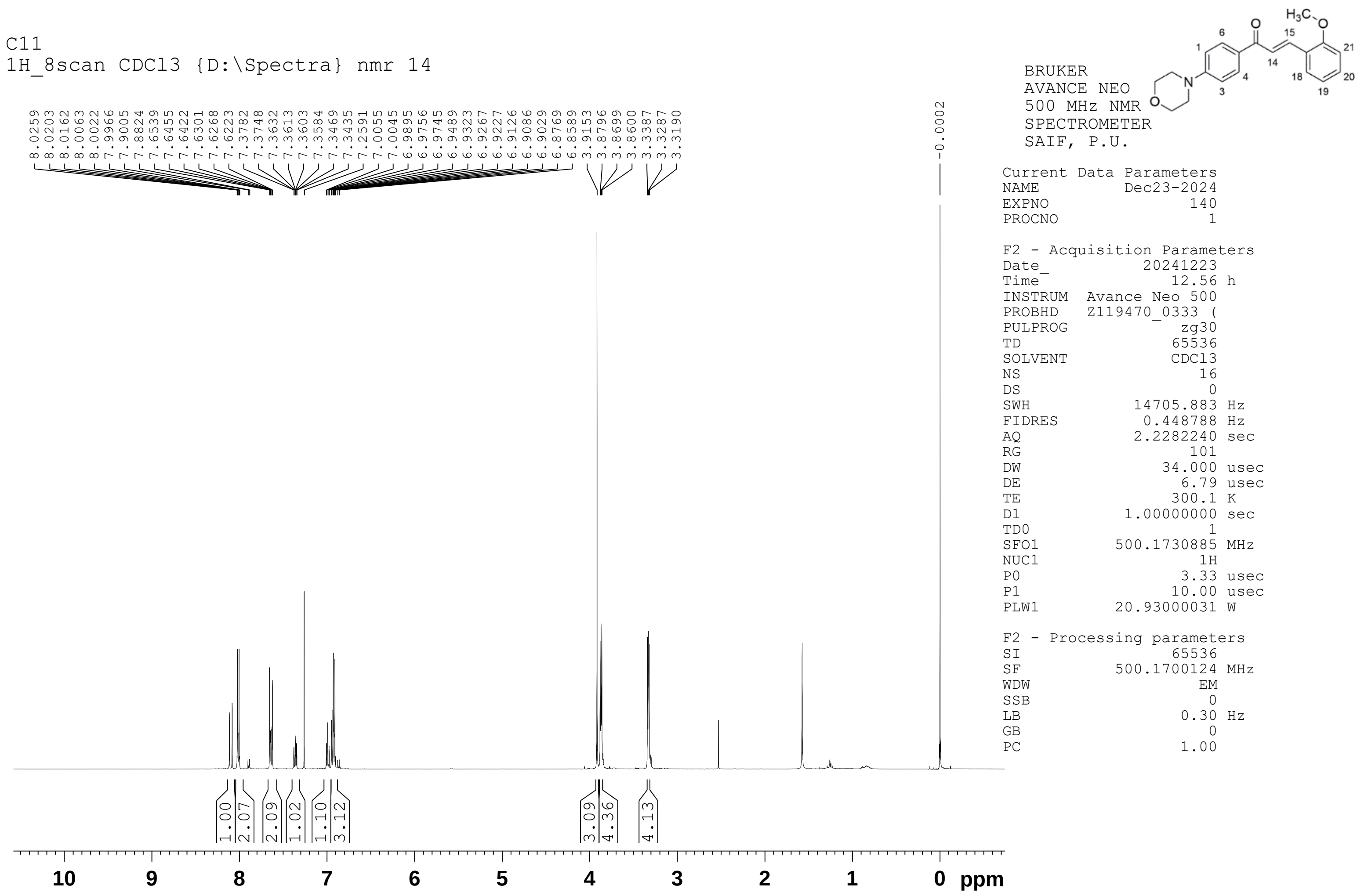

Figure S40. ^{1}H NMR of **C11**

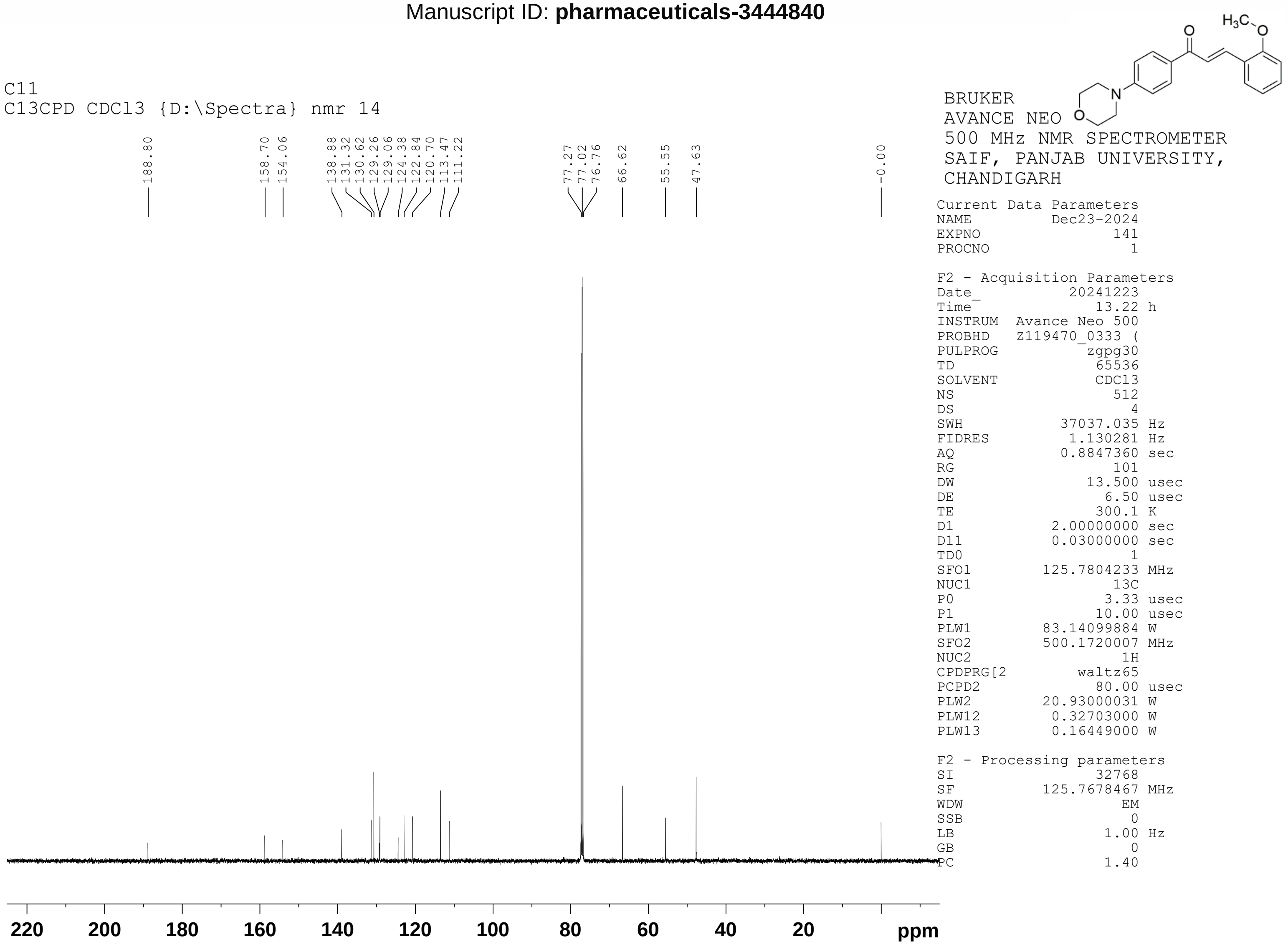

Figure S41. ^{13}C NMR of **C11**

Manuscript ID: **pharmaceuticals-3444840**

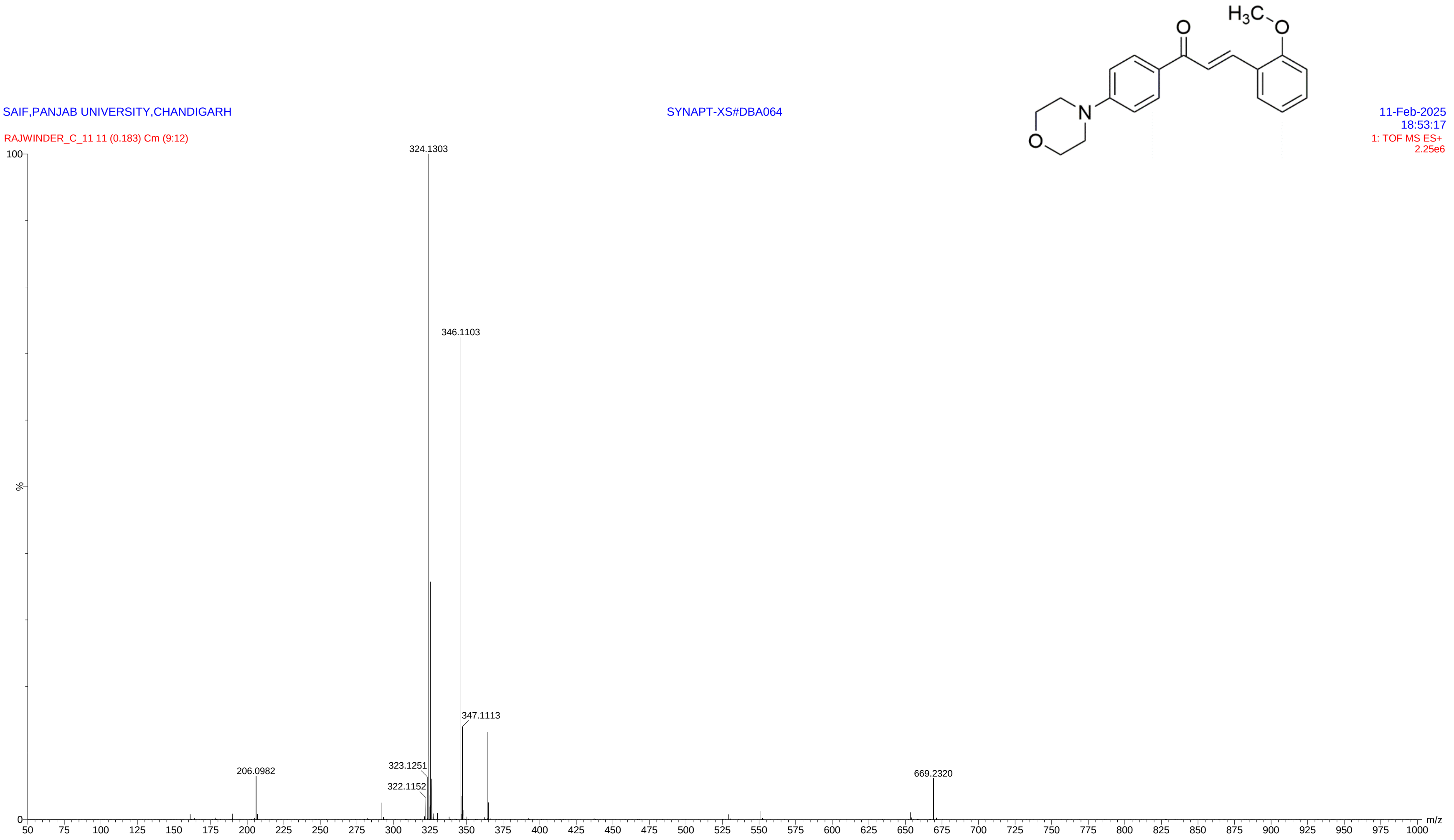

Figure S42. HRMS of **C11**

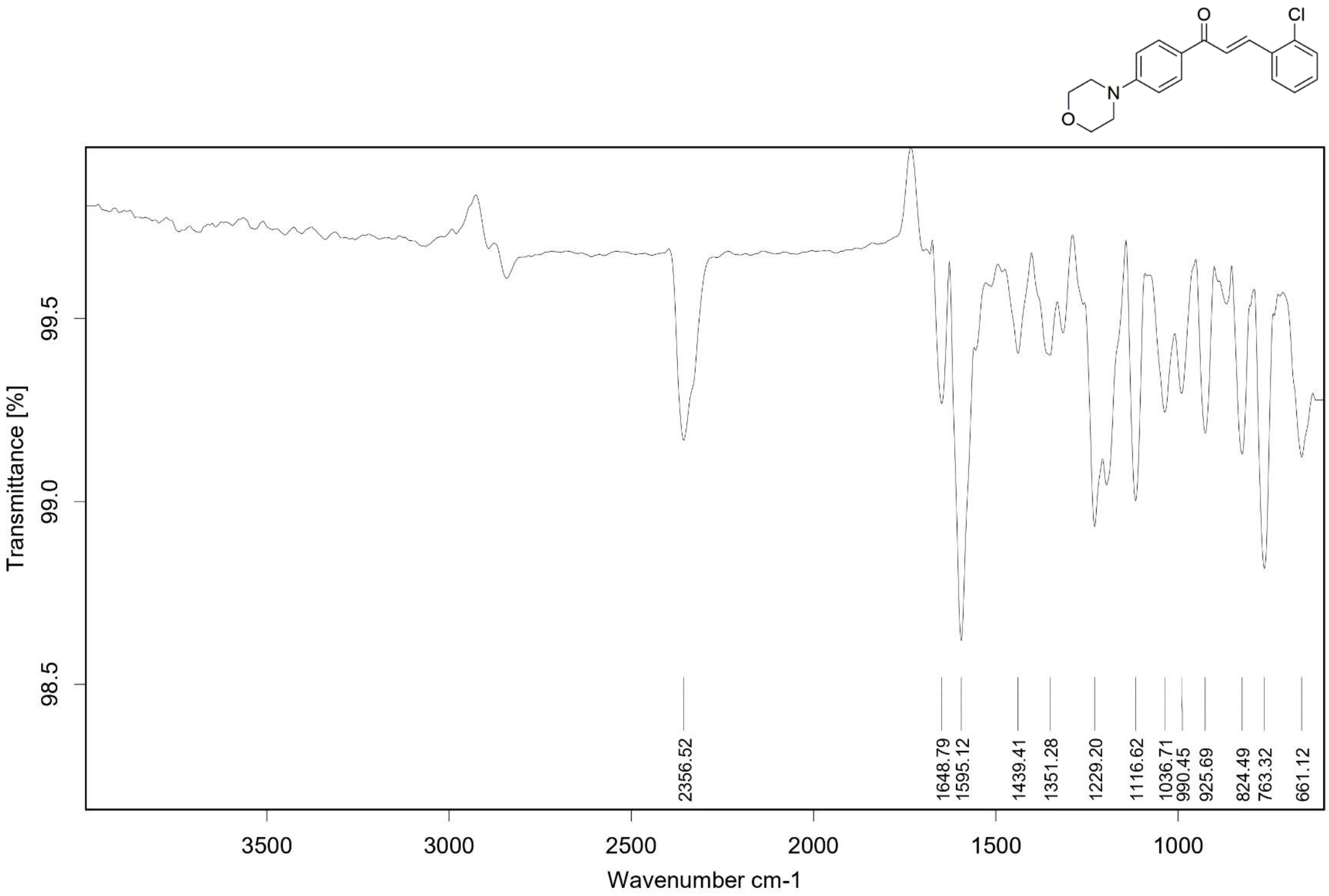

Figure S43. FT-IR of **C12**

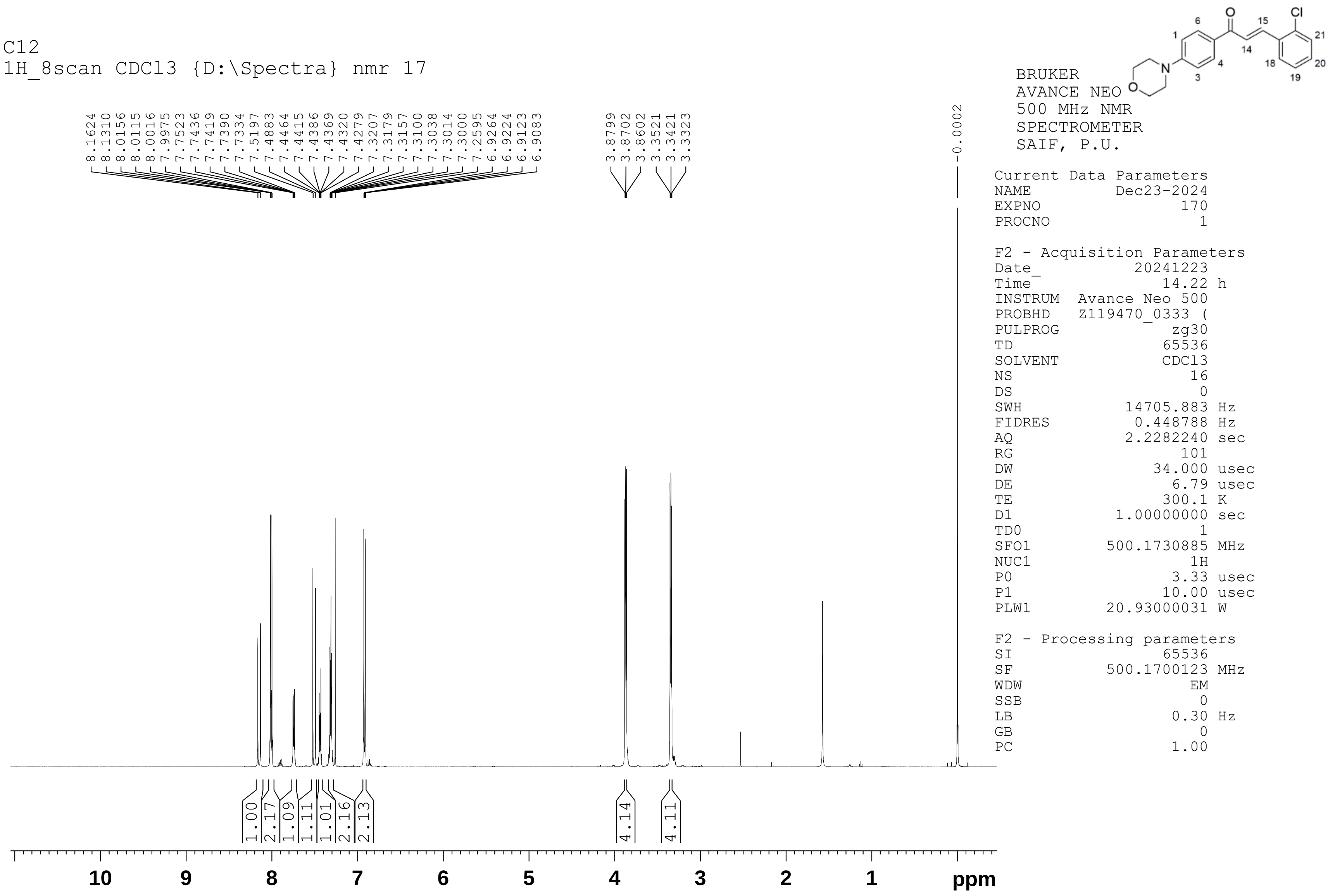

Figure S44. ^{1}H NMR of **C12**

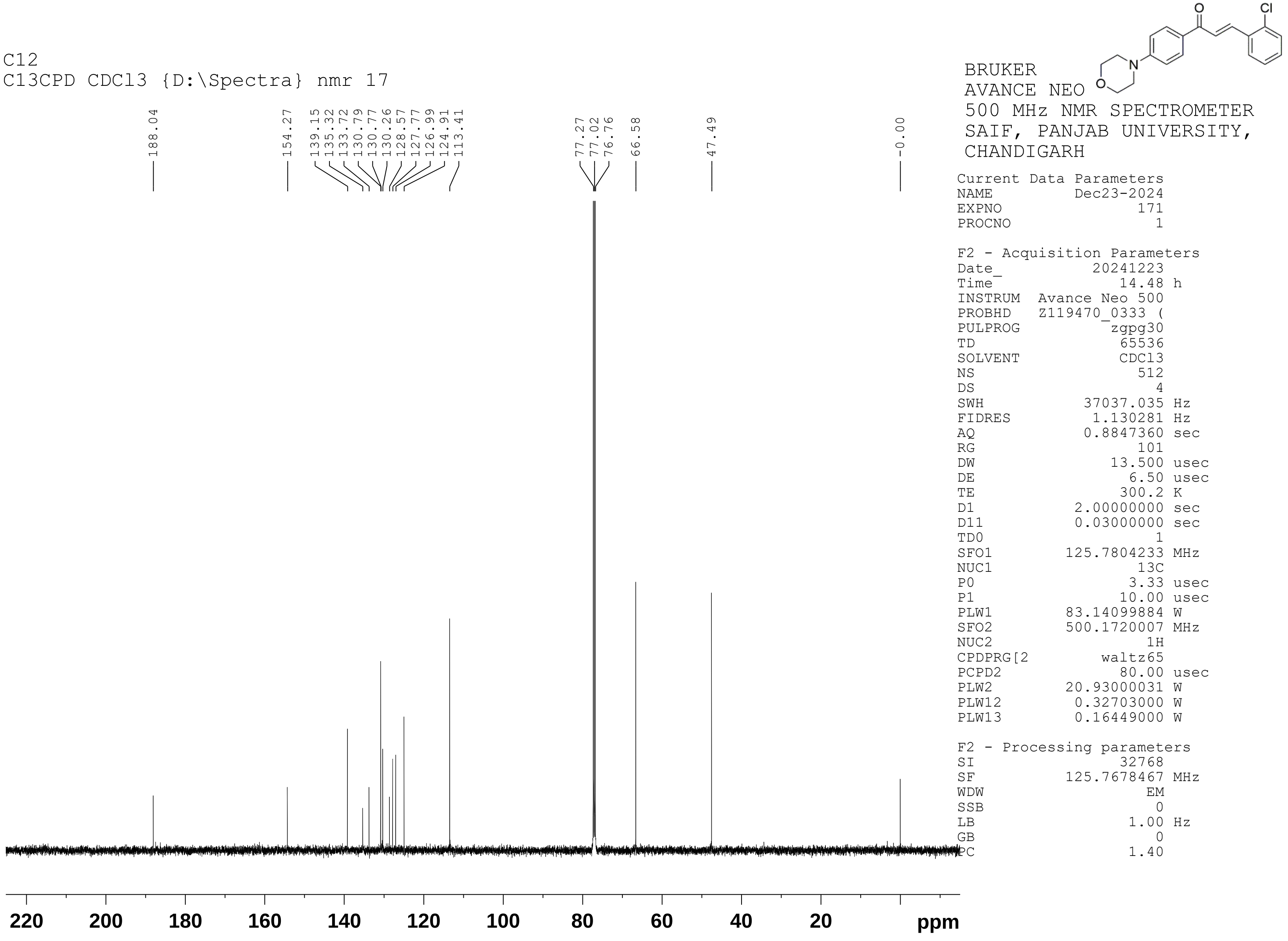

Figure S45. ^{13}C NMR of **C12**

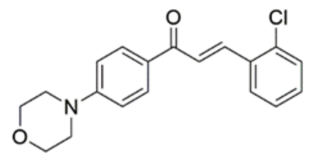

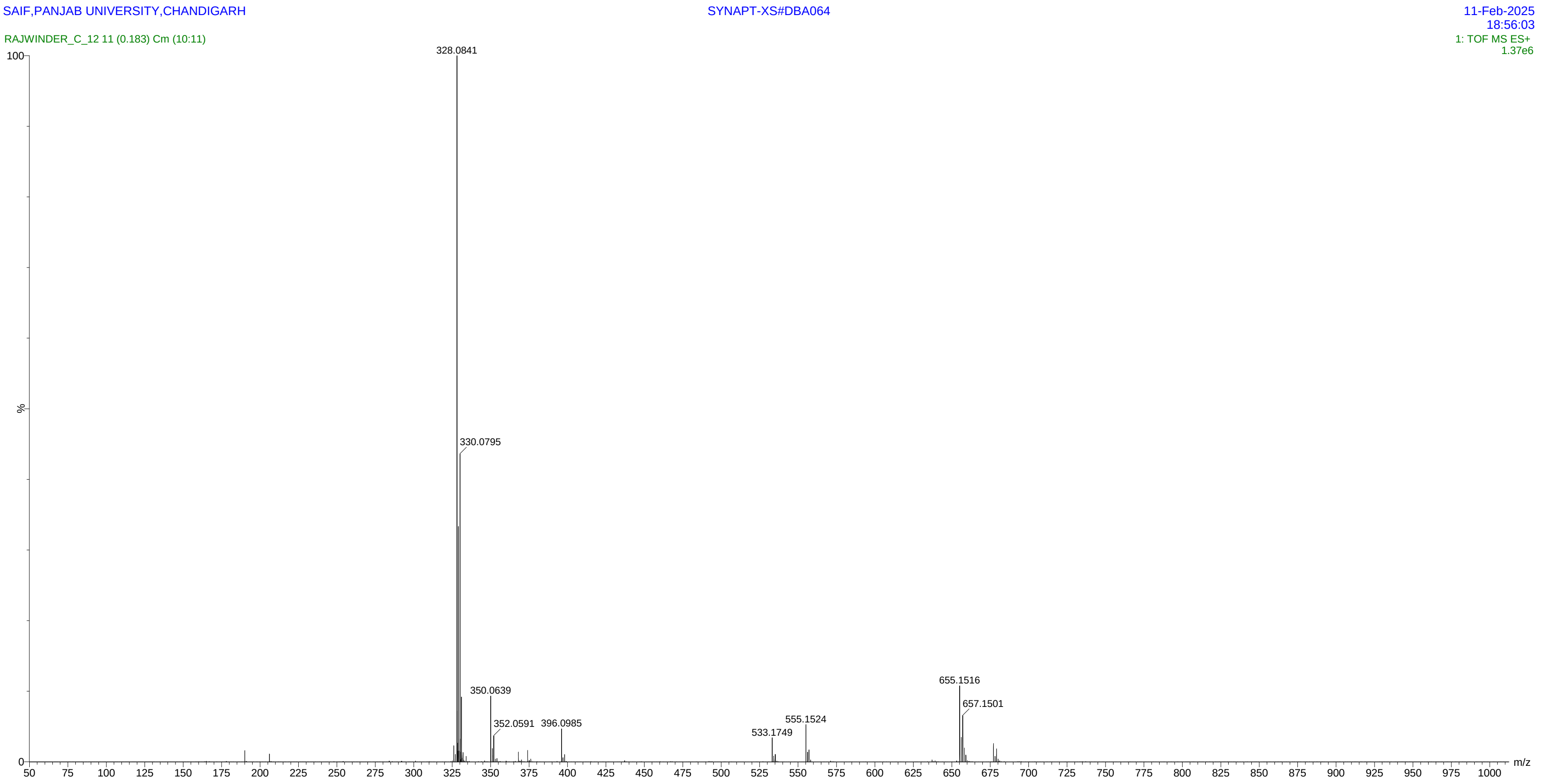

Figure S46. HRMS of **C12**

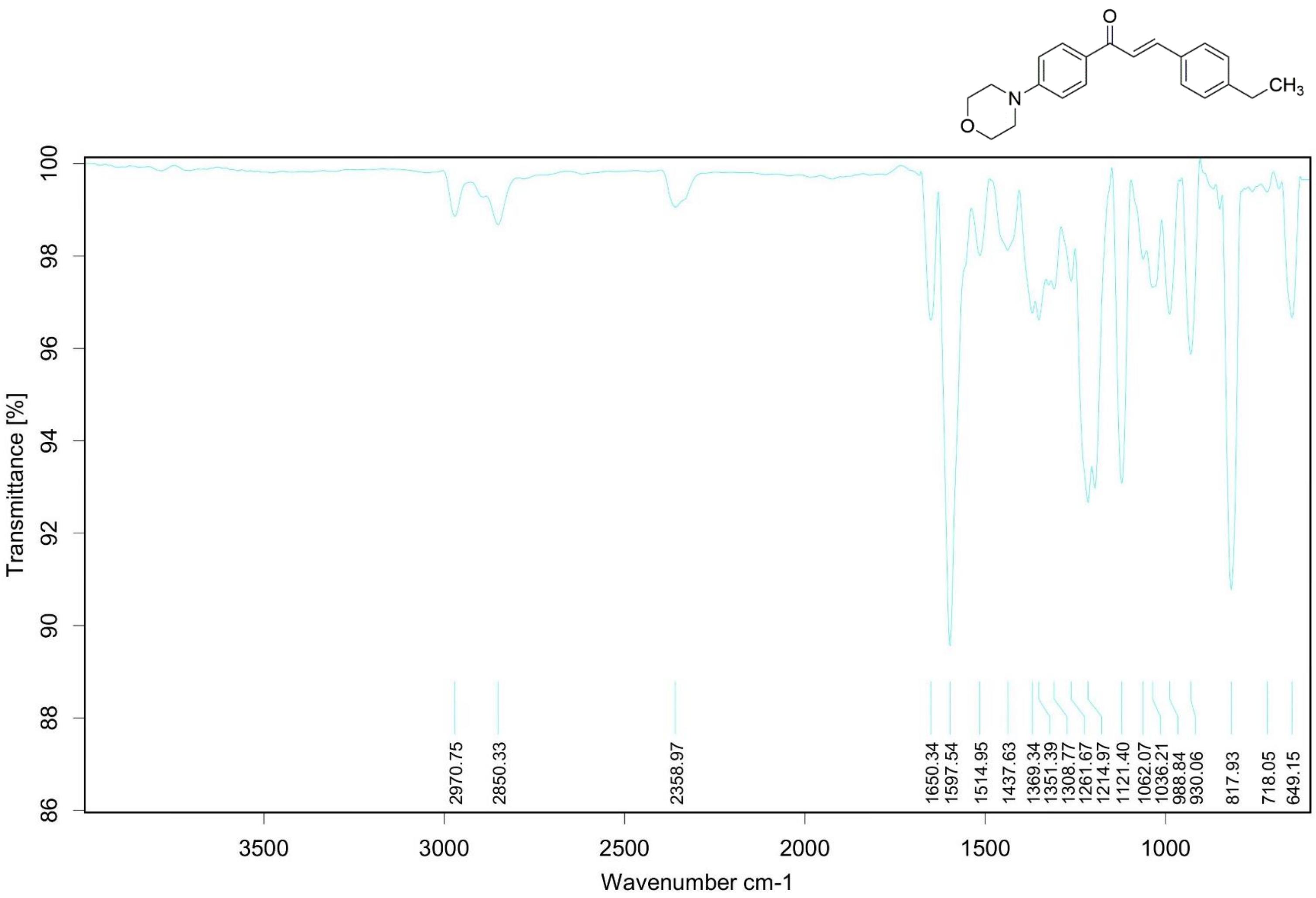

Figure S47. FT-IR of **C13**

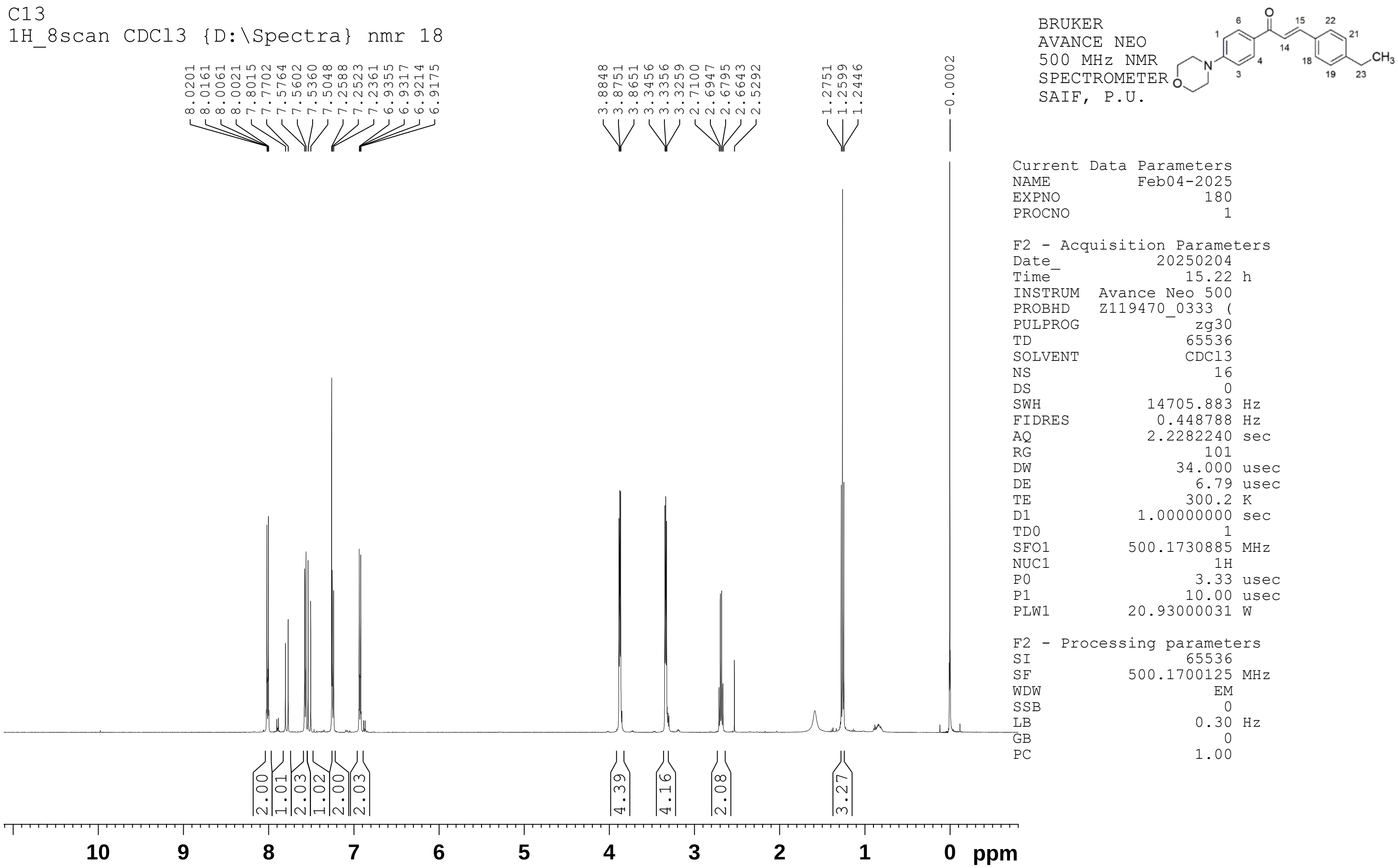

Figure S48. ^{1}H NMR of **C13**

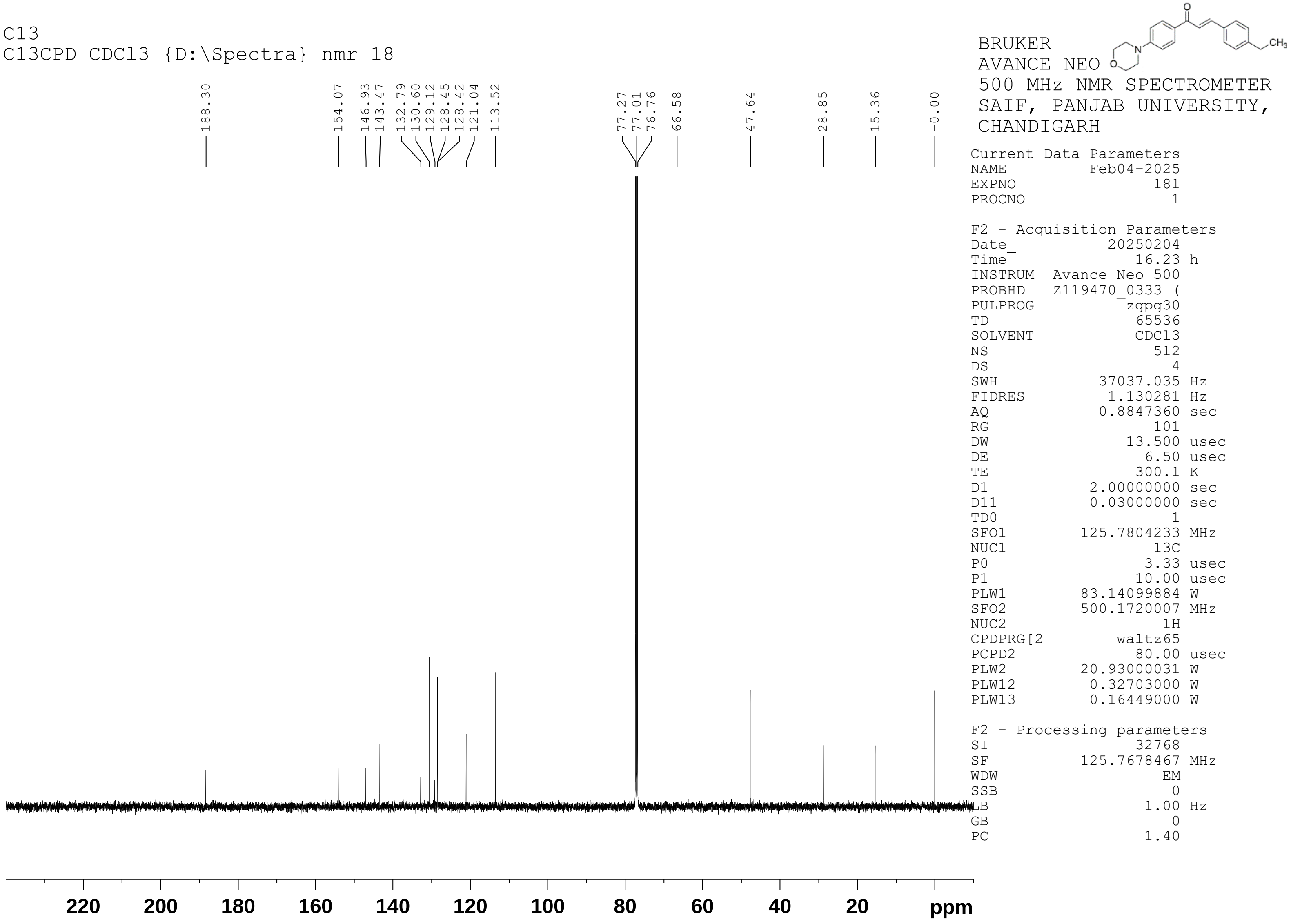

Figure S49. ^{13}C NMR of **C13**

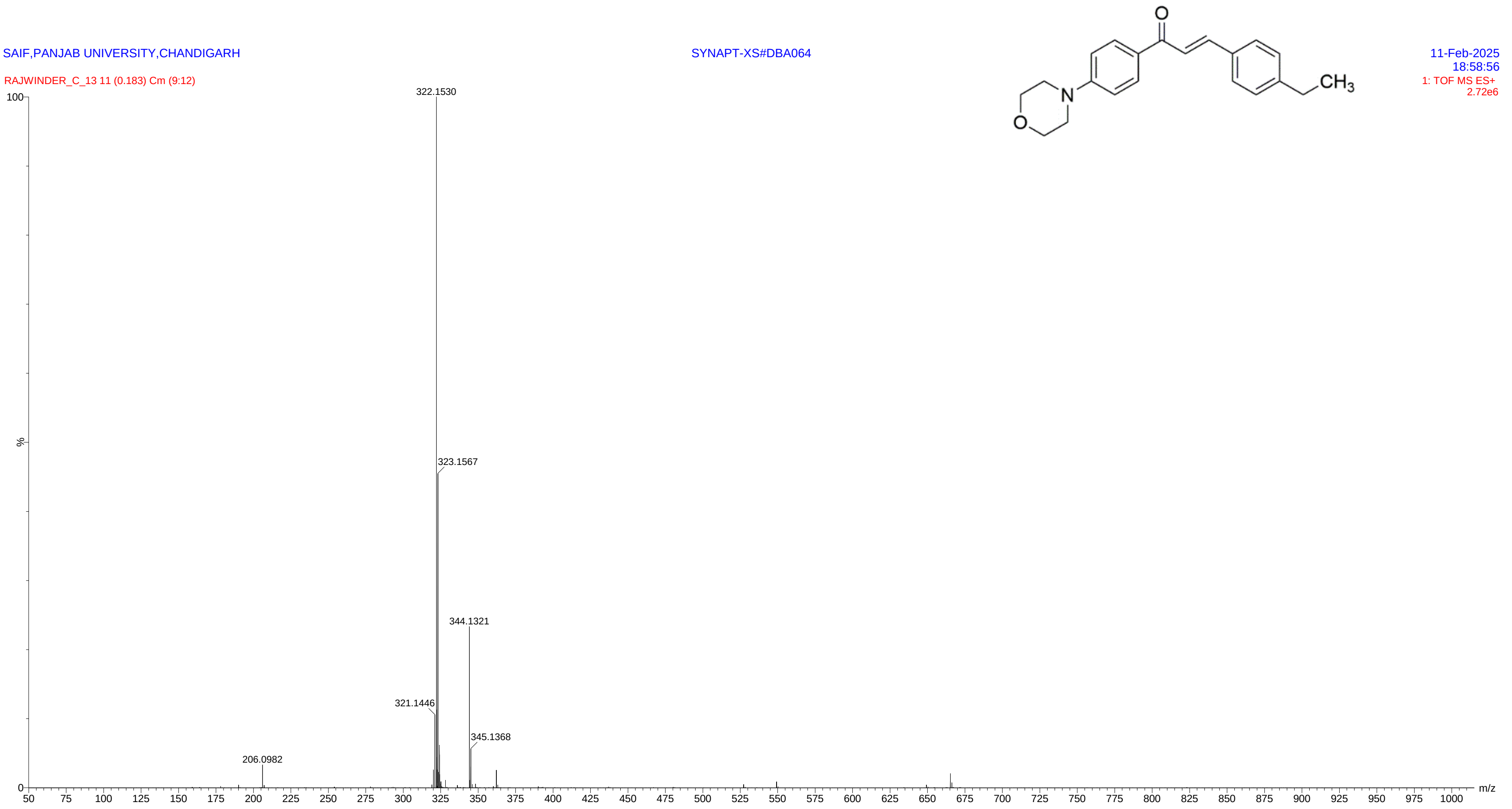

Figure S50. HRMS of **C13**

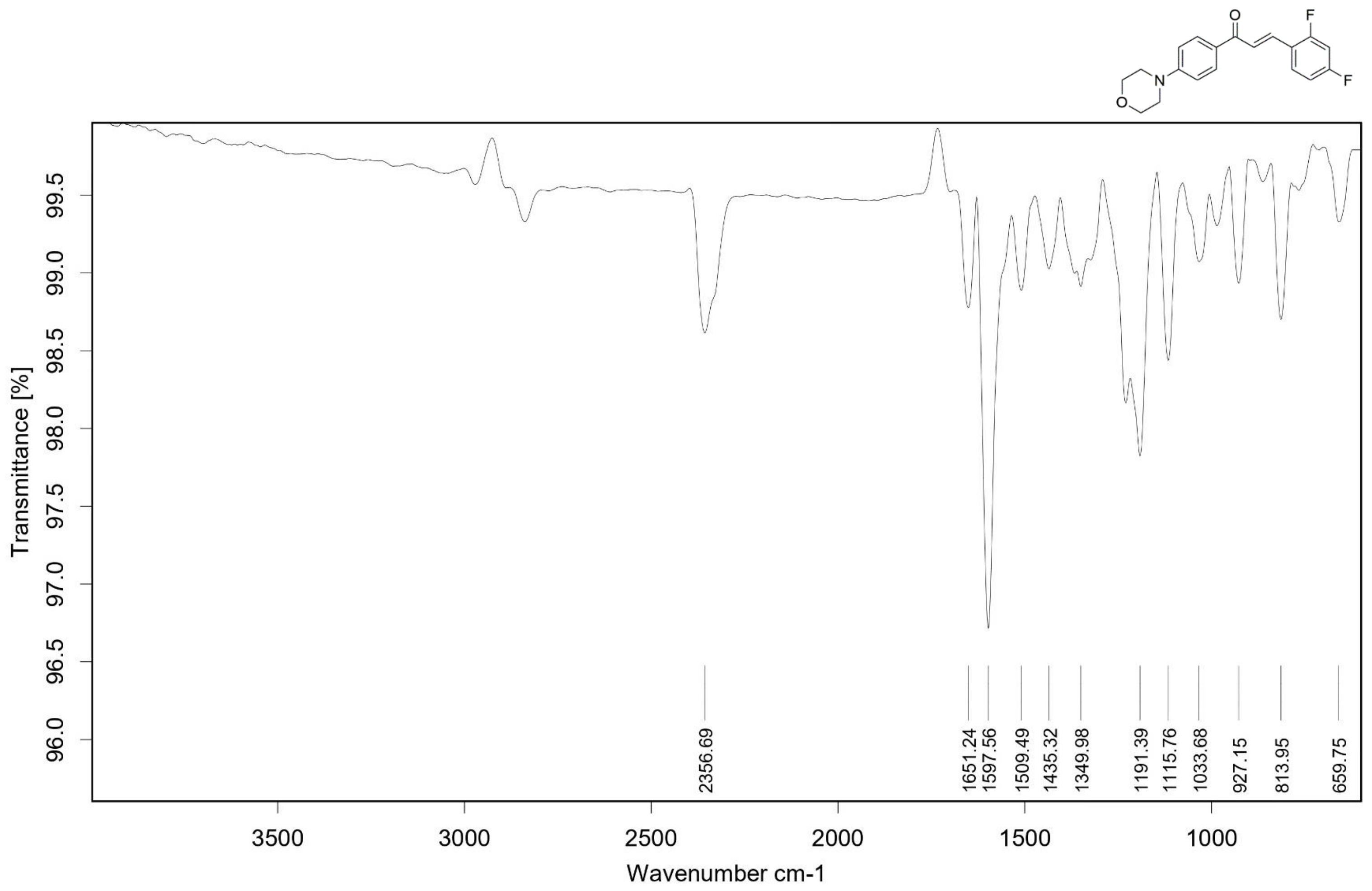

Figure S51. FT-IR of **C14**

C14
1H_8scan CDCl3 {D:\Spectra} nmr 18

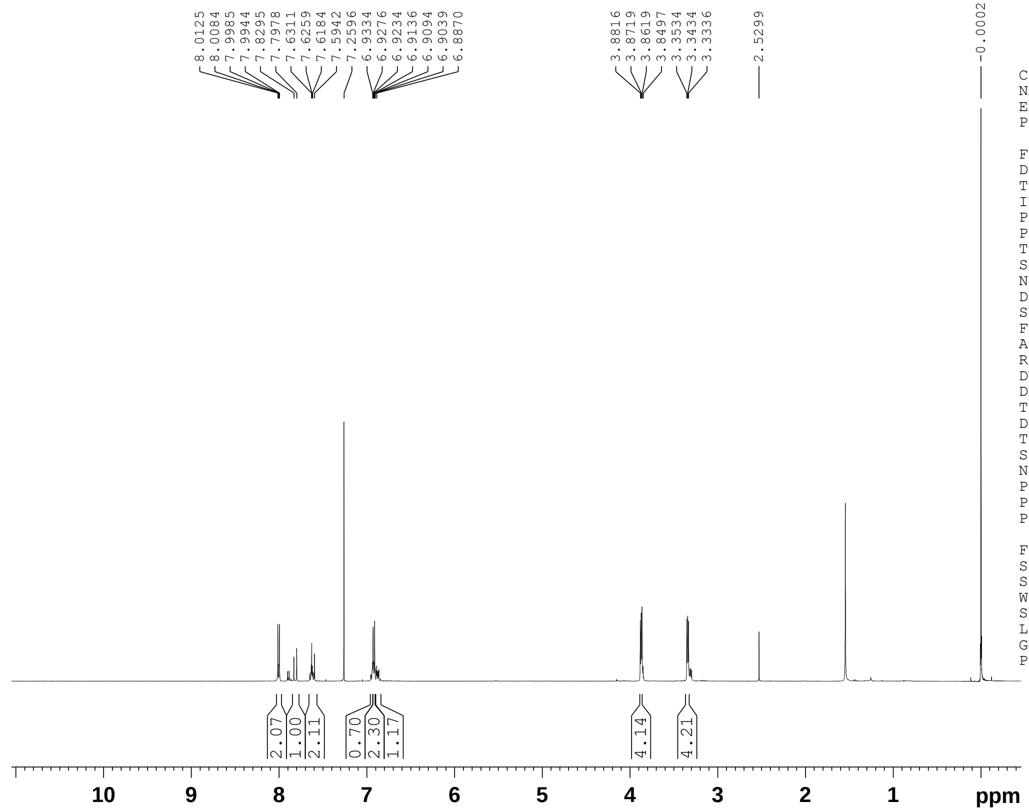

BRUKER
AVANCE NEO
500 MHz NMR
SPECTROMETER
SAIF, P.U.

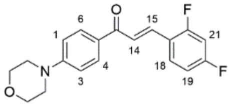

Current Data Parameters	
NAME	Dec23-2024
EXPNO	180
PROCNO	1

F2 - Acquisition Parameters	
Date_	20241223
Time	14.51 h
INSTRUM	Avance Neo 500
PROBHD	Z119470_0333 (
PULPROG	zg30
TD	65536
SOLVENT	CDCl3
NS	16
DS	0
SWH	14705.883 Hz
FIDRES	0.448788 Hz
AQ	2.2282240 sec
RG	101
DW	34.000 usec
DE	6.79 usec
TE	300.1 K
D1	1.00000000 sec
TD0	1
SFO1	500.1730885 MHz
NUC1	1H
P0	3.33 usec
P1	10.00 usec
PLW1	20.93000031 W

F2 - Processing parameters	
SI	65536
SF	500.1700123 MHz
WDW	EM
SSB	0
LB	0.30 Hz
GB	0
PC	1.00

Figure S52. ^{1}H NMR of **C14**

C14
C13CPD CDCl3 {D:\Spectra} nmr 18

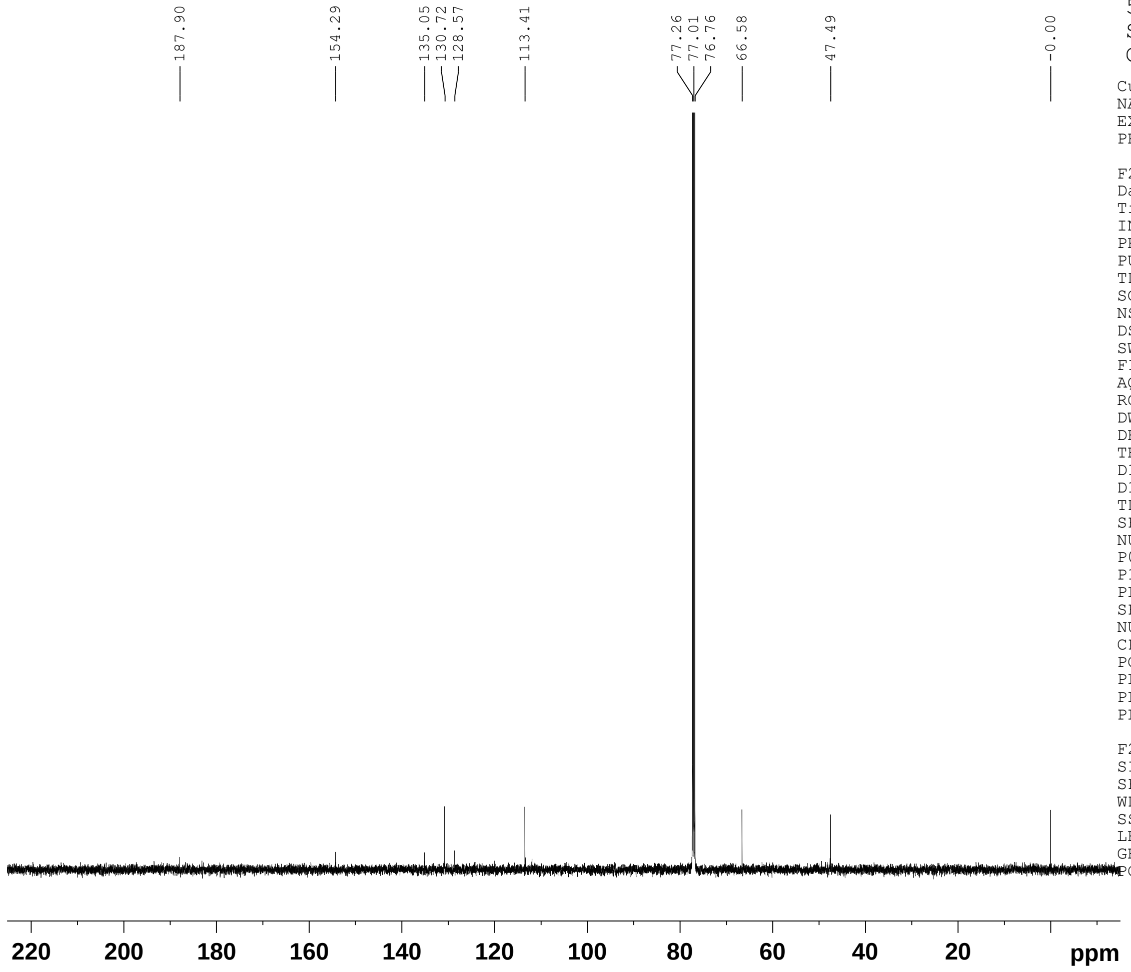

BRUKER
AVANCE NEO
500 MHz NMR SPECTROMETER
SAIF, PANJAB UNIVERSITY,
CHANDIGARH

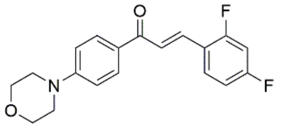


```
Current Data Parameters
NAME          Dec23-2024
EXPNO                181
PROCNO                 1

F2 - Acquisition Parameters
Date_           20241223
Time               15.16 h
INSTRUM   Avance Neo 500
PROBHD   Z119470_0333 (
PULPROG           zgpg30
TD                 65536
SOLVENT            CDCl3
NS                   512
DS                     4
SWH            37037.035 Hz
FIDRES          1.130281 Hz
AQ             0.8847360 sec
RG                   101
DW                13.500 usec
DE                  6.50 usec
TE                 300.1 K
D1            2.00000000 sec
D11           0.03000000 sec
TD0                    1
SFO1         125.7804233 MHz
NUC1                 13C
P0                  3.33 usec
P1                 10.00 usec
PLW1         83.14099884 W
SFO2         500.1720007 MHz
NUC2                  1H
CPDPRG[2         waltz65
PCPD2              80.00 usec
PLW2         20.93000031 W
PLW12         0.32703000 W
PLW13         0.16449000 W

F2 - Processing parameters
SI                 32768
SF           125.7678466 MHz
WDW                   EM
SSB                    0
LB                  1.00 Hz
GB                     0
PC                  1.40
```

Figure S53. ^{13}C NMR of **C14**

Manuscript ID: **pharmaceuticals-3444840**

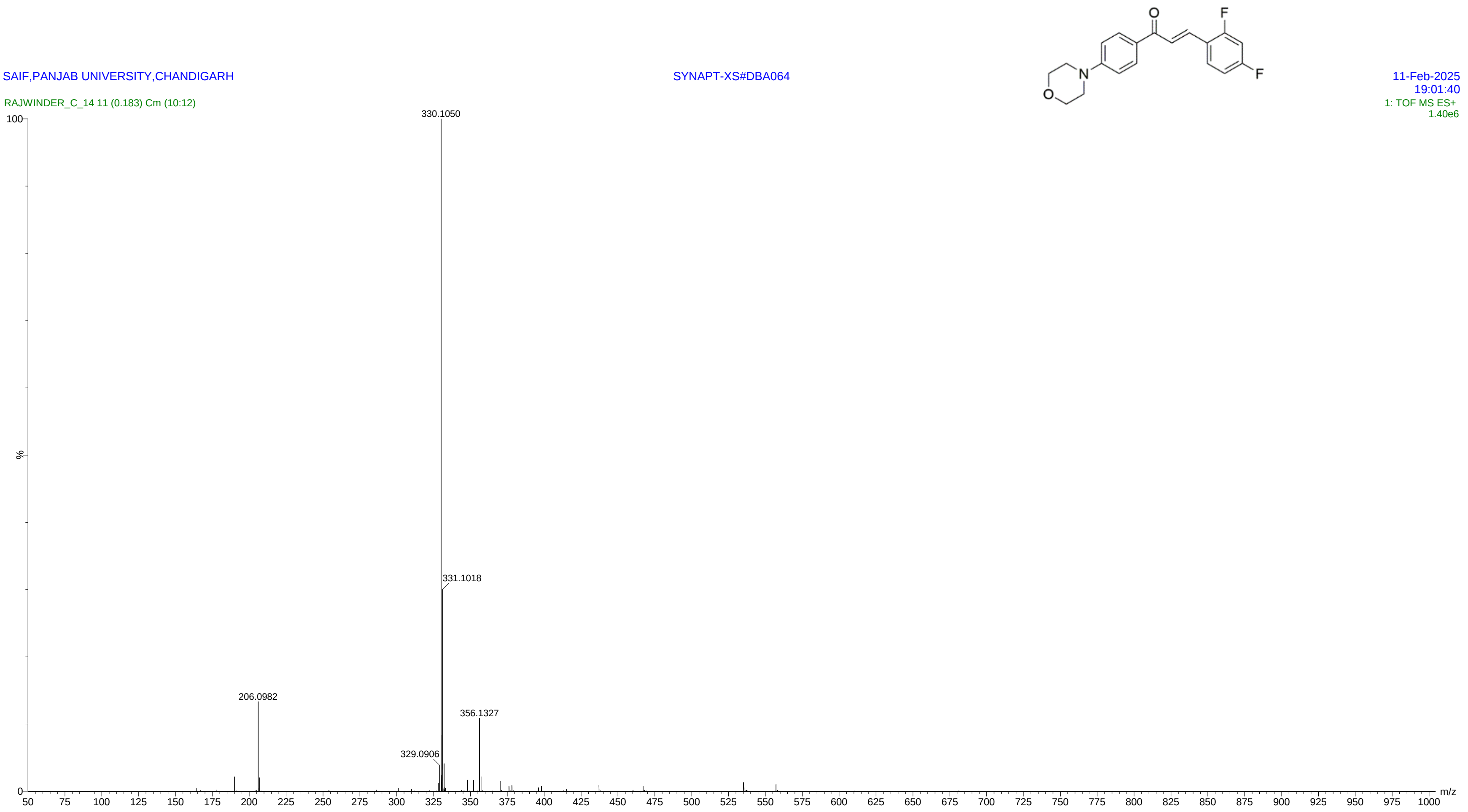

Figure S54. HRMS of **C14**

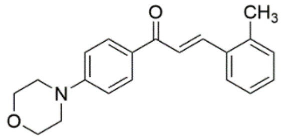

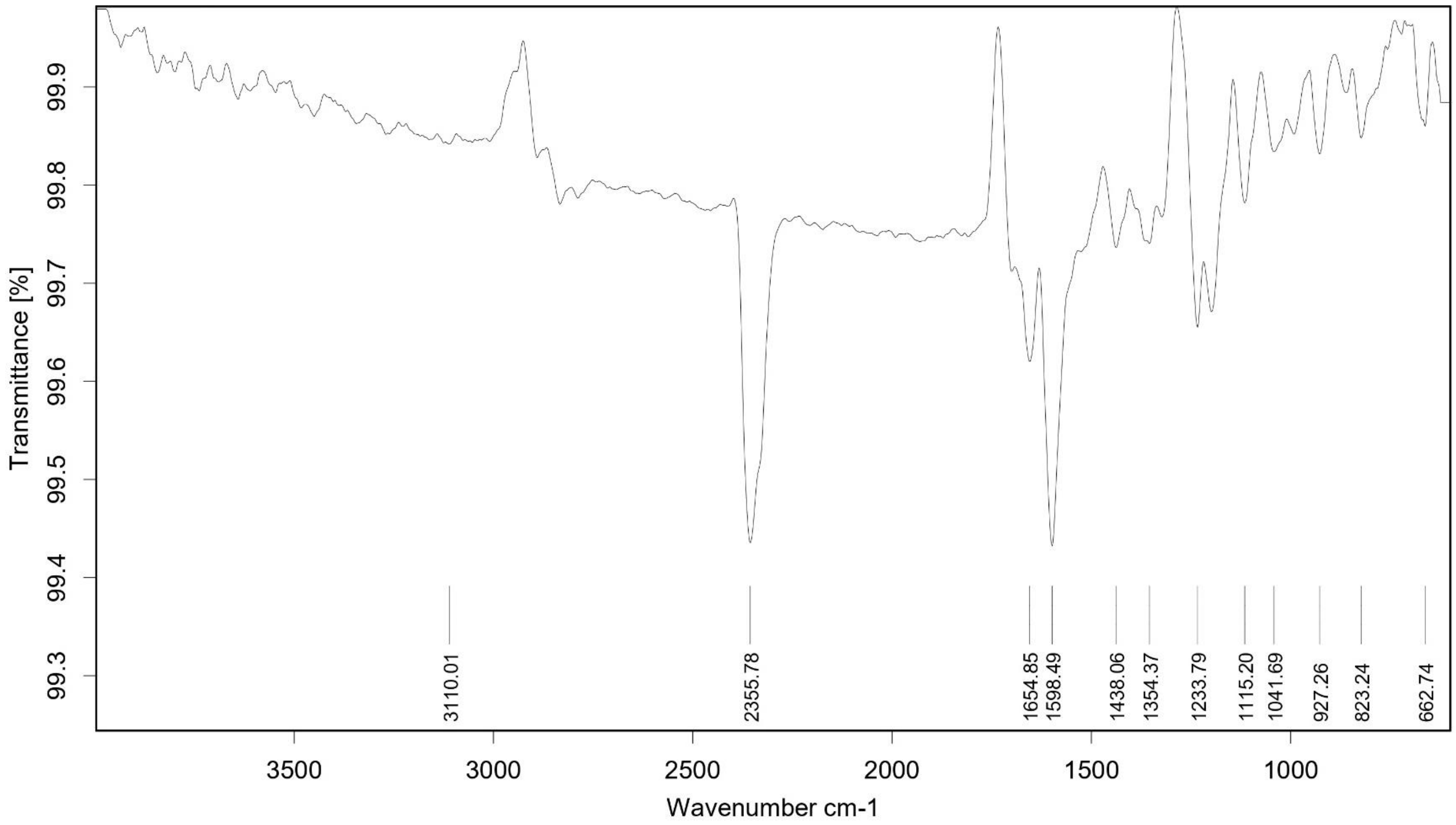

Figure S55. FT-IR of **C15**

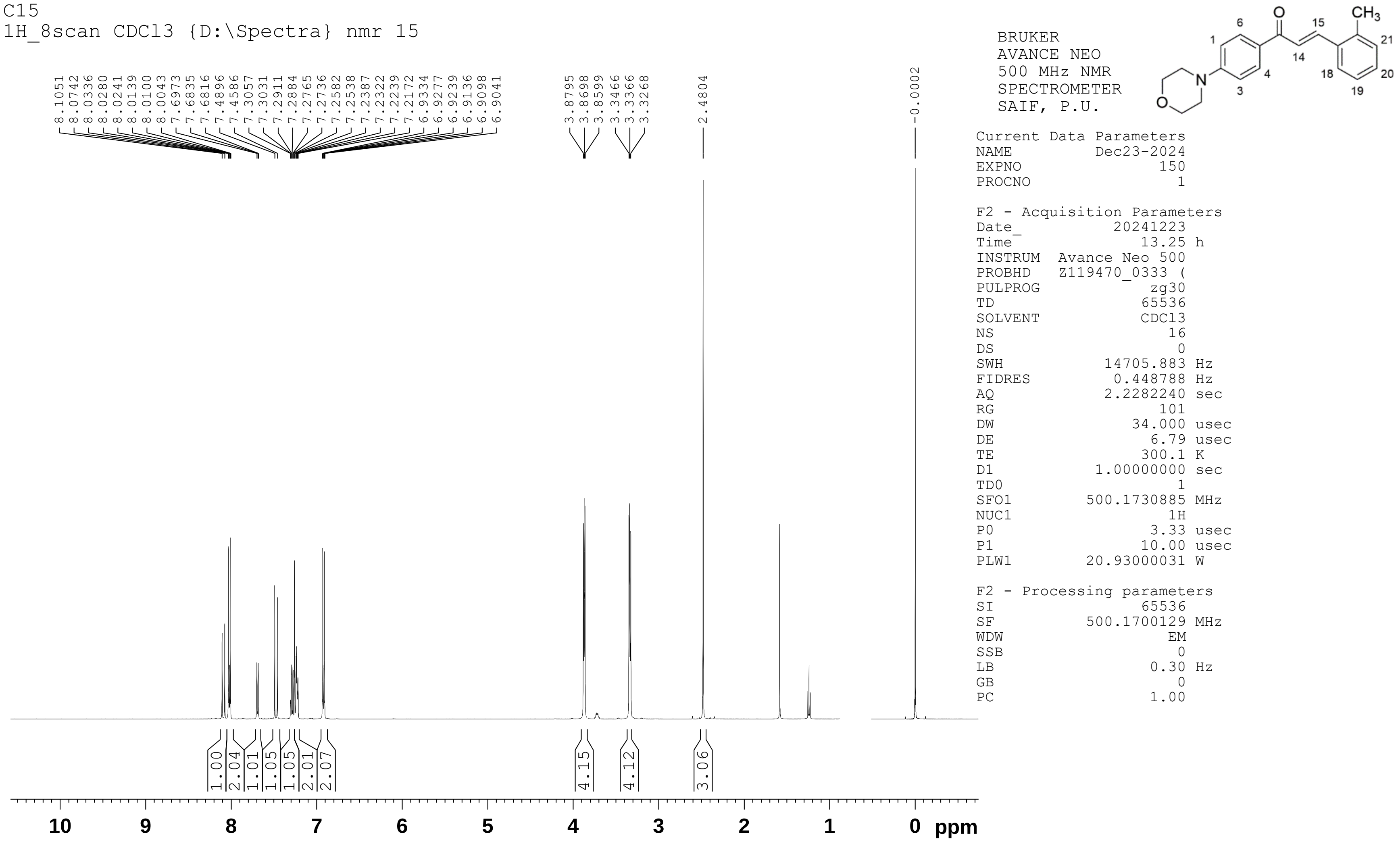

Figure S56. ^{1}H NMR of **C15**

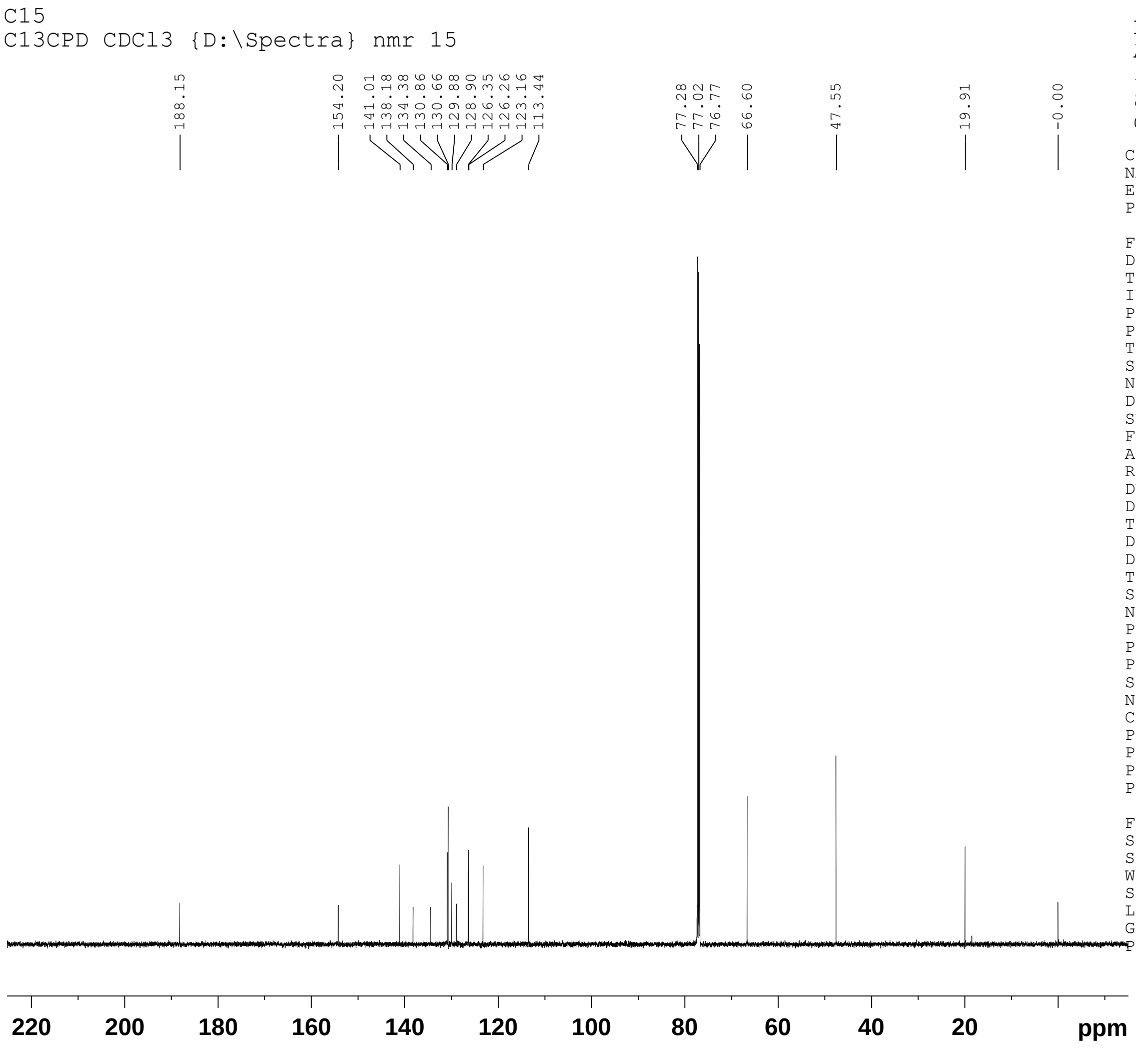

BRUKER
AVANCE NEO
500 MHz NMR SPECTROMETER
SAIF, PANJAB UNIVERSITY,
CHANDIGARH

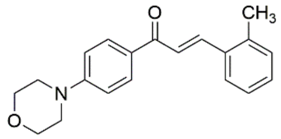


```
Current Data Parameters
NAME          Dec23-2024
EXPNO                151
PROCNO                 1

F2 - Acquisition Parameters
Date_           20241223
Time               13.51 h
INSTRUM  Avance Neo 500
PROBHD   Z119470_0333 (
PULPROG           zgpg30
TD                 65536
SOLVENT            CDCl3
NS                   512
DS                     4
SWH            37037.035 Hz
FIDRES          1.130281 Hz
AQ             0.8847360 sec
RG                   101
DW                13.500 usec
DE                  6.50 usec
TE                 300.2 K
D1            2.00000000 sec
D11           0.03000000 sec
TD0                    1
SFO1         125.7804233 MHz
NUC1                 13C
P0                  3.33 usec
P1                 10.00 usec
PLW1         83.14099884 W
SFO2         500.1720007 MHz
NUC2                  1H
CPDPRG[2         waltz65
PCPD2              80.00 usec
PLW2         20.93000031 W
PLW12         0.32703000 W
PLW13         0.16449000 W

F2 - Processing parameters
SI                 32768
SF           125.7678467 MHz
WDW                   EM
SSB                    0
LB                  1.00 Hz
GB                     0
PC                  1.40
```

Figure S57. ^{13}C NMR of **C15**

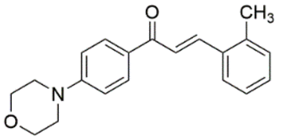

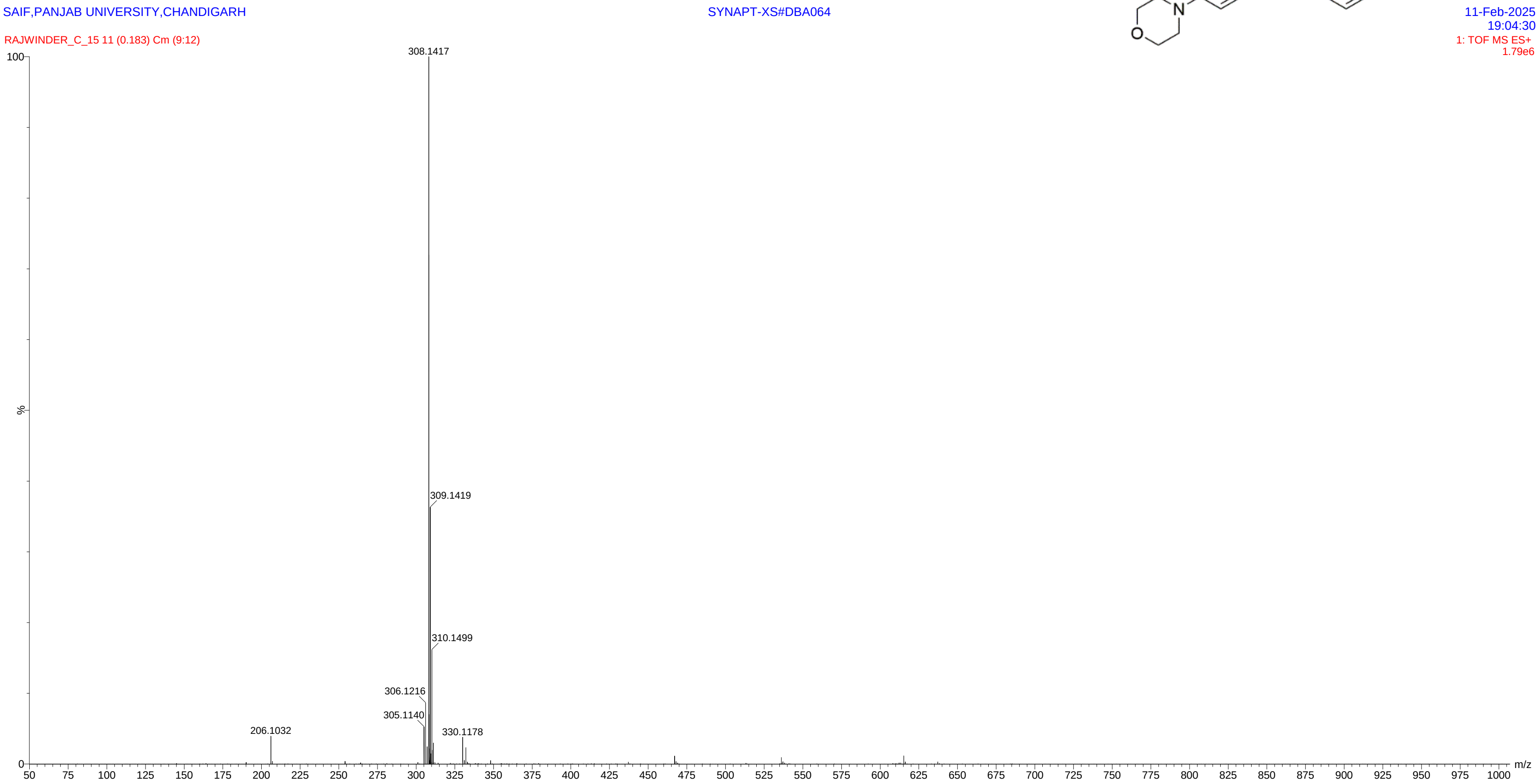

Figure S58. HRMS of **C15**
